# Live-cell magnetic manipulation of recycling endosomes reveals their direct effect on actin protrusions to promote invasive migration

**DOI:** 10.1126/sciadv.adu6361

**Published:** 2025-07-04

**Authors:** Jakub Gemperle, Domenik Liße, Marie Kappen, Emilie Secret, Mathieu Coppey, Martin Gregor, Christine Menager, Jacob Piehler, Patrick Caswell

**Affiliations:** ^1^ Manchester Cell-Matrix Centre, School of Biological Sciences, Faculty of Biology Medicine and Health, Manchester Academic Health Science Centre, The University of Manchester, Manchester, UK.; ^2^ Laboratory of Integrative Biology, Institute of Molecular Genetics of the Czech Academy of Sciences, Prague, Czech Republic.; ^3^ Department of Biology/Chemistry and Center of Cellular Nanoanalytics, Osnabrück University, Osnabrück, Germany.; ^4^ MLM Medical Labs GMBH, Mönchengladbach, Germany.; ^5^ Physico-chimie des Électrolytes et Nanosystèmes Interfaciaux, PHENIX, Sorbonne Université, CNRS, Paris, France.; ^6^ Laboratoire Physico Chimie Curie, Institut Curie, Paris, France.

## Abstract

Endocytic recycling pathways play key roles in a broad range of cellular processes, and many vesicle trafficking regulators are implicated in progression of disease such as cancer. The Rab11 family (Rab11a, Rab11b, and Rab25) controls the return of internalized cargos to the plasma membrane, and Rab25 has been implicated in the aggressiveness of cancer by promoting invasive migration. However, while Rab25 vesicles distribute to the leading edge of moving cells, how directly they contribute to cell protrusion is not clear. Here, we adopt a magnetogenetic approach that allows direct manipulation of Rab25 positioning to show that localization to the cell periphery drives the formation of F-actin protrusions. We demonstrate that endogenous Rab25 vesicles coordinate the positioning of key cargos, including the actin regulator FMNL1 and integrin β1, with the activation of Rho guanosine triphosphatases at the plasma membrane to generate and maintain F-actin–rich filopodium-like protrusions and promote cancer cell invasive migration in the three-dimensional matrix.

## Teaser

Repositioning of Rab25 vesicles containing formin and integrin cargos to the cell periphery promotes protrusion formation.

## INTRODUCTION

Endocytic trafficking controls how cells sense and respond to their environment (*1*, *2*), and accumulating evidence suggests a fundamental role in many physiological processes and pathological conditions (*3*–*5*). Rab guanosine triphosphatases (GTPases) control specific steps within the intricate vesicular trafficking pathways, including how endosomes are positioned. It has been proposed that specific localization of endosomes contributes to polarization, local growth, and migration of cells, either through the selective delivery of receptors or through localized signaling (*3*, *6*–*9*). However, it is challenging to directly address the functional consequences of endosomal positioning, and it remains unclear whether Rab GTPases simply position vesicles and their receptor cargos in a local area for utilization at the membrane or whether they play an active role in determining the initiation of protrusions.

The Rab11 subfamily, composed of Rab11a, Rab11b, and Rab25 (R25; also known as Rab11c), are key regulators of endocytic recycling. They control the return of internalized membrane-associated cargos to the cell surface (*10*) through recycling endosomes. Accumulating evidence suggests that polarized Rab11 trafficking contributes to aspects of tumorigenicity (*5*, *11*–*16*). Furthermore, Rab11 recycling endosomes have been demonstrated to nucleate F-actin from vesicles by recruiting actin nucleation factors to organize the actin network into tracks that allow for microtubule-independent vesicle movement (*17*, *18*). The Rab11 family has also been linked to cell motility. Rab11a/b has been demonstrated to facilitate cancer cell invasion by promoting the formation of filopodium-tipped protrusions (*19*–*22*), suggesting a link between the Rab11 family and F-actin nucleation that extends beyond formation of tracks for vesicles. Unlike the ubiquitous Rab11s (Rab11a and Rab11b), Rab25 has a limited expression profile in normal tissue (*23*) and has been shown to be up-regulated in approximately half of ovarian and breast tumors, correlating with their aggressiveness both clinically and in mouse models (*24*, *25*). Rab25 directs the localization of integrin α5β1–containing recycling vesicles/endosomes to the leading edge of migrating cells to promote the formation of long actin-rich pseudopodia and enhance the ability of tumor cells to invade the extracellular matrix (*11*, *21*, *26*).

While it is clear that recycling vesicles serve to deliver transmembrane cargos such as integrins to the plasma membrane (PM) at the leading edge to facilitate cell migration, and the presence of recycling endosomes correlates with protrusion formation, whether recycling endosomes directly modulate F-actin regulation at the cell periphery to promote protrusion formation is not known.

The causal relationship between the directed localization of recycling endosomes and cell invasion could be dissected through active manipulation of their repositioning. Optogenetic control of Rab11 endosomes provided valuable insight into the role of Rab11 in neurite outgrowth in two dimensions (*27*). However, this approach depends on recruiting engineered cytoskeletal motor proteins such as kinesin, myosin, or dynein, which are guided by an endogenous, polarized cytoskeleton. Blue light phototoxicity, inadvertent background dark-state binding, and motor overexpression side effects are also confounding factors for this approach (*28*, *29*). In contrast to optogenetics, magnetic manipulation techniques are readily compatible with opaque specimens and are noninvasive and not limited by molecular motor properties (*30*). Recent advances in magnetogenetics have led to the development of magnetic semisynthetic superparamagnetic nanoparticles combined with green fluorescent protein (GFP) that have appropriate biological, physicochemical, and magnetic properties. These nanoparticles have demonstrated the ability to manipulate proteins and organelles fused to anti-GFP nanobodies inside living cells under two-dimensional (2D) conditions with exceptional spatial and temporal resolution (*30*–*32*). Here, we have built on this approach to develop a magnetogenetic strategy to micromanipulate oncogenic Rab25 endosomes and vesicles with high spatiotemporal precision in migrating cells. We show that the local positioning of Rab25 vesicles to the PM is sufficient to initiate protrusion in 2D and physiologically relevant three-dimensional (3D) matrices. The actin-polymerizing protein FMNL1 (formin-like protein 1) is a cargo of Rab25 vesicles, which promotes the formation of both actin tracks and filopodia to generate nascent protrusions, and Rab25 acts to coordinate the delivery of FMNL1 and integrins with RhoA activity to promote protrusion formation.

## RESULTS

### Magnetogenetic manipulation of endosome positioning directly drives cell protrusions

To gain insight into the role of Rab25 in cancer cell invasion, we reactivated expression of Rab25 from the genomic locus of A2780 ovarian cancer cells using the DExCon approach (fig. S1A) (*21*). This allows for the reactivation of silenced Rab25 gene in a doxycycline (dox)–dependent manner and enables the visualization of endogenous Rab25-positive endosomes using mCherry fluorescence. The anti-GFP nanobody (NB^GFP^) was included in frame with endogenous Rab25 to enable vesicle manipulations (fig. S1, A to C). The functionality of NB^GFP^ was verified through colocalization with coexpressed GFP (fig. S1C), with induced Rab25 showing the expected perinuclear vesicle–enriched localization. Rab25 expression increased cell length, consistent with previous observations (*11*), thereby demonstrating the functionality of our system (fig. S1, D and E). In addition, we observed a positive correlation between the trafficking of Rab25 endosomes toward the PM and actin polymerization/protrusion growth (see fig. S1, F and G). Similarly, actin tracks were observed alongside Rab25 endosomes (fig. S1H), which also formed independently of microtubules (fig. S1I). These findings corroborate previous research (*11*, *17*, *19*) but do not distinguish the causality between endosome positioning and actin polymerization/protrusion formation.

**Fig. 1.**
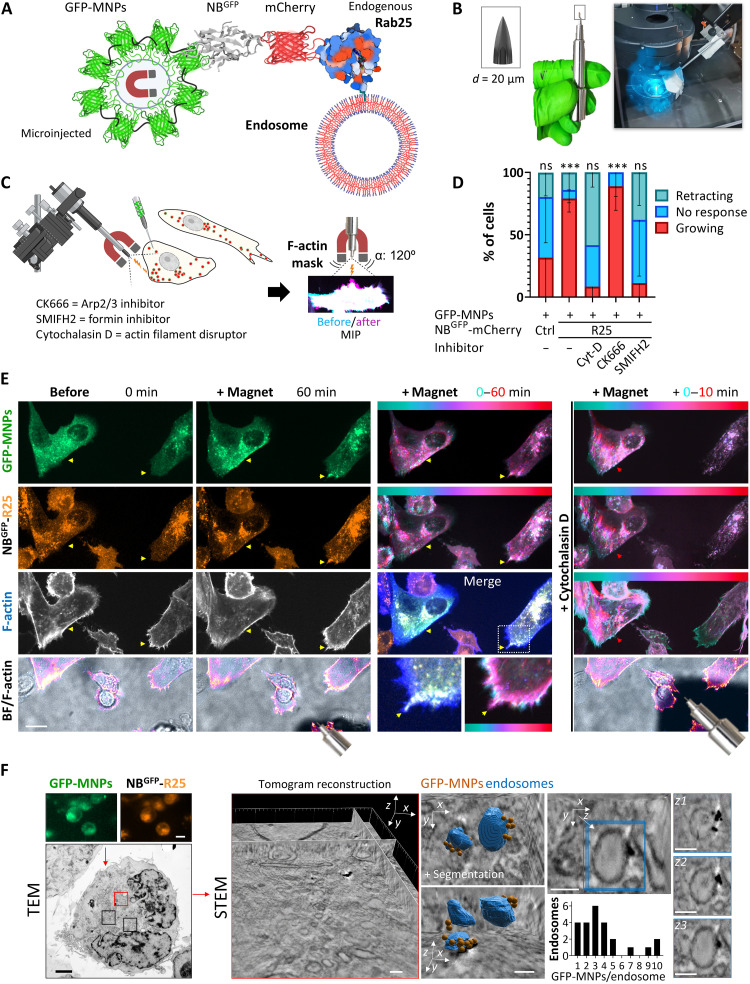
A magnetogenetic approach for remote manipulation of Rab25 (R25) demonstrates a direct role for endosome positioning in cell protrusion growth. (**A**) Schematic diagram of the magnetogenetic strategy to control R25 endosomes. (**B**) Magnetic tip assembly composed of neodymium magnets and a fine wire tip attached to the micromanipulation apparatus. (**C**) Experimental setup and (**D**) quantification of protrusion growth: GFP-MNPs delivered by microinjection into randomly selected (independent of initial cell polarity) A2780 cells expressing control NB^GFP^-mCherry-(Ctrl) or NB^GFP^-mCherry-R25 cells (DExCon-modified; dox for >94 hours; 250 ng/ml) on FN-coated coverslips and MNPs attracted with a magnetic tip. For each GFP-MNP–positive cell protrusion, growth toward the magnetic tip was analyzed from maximum intensity projections (MIPs) of Lifeact-iRFP670 (F-actin) images. Overlay masks before (0) and after (10 to 60 min) visible magnetic enrichment of GFP-MNPs, *n* = 19 (Ctrl; *N* = 5), *n* = 27 (R25; *N* = 8), *n* = 9 [R25; 200 nM cytochalasin D (Cyt-D); *N* = 2], *n* = 8 (R25; 100 μM CK666; *N* = 3), *n* = 13 (R25; 5 μM SMIFH2, *N* = 3), means ± SD. Ordinary one-way ANOVA (GraphPad); ***P < 0.001. ns, not significant. (**E**) Representative spinning-disk confocal time-lapse images of cells with magnetically attracted R25 endosomes before and after cytochalasin D treatment (see movie S2). The shadow in the bright field (BF) indicates the magnetic tip. Yellow arrowheads and cyan-red LUT illustrate changes in GFP-MNPs and vesicle distribution and protrusion growth/filopodia (F-actin) over time through color grading. Scale bar, 20 μm. (**F**) STEM tomography. GFP-MNPs delivered by electroporation (representative fluorescence images; scale bar, 10 μm); TEM image (scale bar, 2 μm) with a corresponding STEM tomogram (red box; scale bar, 2 μm); zoom (right) with a segmented tomogram and quantification of GFP/MNPs per recycling endosome (50 to 200 nm) (scale bar, 100 nm). Tomogram animation movie S16 is accessible via https://doi.org/10.6084/m9.figshare.22155083. [(A) and (C)] Created in BioRender (https://biorender.com/6wqgouf).

To determine the precise connection and causality between Rab25 recycling endosomes and protrusion growth, we developed a magnetogenetic approach for the spatiotemporal control of Rab25 endosomes (Fig. 1A) using a magnetic tip attached to a remote-controlled micromanipulator (Fig. 1B). A2780 cells were microinjected with the superparamagnetic synthetic maghemite core particles coated with GFP [monomeric enhanced GFP (mEGFP)] fused with the iron binding site of Mms6 from magnetotactic bacteria [GFP-coated magnetic nanoparticles (GFP-MNPs); Fig. 1, A to C] (*32*). We previously found that these nanoparticles yield intracellular stealth properties and high magnetization while also maintaining a small hydrodynamic diameter (19.6 ± 2.7 nm), allowing them to diffuse freely in the cytoplasm (*32*). To enhance the usability and stability of the mEGFP coating (*32*), mEGFP was cross-linked with paraformaldehyde (PFA) after binding to nanoparticles. A rapid attraction gradient of freely moving GFP-MNPs toward the magnetic field was observed in all microinjected cells up to ~200 μm away from the micromagnet covering the 120° angle (fig. S2A). The magnetic force exerted on particles at a distance of 30 to 150 μm from the micromagnet was in the femtonewton range, as previously determined using the Boltzmann law from the steady-state profile of GFP-MNP distribution inside living cells (fig. S2B). By moving the micromagnet, it was possible to quantitatively concentrate GFP-MNPs to various subcellular, even centrally located positions in the cell (fig. S2C and movie S1). Although GFP-MNPs have unhindered mobility in the cytoplasm (*32*), they could not pass through the nuclear envelope and the nucleus provided an obstacle for their movement (fig. S2C and movie S1). Microinjection of GFP-MNPs into the cytoplasm of cells stably expressing NB^GFP^-mCherry (control) resulted in the accumulation of NB^GFP^-mCherry in the cytoplasm. Magnetic relocalization of both GFP-MNPs and NB^GFP^-mCherry was found to be reversible within a matter of seconds (attraction time *t*_1/2_ ~ 20 s; relaxation time *t*_1/2_ ~ 10 s) (fig. S2D) and did not have any visible effect on the PM or F-actin despite sustained attraction of GFP-MNPs over a 10- to 60-min imaging period (Fig. 1, C and D, and fig. S2, E and F).

Next, GFP-MNPs were microinjected into cells engineered to express NB^GFP^-mCherry-Rab25 from the endogenous locus. GFP-MNPs localized to Rab25 endosomes, which were then relocalized using magnets. The relocalization was noticeable within several minutes and increased slowly over time (Fig. 1E and movie S2). This suggests that the majority of endogenous Rab25 is likely to be vesicular and not rapidly diffusible, consistent with previous observations (*21*). The direct relocalization of Rab25 resulted in the formation of small but robust and clearly discernible actin-rich protrusions at sites where vesicles had accumulated. This occurred in the direction of the magnetic field in most microinjected cells, regardless of their polarity before the positioning of the magnet (Fig. 1, C to E, and movie S2). The effect of Rab25 on actin protrusions was blocked by cytochalasin D or the small-molecule inhibitor of formins (SMIFH2) but not by the Arp2/3 inhibitor CK666 (Fig. 1, C to E; movie S2; and fig. S3, A and B). This suggests that the mechanism is dependent on formin-mediated actin polymerization rather than Arp2/3. Unbiased artificial intelligence (AI)–based quantification of unmanipulated vesicle dynamics revealed that the active movement of Rab25 endosomes in A2780 cells does not require Arp2/3 activity, as demonstrated by the CK666 inhibitor after 5 min of treatment (fig. S4A). In contrast, inhibition of formins by SMIFH2 had a negative impact on the mobility of Rab25 vesicles in the absence of magnetic manipulation (fig. S4B). However, magnetic relocalization of Rab25 endosomes remained possible, although this did not result in any discernible effect on actin-based protrusions (fig. S4C). This is consistent with previously reported dose-dependent inhibition of A2780 cell migration by SMIFH2 (*33*).

To our knowledge, the number of Rab molecules per recycling endosome has not been well documented. Therefore, to determine the average magnetic force exerted on Rab25 recycling endosomes (40 to 200 nm in size) and compare it with the force parameters related to molecular motors, we imaged GFP-MNPs that were delivered to Rab25-positive cells using scanning transmission electron microscopy (STEM) tomography. In the reconstituted and segmented tomograms, we observed up to 10 GFP-MNPs per recycling endosome usually enriched in a defined subdomain of the outer vesicular membrane (Fig. 1F). This observation indicates that Rab25 may not be distributed uniformly throughout the recycling endosome. Furthermore, it suggests that the total force per whole Rab25 endosome is up to 15 fN (10 × 1.5 fN) at a distance of 50 μm from the micromagnet, which is two orders of magnitude below the proposed force of myosin and kinesin molecular motors (~1 to 5 pN) (*34*–*36*). Therefore, we hypothesized that a low but sustained magnetic force could alter the balance between positively and negatively directed motors and potentially repeatedly shift vesicles toward the micromagnet when they temporarily detach from the cytoskeleton. This is consistent with our previously published findings, which demonstrated relatively fast relocation of genetically modified Rab25 endosomes to the outer mitochondrial membrane using knock-sideways (*t*_1/2_ = 476 s) (*21*), and a recent observation that Rab11 vesicles move to a large extent by passive diffusion/flow (*37*). Overall, these data demonstrate that it is feasible to actively relocalize membrane-bound Rab25 using magnets and that relocalization of Rab25 directly triggers protrusion outgrowth that is F-actin– and formin-dependent.

### Endosomes spatiotemporally modulate Rab25 localization to control protrusion outgrowth

**Fig. 2.**
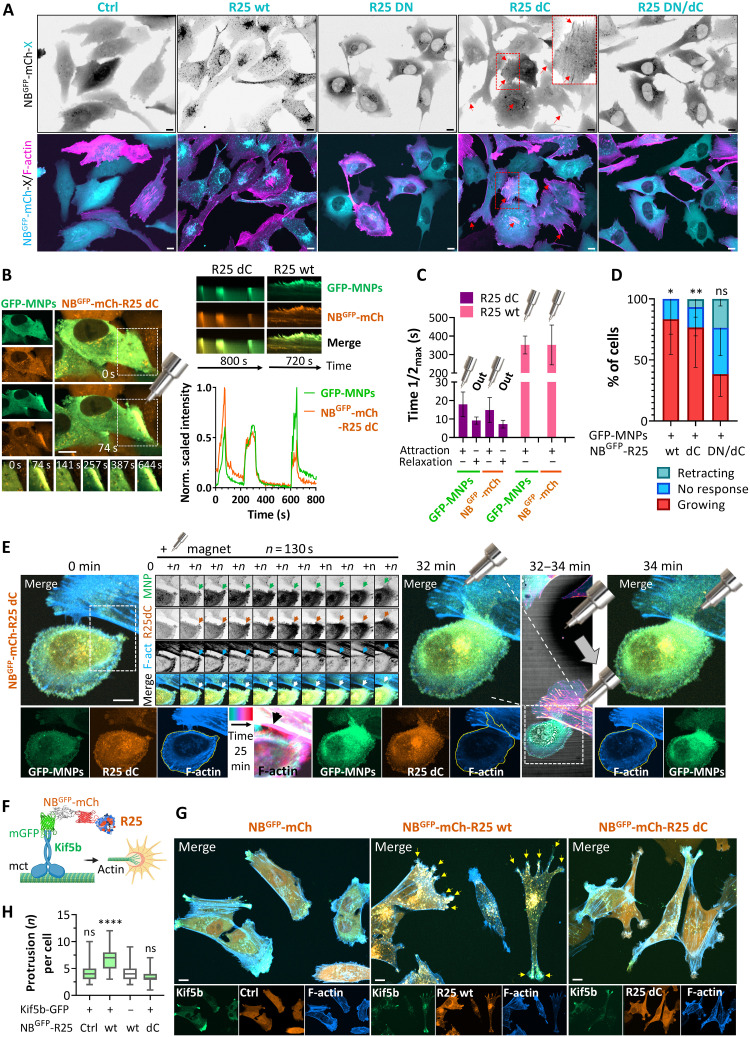
Endosomes spatiotemporally modulate R25 localization to control protrusion outgrowth. (**A**, **B**, and **E**) Representative images from confocal spinning-disk live-cell imaging of A2780 stably expressing Lifeact-iRFP670 (F-actin) with NB^GFP^-mCherry-(Ctrl) or fused with different R25 mutants on FN. (A) Boxed area ×2 magnified images. Arrows, stress fibers. [(B) and (C)] Magnetic attraction and release kinetics of NB^GFP^-mCherry-(X) and GFP-MNPs (delivered by microinjection); individual representative frames from the white box, kymographs and profiles determined from the changes in fluorescence intensity across the black arrow (normalized 0- to 1-scaled fluorescence intensities), exponentially fitted and quantified in (**C**); *n* = 3 (*N* = 3). (**D**) Protrusion growth quantified as in Fig. 1 (C and D); *n* = 14 (R25 wt; *N* = 3), *n* = 14 (R25 dC; *N* = 5), *n* = 22 (R25 DN/dC; *N* = 5), means ± SD. Ordinary one-way ANOVA. (E) Representative spinning-disk confocal time-lapse images (MIPs) of cells on FN with magnetically attracted NB^GFP^-mCherry-R25 dC; zoom inset from the white box (one-matched *Z* plane; arrows indicate changes). The shadow in the bright field indicates magnetic tip movement (gray arrow), and the corresponding alteration in cell shape is outlined by a yellow line. Color-grade time lapse (cyan-red LUT, arrow). See movie S3. (**F**) Schematic diagram of the alternative strategy to control R25 endosomes on the basis of the NB^GFP^ module and GFP-tagged C-terminally truncated molecular motor Kif5b[1-807] (created with BioRender.com, https://biorender.com/f9jfcq2). (**G**) Representative confocal spinning-disk live-cell images of A2780 on FN stably coexpressing Lifeact-iRFP670 (F-actin) and NB^GFP^-mCherry-(Ctrl) or fused with different R25 mutants and GFP-Kif5b[1-807] sorted as shown in fig. S6C (dox for 48 hours, 500 ng/ml; MIPs). FN-coated 96-well plate (Cellvis, #1.5H cover glass). Quantified in (**H**) is the number (*n*) of narrow protrusions (width ≤5 μm) per cell [yellow arrows in (G), example]. ANOVA on ranks with Dunn’s test (compared to the variant without Kif5b); *n* > 50 cells (*N* = 3). *P < 0.05; **P < 0.01; ***P < 0.001; ****P < 0.001. For the quantification of cell length, see fig. S6E. All scale bars, 10 μm.

To determine whether Rab25 has the capability to directly promote protrusions in its guanosine 5′-triphosphate (GTP) form and whether it requires localization on endosomes, we generated mutants of Rab25 that disrupted membrane anchorage (dC; fig. S5A) and/or mutated the GTP binding site [T26N; dominant negative (DN)]. These NB^GFP^-mCherry–fused Rab25 mutants [wild type (wt), dC, DN, or DN/dC] were introduced into A2780 cells (endogenous Rab25 expression nondetectable) using lentiviruses and sorted for comparable expression of mCherry. In comparison to the wt Rab25 protein, Rab25 dC, DN, and DN/dC mutants were more diffusely localized, suggesting a defect in the membrane recruitment and trafficking function for all these mutants (Fig. 2A). This observation is analogous to that previously demonstrated for Rab11b (*38*). GFP-MNPs were then microinjected to compare our ability to spatiotemporally control Rab25 variants using magnets. We found that Rab25 dC has fast and reversible relocalization kinetics, similar to those of the NB^GFP^-mCherry control (attraction time *t*_1/2_ ~ 20 s; relaxation time *t*_1/2_ ~ 10 s; Fig. 2, B and C). In contrast, membrane-bound Rab25 wt yielded typical values of *t*_1/2_ ~ 352 s with low reversibility (Fig. 2, B and C), consistent with previous optogenetic experiments (*29*). The quantification of the difference in relocalization kinetics between Rab25 dC and Rab25 wt provides further confirmation that it is possible to magnetically relocalize Rab25 endosomes rather than just membrane-free Rab25. In addition, the magnetic redirection and enrichment of Rab25 wt and Rab25 dC, but not inactive Rab25 dC/DN, to the cell periphery promoted F-actin protrusion growth (Fig. 2, D and E, fig. S5B, and movie S3). This suggests that the effect of Rab25 on protrusion, once localized, is direct and requires Rab25 activity, but at least on 2D substrates, vesicle association is not essential. A small proportion of Rab25 dC mutant is associated with stress fibers (Fig. 2A). This was particularly evident in ~25% of cells upon GFP-MNP delivery by microinjection and was not amenable to magnetic force, suggesting highly stable association (fig. S5C).

To compare our magnetogenetic results using an independent approach, we developed an additional strategy on the basis of our NB^GFP^ module and GFP-tagged molecular motor Kif5b, which has previously been shown to associate with Rab11 vesicles (*39*). GFP-fused Kif5b[1 to 807] was truncated to prevent endogenous cargo binding (*27*) and used to semidirectly manipulate Rab25 variants to the cell periphery via NB^GFP^:GFP interaction (Fig. 2F). Stable expression of Kif5b[1 to 807]-GFP enriched the NB^GFP^-mCherry control or fused variants of Rab25 in the cell periphery and, in combination with Rab25 wt and dC, but not control, induced an increase in cell length (fig. S6, A and B). Cells with Rab25 wt formed narrow protrusions where Rab25 wt codistributed with the base of narrow filopodium-like structures. In contrast, protrusions promoted by Rab25 dC were characterized by wider veils of F-actin at the leading edge (fig. S6B). These findings suggest that the pattern of Rab25 positioning at the cell periphery is sufficient to drive the extension of actin-rich membrane protrusions from the cell periphery. When the levels of Kif5b[1 to 807]-GFP were closely matched in GFP-mCherry fusion–expressing cells (fig. S6C), a significant reduction in the proliferation rate of Rab25 wt/Kif5b–coexpressing cells was observed (fig. S6D). This was accompanied by a notable increase in the number of narrow protrusions and the length of the cells coexpressing Rab25 wt/Kif5b (Fig. 2, G and H, and fig. S6, E and F). In contrast, the cells coexpressing Rab25 dC/Kif5b exhibited an increase in length but not in the number of protrusions (Fig. 2, G and H, and fig. S6, E and F). We next tested whether these differences are linked to the integrin β1 adhesion receptor cargos, previously shown to interact with Rab25 endosomes (*11*). The treatment of cells with β1-blocking antibodies resulted in decreased cell length across all cell lines examined (Ctrl, Rab25 wt, and Rab25 dC), irrespective of the presence or absence of coexpression of Kif5b[1 to 807]-GFP (fig. S6, E and F). Rab25 dC/Kif5b– and Ctrl/Kif5b–expressing cells showed a more rounded morphology with lamellipodium-like ruffles upon β1 blockade (fig. S6, F and G). However, Rab25 wt/Kif5b cells maintained their characteristic morphology with multiple narrow protrusions (fig. S6, F to H). Rab25wt/Kif5b protrusions were more short-lived in the presence of β1-blocking antibodies (fig. S6I), suggesting that Rab25 wt cells specifically require integrin β1 to form long, narrow, and stable protrusions.

Together, these data demonstrate that the positioning of Rab25 at the cell periphery is sufficient to drive the extension of actin-rich membrane protrusions from the cell periphery. The morphology of these protrusions is a dictated association of Rab25 with vesicles, which is likely to restrict the delivery of cargos/signals. We further suggest that magnetogenetics is superior to methods that rely on recruiting engineered cytoskeletal motors, which suffer from overexpression side effects and are limited by the cellular distribution of the engineered molecular motors and their polarized movement.

### Manipulation of Rab25 recycling endosomes in the 3D matrix triggers robust F-actin protrusion

**Fig. 3.**
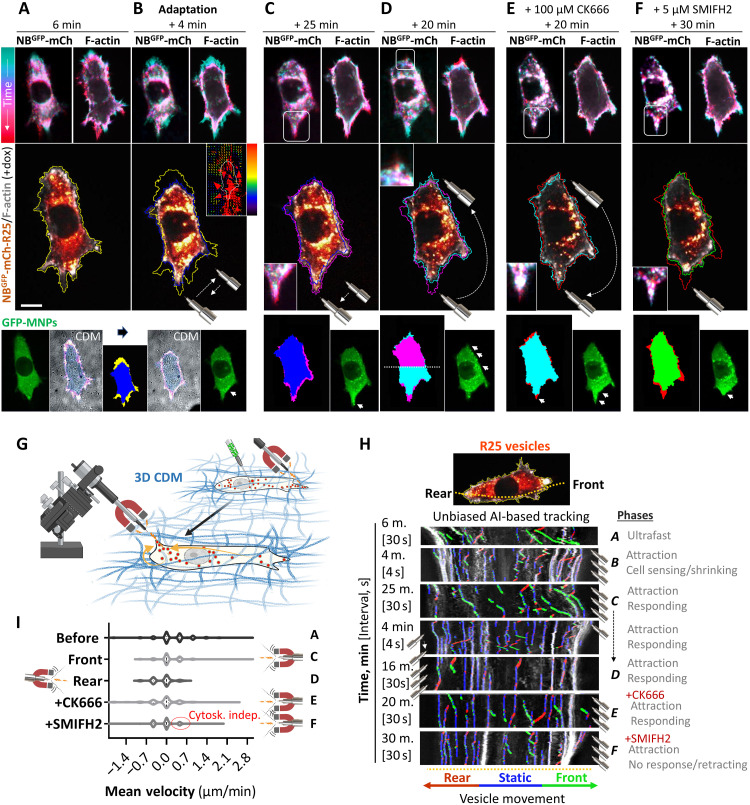
Manipulation of R25 recycling endosomes in living cells migrating in a 3D matrix triggers robust F-actin protrusion. (**A** to **H**) Representative images from confocal spinning-disk live-cell imaging. A2780 DExCon-modified NB^GFP^-mCherry-R25 cells (see fig. S1) pretreated with dox (72 hours; 250 ng/ml) expressing Lifeact-iRFP670 (F-actin) migrating in 3D CDMs. GFP-MNPs delivered by microinjection and repeatedly relocalized using a magnetic tip (movement specified) as depicted in (G) (created in BioRender, https://biorender.com/u40q760) in the presence or absence of different indicated inhibitors. See movie S4. *N* = 3. [(A) to (F)] Masks created from F-actin images (solid colors overlaid from preceding and following events). Arrowheads, gradient of GFP-MNPs. Color-grade (cyan-red) LUT images show changes in vesicles (NB^GFP^-mCh-R25)/GFP-MNP distribution and cell shape (F-actin) in time. The relative stress map [inset in (B)] is displayed as a vectorial plot with red-blue LUT; the size and direction of vectorial arrows illustrate force distribution exerted on CDMs. Scale bar, 20 μm. [(H) and (I)] AI-based tracking of vesicle (NB^GFP^-mCh-R25) movement (labeled blue, static; red, toward the rear; green, toward the front) using kymographs (generated from the yellow dashed line) and Kymobutler plug-in during experiment shown in (A) to (F). Note that the ultrafast phase kymograph (A) of vesicle movement was corrected by eye. The position of the magnet relative to the cell front and rear is shown. The total quantification of (H) is shown in (**I**).

Next, we aimed to replicate our results under more challenging but physiological conditions using cell-derived matrices (CDMs) (*40*). In this 3D environment, A2780 cells exhibit increased migration and front-rear polarization (Fig. 3A) (*41*) and form invasive protrusions (*11*, *19*, *42*). A gradient of mCherry protein was magnetically controlled through the interaction of NB^GFP^:GFP-MNPs without any noticeable impact on actin dynamics in control cells (fig. S7A). Upon expression of DExCon-modified NB^GFP^-mCherry-Rab25, the proximity of the magnetic tip to GFP-MNP–microinjected cells resulted in an unanticipated temporary cell contraction adaptation (Fig. 3, A and B, and movie S4). This resulted in a pulling force being exerted on surrounding matrix fibers (Fig. 3, A and B, and fig. S7B). The cell adaptation was reproducibly noticeable when the magnet was moved into the field of view but only for NB^GFP^-mCherry-Rab25–expressing cells. When cells were treated for 30 min with blebbistatin, no adaptation and matrix perturbation were observed (fig. S7B). This suggests that the actomyosin cytoskeleton is involved in the mechanosensing of magnetic force by MNP-bound Rab25 endosomes.

Cells adapted to the remote mechanical perturbation within ~5 min. Subsequently, MNP-bound Rab25 endosomes were attracted toward the magnet, and near-simultaneously, protrusion growth was observed within a region of the cell that had been protruding (dark blue to magenta outlines; Fig. 3C). Notably, the repositioning of the magnet toward the cell rear led to Rab25 vesicle accumulation and actin protrusion formation in the posterior part of the cell, which previously lacked protrusive structures (magenta to cyan outlines; Fig. 3, D and G, and movie S4). The repositioning of the magnetic tip to its original position promoted vesicle movement accompanied by actin-based protrusion in a previously retracting region of the cell, even in the presence of the Arp2/3 inhibitor CK666 (cyan to red outlines; Fig. 3E). In contrast, addition of a formin inhibitor (SMIFH2) caused the retraction of the protrusion, suggesting a dependence on the formin family of actin-polymerizing proteins (red to green outlines; Fig. 3F and movie S4). The unbiased AI-based vesicular tracking confirmed that local actin polymerization near the cell periphery (Fig. 3, A to F; top right F-actin color-grade time lapses) follows the relocalization of Rab25 endosomes that are controlled by magnetic tip movement (Fig. 3, H and I). SMIFH2 treatment disrupted this connection between Rab25 and F-actin protrusion outgrowth (Fig. 3, H and I). We then similarly tested the exogenously expressed Rab25 dC mutant with impaired membrane recruitment. However, despite rapid Rab25 dC attraction via magnetic repositioning of GFP-MNPs, we were unable to induce protrusions/PM growth in any of four biological repeats (fig. S7C).

We further confirmed our observation by an indirect approach, scoring the extension of pseudopodial protrusions at the cell front, previously associated with Rab25 expression in invasive cells (*11*). The reactivation of endogenous Rab25 or expression of exogenous Rab25 wt correlated with an extension in pseudopodial protrusions in front of invasive cells and an increase in cell migration persistence. No change in average speed was observed (fig. S8, A to E), which is consistent with previous findings (*11*, *21*). Nevertheless, the expression of DN Rab25 or Rab25 uncoupled from endosomes (Rab25 dC) did not result in an increase in pseudopod length or cell migration persistence (fig. S8, A to E). To enhance the redistribution of Rab25 mutants to the cell periphery and to partially rescue the diffuse localization of the Rab25 dC variant, we again stably coexpressed Rab25 variants fused to NB^GFP^-mCherry and Kif5b[1 to 807]-GFP. For Rab25 wt, Kif5b expression resulted in a notable increase in pseudopod extension at the cell front to a significantly greater extent than Rab25 wt alone (fig. S8, A to C). This effect was accompanied by high migration persistence (fig. S8, D and E). Notably, this phenomenon was not observed in the control or in the Rab25 dC mutant, in contrast to the effects observed on cell elongation on 2D substrates. Formin inhibition (SMIFH2) blocked the ability of Kif5b-Rab25 wt to induce pseudopod extension in a dose-dependent manner (fig. S8F), consistent with previous results.

The inability of the Rab25 dC mutant to promote membrane/protrusion growth under 3D conditions, despite peripheral enrichment by kinesin motors or magnetogenetic targeting to the cell periphery, suggests that the spatiotemporal control of Rab25 is not sufficient to control all aspects of protrusion formation. Rab25-driven tumor cell invasion into fibronectin (FN)-rich CDMs is highly dependent on Rab25’s ability to interact with integrin β1–containing recycling vesicles (*11*). The elongation of pseudopodial protrusions promoted by Kif5b-dependent relocalization of Rab25 endosomes could be inhibited by integrin β1–blocking antibodies, suggesting that in addition to formins, integrin β1 is also an important factor for protrusion formation (fig. S8F). Local targeting of GFP-MNPs:Rab25 endosomes to the cell periphery of cells in 3D CDMs reproducibly induced protrusion growth, but this was completely abrogated by integrin β1 blockade (fig. S8G and movie S5). These data suggest that the delivery of integrin β1–containing vesicles to specific membrane sites is critical for Rab25-mediated protrusions in 3D CDMs. Together, these results demonstrate the critical role of Rab25 endosomes as a platform for the spatiotemporal control of Rab25 activity, coordinating modulation of actin polymerization with integrin adhesion receptor delivery for protrusion formation in the 3D matrix.

### Rab25 recycling endosomes recruit FMNL1

To identify Rab25 proximal interacting actin nucleators in living A2780 cells, we reanalyzed our previously published Rab25-BioID (proximity labeling) dataset (*22*). In addition to known Rab25-associated membrane receptors [such as endothelial growth factor receptor (EGFR) and integrin β1], motors (such as Kif5b), and motor-coupling proteins (such as RAB11FIP2, which binds to MyoVb), we noted that one of the most highly enriched hits was the actin nucleator FMNL1, which belongs to the formin family (fig. S9A).

**Fig. 4.**
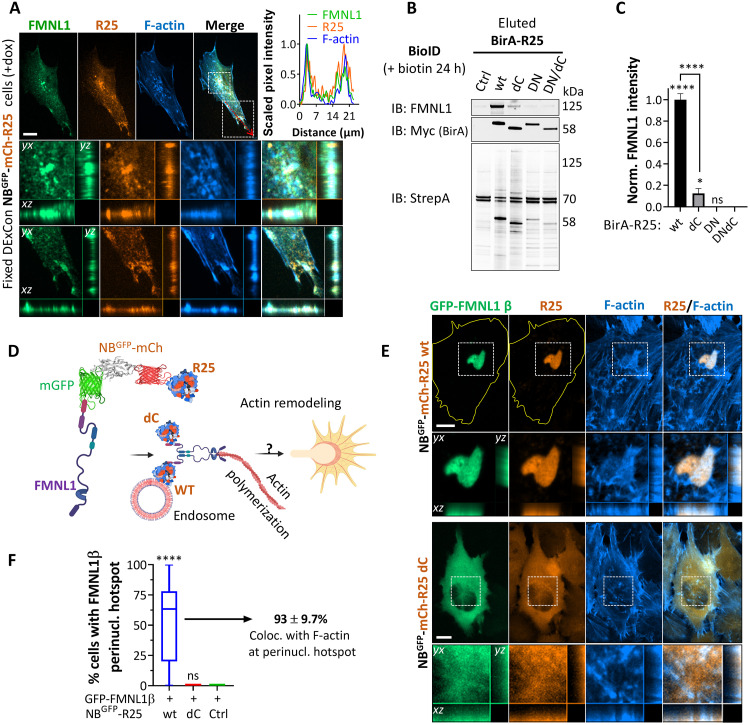
R25 recycling endosomes recruit FMNL1. (**A**) Representative confocal spinning-disk images of A2780 DExCon-modified NB^GFP^-mCherry-R25 cells (mCherry, orange/amber) pretreated with dox (>94 hours; 250 ng/ml) immunolabeled for FMNL1 (green, anti-FMNL1, Alexa Fluor 488) and stained for F-actin (blue, phalloidin-Alexa Fluor 633). Red dotted line, line scan profile of normalized 0- to 1-scaled fluorescence intensities. White box, zoom inset (MIP) with cross sections. FN-coated μ-Plate 96 plate (#1.5 IbiTreat). Scale bar, 10 μm. (**B** and **C**) BioID experiment. A2780 stably expressing BirA fused R25 wt or mutants (dC, DN, and DN/dC) cultured with biotin (1 μM biotin, 16 hours). Lysates equalized to the total protein amount and biotinylated proteins pulled down with streptavidin beads (Ctrl, no BirA). (B) Immunoblots (IBs) of pulled-down FMNL1, BirA (Myc epitope), and streptavidin (StrepA) as the loading control. Fluorescent antibodies are shown in black and white. (C) Quantification of pulled-down FMNL1 from immunoblots. The graph shows band intensities normalized to individual BirA levels and relative to BirA-R25 wt values (background from Ctrl sample subtracted). Means ± SD; *N* = 3. One-way ANOVA analysis with Tukey post hoc test (compared to DN or dC or as indicated); *P < 0.05; ****P < 0.001. (**D**) Schematic diagram of the strategy to enforce the proximity of FMNL1β with R25 wt or dC mutant unable to bind endosomes by the interaction of GFP(-FMNL1) and NB^GFP^(-mCherry-R25). Created with BioRender.com (https://biorender.com/f9jfcq2). (**E**) Representative confocal spinning-disk live-cell images of A2780 stably coexpressing Lifeact-iRFP670 (F-actin) and GFP-FMNL1β with NB^GFP^-mCherry-(Ctrl) or fused with different R25 mutants. The cell shape is outlined in yellow. The zoom inset from the white box is shown as MIP with cross sections. FN-coated 96-well plate (Cellvis, #1.5H cover glass). Scale bar, 10 μm. Quantified in (**F**) as % of cells with the FMNL1 β perinuclear hotspot as shown in (E). The box plot shows the median and 25th and 75th percentiles with whiskers reaching the last data point; *n* > 55 cells per condition; *N* = 3. One-way ANOVA analysis with Tukey post hoc test (compared to Ctrl); ****P < 0.001.

The use of confocal spinning-disk microscopy revealed the colocalization of Rab25 and FMNL1 on perinuclear recycling endosomes, which were surrounded by abundant F-actin (Fig. 4A). In addition, we found high colocalization of Rab25, FMNL1, and F-actin in actin-rich protrusions, including filopodium-like structures (fig. S9B). FMNL1:Rab25 remained highly colocalized upon inhibition of cytoskeletal elements/regulators, albeit with a slight decrease upon nocodazole treatment (fig. S9, C and D). While pan-formin inhibition did not disrupt FMNL1:Rab25 association, it did decrease the association of both with F-actin. Notably, treatment with nocodazole or low-dose cytochalasin D disrupted the perinuclear Rab25 enrichment and resulted in a redistribution of FMNL1 and F-actin toward the cell periphery (fig. S9, C and D).

To characterize FMNL1:Rab25 interaction in live cells, we used a knock-sideways relocalization approach, whereby Rab25 fused to FKBP is relocalized (*t*_1/2_ ~ 5.4 ± 0.1 min) (*22*) to the outer mitochondrial membrane upon rapamycin treatment via interaction of FKBP:FRBmito (fig. S9E). GFP-FKBP-Rab25 or GFP-FKBP was highly enriched to mitochondria upon rapamycin treatment, and there was a subtle redistribution of FMNL1 with GFP-FKBP-Rab25, suggesting that the Rab25:FMNL1 interaction is weak and transient or that redistribution is limited by other factors (fig. S9, F and G).

To determine whether Rab25’s association with FMNL1 is restricted to endosomes and/or to active Rab25, we conducted additional BioID experiments using Rab25 wt and Rab25 mutants (dC, DN, and DN/dC). While Rab25 wt induced extensive proximal biotinylation of FMNL1, inactive DN forms of Rab25 did not (Fig. 4, B and C). However, Rab25 dC was also able to do so, albeit to a far lesser extent (Fig. 4, B and C). This suggests that endosomal localization is beneficial for Rab25 association with FMNL1 but not essential. The enrichment of Rab25 wt or Rab25 dC at the cell periphery by the Kif5b[1 to 807]-GFP motor, via NB^GFP^:GFP interaction, showed strong colocalization with FMNL1 for both variants (fig. S9H). This suggests that Rab25 dC encounters FMNL1 at the PM rather than endosomes, hence lower association. Integrin β1 proximity labeling, however, was restricted solely to Rab25 wt, consistent with our previous findings that Rab25-GTP interacts with integrin β1 (fig. S9I). This indicates that endosomal localization is required for Rab25 to interact with integrin β1.

Three isoforms of FMNL1 (α, β, and γ) have been described, differing in the regulatory sequence at the C terminus. Of these, FMNL1γ appears to be constitutively active (*43*, *44*). We identified the regulatable FMNL1β isoform as the major isoform in A2780 cells (fig. S10A). To provide evidence of FMNL1 activity and the functionality of the FMNL1β/Rab25 association in A2780 DExCon Rab25 cells, we enforced the proximity of FMNL1β and Rab25 by the interaction of GFP(-FMNL1) (Fig. 4D). This resulted in stable endosomal colocalization of Rab25, FMNL1, and F-actin (fig. S10B). To support our hypothesis that endosomes locally regulate Rab25 activity to spatiotemporally control FMNL1-dependent actin nucleation, we generated stable cell lines coexpressing GFP-FMNL1β/NB^GFP^-mCherry-Rab25 wt or GFP-FMNL1β/NB^GFP^-mCherry-Rab25 dC. The sustained association of FMNL1β with NB^GFP^-mCherry-Rab25, but not with diffuse Rab25 dC, resulted in notable coenrichment of FMNL1β and Rab25 wt collapsed into a cluster in the perinuclear area in ~50% of cells, with prominent actin nucleation at these sites in most cells (Fig. 4, E and F). Overall, these results demonstrate that Rab25 recycling endosomes locally can change actin polymerization via FMNL1 and that this association is likely to be transient in normal cells to maintain the F-actin network.

### Rab25 recycling endosomes modulate F-actin polymerization in protrusions via FMNL1

**Fig. 5.**
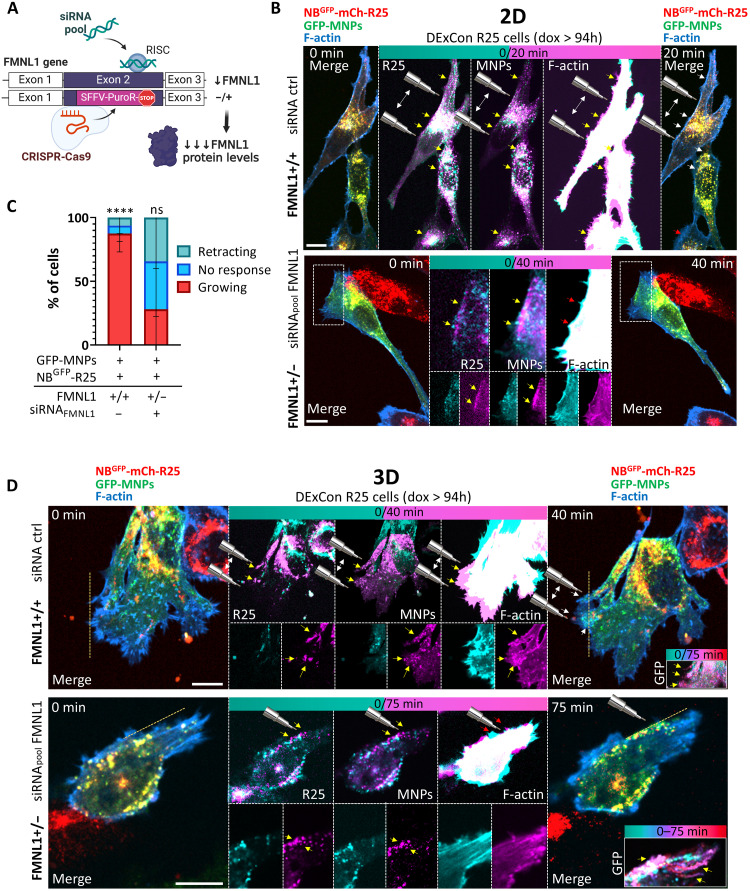
Magnetic spatiotemporal control of R25 endosomes modulates F-actin polymerization in protrusions via FMNL1. (**A**) Strategy to efficiently deplete FMNL1 using a smart pool of siRNAs in an FMNL+/− heterozygous background previously created by the CRISPR-Cas9 editing (see also fig. S10, C and D; for details, see Methods). Created with BioRender.com (https://biorender.com/nsezjwg). (**B** and **D**) Representative frames from confocal spinning-disk live-cell time-lapse images of A2780 DExCon-modified NB^GFP^-mCherry-R25 FMNL1+/+ or FMNL1+/− cells (dox treated for >94 hours; 250 ng/ml) stably expressing Lifeact-iRFP670 (F-actin) on FN, microinjected with GFP-MNPs and nucleofected with chemically modified siRNA_pool_ anti-FMNL1 or nontargeting Ctrl siRNA (see Methods) as indicated. Experiments were performed 5 days after (for immunoblots, see fig. S10D). R25 endosomes attracted using a magnetic tip (shown by cartoon; arrows indicate movement). Red arrows (time 0) or yellow/white arrows (later time point) indicate the outcome. MIPs if not stated otherwise. Zoomed insets correspond to areas indicated by boxed areas. Scale bar, 10 μm. Quantification in (**C**) as in Fig. 1 (C and D) as % of cells showing changes in protrusion growth toward the magnetic tip; *n* = 12 (siRNA Ctrl; *N* = 3) and *n* = 14 (siRNA_pool_ anti-FMNL1; *N* = 3; see movie S6), means ± SD. Ordinary one-way ANOVA (GraphPad); ****P < 0.001. (D) Representative images from confocal spinning-disk live imaging of cells in 3D CDMs; *N* = 3. MIP (top) or one-matched (0/40 min) *Z* plane is shown (bottom). Dashed line, cell edge (F-actin) at time 0. The color-grade (cyan-red) LUT image shows changes in GFP-MNP distribution in time. See movies S7 and S8.

The rearrangement of the actin cytoskeleton to promote actin-based protrusions requires nucleation and elongation of actin filaments, a process that is catalyzed by actin assembly factors (*45*). We inactivated the FMNL1 gene using CRISPR-Cas9–based gene editing in DExCon-modified NB^GFP^-mCherry-Rab25 cells. However, we were only able to generate heterozygote knockouts of one allele (for details, see Methods). Therefore, we developed a combination strategy that efficiently depletes FMNL1 using a smart pool of small interfering RNAs (siRNAs) in the FMNL−/+ heterozygous background previously created by CRISPR-Cas9 editing (Fig. 5A and fig. S10, C and D). To demonstrate that Rab25 promotes protrusions via FMNL1 we then directly re-localized NB^GFP^-mCherry-Rab25 in cells using magnets. In 2D, we again observed formation of small but robust and clearly discernible actin-rich protrusions in the direction of the magnetic field in most microinjected cells transfected with control siRNA, irrespective of their polarity [Fig. 5, B (top) and C]. By contrast, magnetic relocalization of NB^GFP^-mCherry-Rab25 toward the cell periphery did not induce protrusions in FMNL1-depleted cells [Fig. 5, B (bottom) and C, and movie S6].

In cells migrating in 3D, robust generation of protrusions was reproducibly induced by magnetic relocalization of NB^GFP^-mCherry-Rab25– and FMNL1-expressing cells [Fig. 5D (top) and movie S7]. However, this phenomenon was not observed following FMNL1 depletion, demonstrating that Rab25 endosomes directly promote actin-rich protrusions via FMNL1 [Fig. 5D (bottom) and movie S8]. Similarly, only in FMNL1-expressing cells, we were occasionally able to directly manipulate endosomes in a slowly moving cluster that induced the formation of a visible actin polymerization hotspot. This actin hotspot moved and highly colocalized with Rab25 and GFP-MNPs over 60 min, coattracted to the magnet, and returned after its removal (fig. S11), suggesting that FMNL1 regulates the formation of both actin tracks and nascent protrusions.

In summary, these results confirm our hypothesis that Rab25 endosomes promote F-actin polymerization and protrusions by recruiting the formin FMNL1 in addition to integrin β1. Furthermore, these proof-of-principle data demonstrate the power of our approach using magnets in combination with genetic perturbation to demonstrate the causal effect of Rab25 endosome positioning on F-actin protrusions in invasive cancer cells.

### Rab25 recycling endosomes serve as a signaling platform for RhoA/FMNL1 activity

**Fig. 6.**
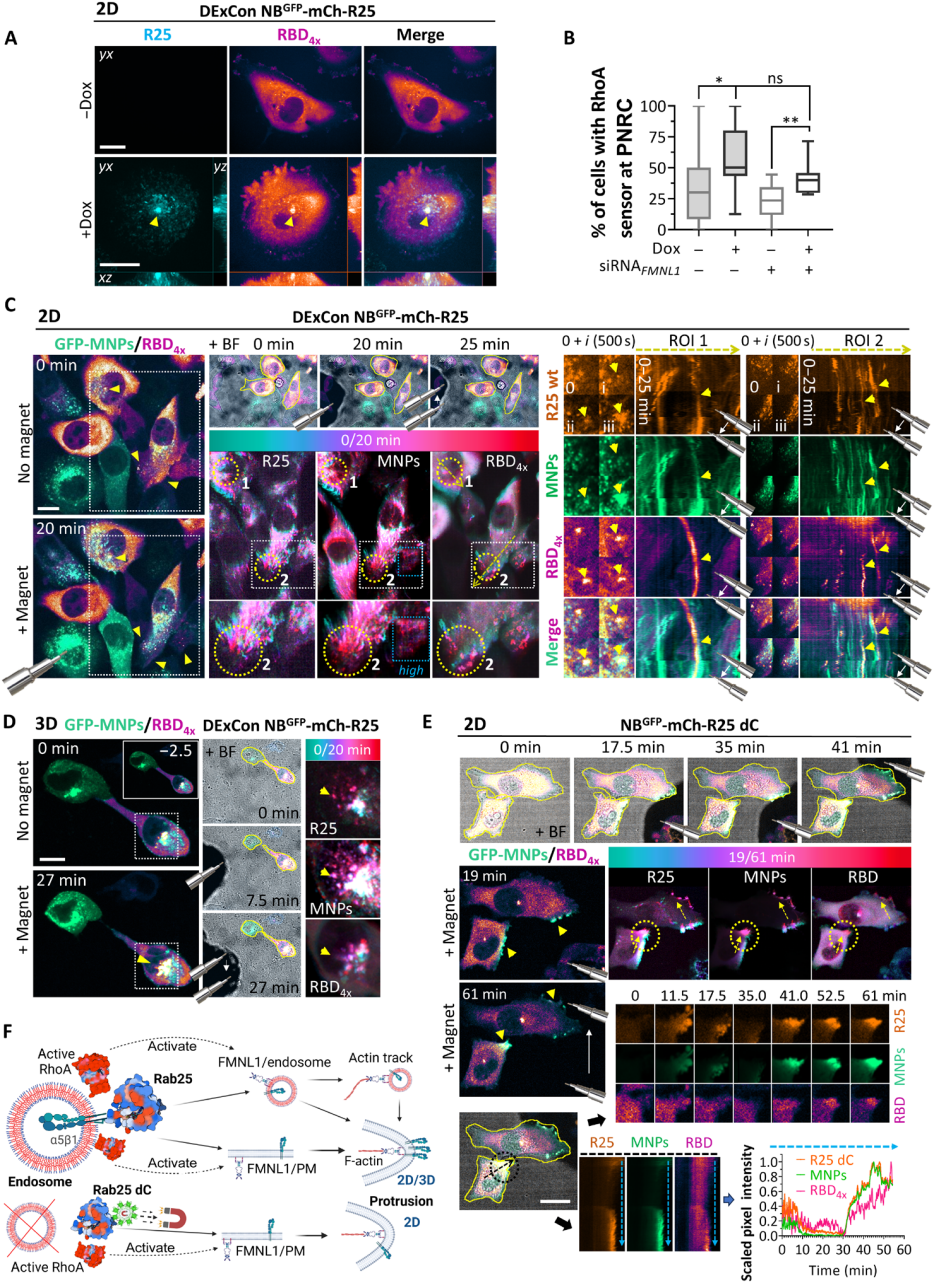
R25 recycling endosomes serve as signaling platforms for RhoA/FMNL1 activity. (**A**, **C**, and **D**) Representative spinning-disk live-cell images of A2780 DExCon-modified NB^GFP^-mCherry-R25 (dox for >72 hours; 250 ng/ml) or (**E**) A2780 NB^GFP^-mCherry-R25 dC stably coexpressing active RhoA probe iRFP670_3x_-RBD_4x_ (RBD_4x_). GFP-MNPs microinjected (green). Color-grade (cyan-red) LUT image, changes in mCherry/GFP-MNPs/RBD_4x_ in time (generated from the one-matched *Z* plane). Yellow arrowheads/arrows, mCherry/GFP-MNPs/RBD_4x_ signal enrichment/changes/movement. Magnetic tip position, shadow/cartoon (white arrows, movement). Cell shape, yellow line outline in merged/bright-field images. Scale bar, 20 μm. (A) MIPs with cross sections and cells on FN. Arrow, PNRC. Quantified in (**B**) as the percentage of cells with the fluorescent signal of RBD_4x_ visibly enriched at the PNRC spot ± R25 (±dox for 48 h; *n* > 60 cells, *N* = 3) ± siRNA_pool_ anti-FMNL1 (*n* > 40 cells, *N* = 2). One-way ANOVA with Tukey’s multiple comparison test; *P < 0.05; **P < 0.01. (C) Cells on FN; one-matched *Z* plane for all. See movie S9. White boxed areas, ×3 color-grade LUT images for individual channels with indicated zoomed insets (bottom); blue box, higher contrast. Circle 1, zoomed inset of ROI1 (time interval, 500 s = *i*). Circle 2 with a yellow dotted line, zoomed inset of ROI2 as the kymograph. (D) 3D CDM. GFP-MNPs/RBD_4x_ merge, MIPs. Top inset: −2.5 min; no magnet. Movie S22 is accessible via https://doi.org/10.6084/m9.figshare.22155083. (E) FN-coated coverslip. One-matched *Z* plane for all. See movie S10. Yellow circle, zoomed inset of time lapse for individual channels. Black circle and black dashed line, zoomed inset as the kymograph for individual channels. Blue dashed line across the kymograph, line scan profile of normalized 0- to 1-scaled fluorescence intensities. (**F**) Schematic model summarizing our results: R25 endosomes serve as a platform for the spatiotemporal control of R25 activity (possible to directly modulate using magnetogenetics). This locally modulates RhoA activity to promote actin polymerization in both actin tracks and nascent protrusions through the FMNL1-dependent actin nucleation mechanism while simultaneously delivering integrin receptors for protrusion formation/stabilization. Created with BioRender.com (https://biorender.com/1pdoxf0).

Endosomes, including Rab11 recycling vesicles, are emerging platforms for integrin-mediated signaling that are linked with local RhoA activity to fine-tune actomyosin-based contractility, thereby establishing front-rear cell polarity (*20*, *46*–*49*). FMNL1 has been recently identified as a proximity interactor of RhoA (and of RhoB, RhoG, RhoD, RhoU, RhoF, and RhoJ), binding to its active form (*50*, *51*). Because we identified a major FMNL1β isoform in A2780 cells, whose activity is likely regulated by Rho GTPases, we tested whether the activity of RhoA might be locally modulated by the magnetic relocalization of Rab25-positive endosomes. To monitor endogenous RhoA activity, we adapted a localization-based activity probe iRFP670_3x_-RBD_4x_ that detects active RhoA with improved specificity and sensitivity (*52*). We found that the active Rho probe iRFP670_3x_-RBD_4x_ is enriched with Rab25 at the perinuclear recycling compartment (PNRC) in a Rab25-dependent manner, both in 2D and 3D (Fig. 6, A and B, and fig. S12, A and B), and in vesicles moving at the cell periphery (fig. S12C). This localization was not perturbed with acute FMNL1 depletion (Fig. 6B). Furthermore, GFP-RhoA colocalized with mCherry-Rab25 at the base of narrow filopodium-like protrusions (fig. S12D), as well as with the Rho activity probe in the perinuclear region of cells (fig. S12E). This overlap was more pronounced upon expression of the constitutively active Q63L RhoA form but not the DN T19N RhoA mutant (fig. S12E).

Given the colocalization of Rab25 endosomes with RhoA and the active Rho probe (fig. S12C), we reasoned that magnetic relocalization of Rab25 toward the PM could lead to dynamic co-redistribution of RhoA activity as well. Magnetic redistribution of NB^GFP^-mCherry-Rab25 correlated with local RhoA activity changes in cells moving under 2D and 3D (CDMs) conditions (Fig. 6, C and D, and movie S9). Sustained attraction of Rab25 endosomes resulted in an increased localization of the active Rho probe in a punctate pattern in cell protrusions that were in close proximity to, but not completely superimposed with, Rab25 vesicles and GFP-MNPs (Fig. 6, C and D, and movie S9).

Previous studies have suggested that α5β1 recycling can modulate RhoA activity through coordinated polarized signaling of receptor tyrosine kinases, including EGFR1 and c-Met, as well as the activity of the RacGAP1-IQGAP1 complex (*20*, *53*, *54*). We therefore tested the ability of the Rab25 dC variant, which cannot associate with vesicles containing integrin β1 but can promote cell protrusion in 2D, to locally modulate RhoA activity. Magnetic spatiotemporal control of an NB^GFP^-mCherry-Rab25 dC gradient resulted in a rapid local increase in the intensity of the active Rho probe, which followed Rab25 dC enrichment toward the cell periphery or across the cell after redirection by the magnet (Fig. 6E and movie S10). However, a punctate pattern of the active Rho probe was not observed before or after relocalization in protrusions of Rab25 dC–expressing cells, suggesting that Rab25 can directly modulate RhoA activity, regardless of endosome association.

Together, our magnetogenetic approach demonstrates the importance of Rab25 endosomes as a platform for the spatiotemporal control of Rab25-driven activities. This control locally modulates RhoA activity to promote actin polymerization in protrusions through an FMNL1-dependent actin nucleation mechanism while simultaneously delivering integrin adhesion receptors for protrusion formation and stabilization in physiologically relevant 3D matrices (Fig. 6F).

## DISCUSSION

The contribution of endocytic recycling to tumor progression and cancer cell invasion has been well documented. However, the understanding of the precise mechanisms through which recycling vesicles can generate or maintain protrusions upon arrival at the PM has been hampered by an inability to directly manipulate vesicle positioning in time and space. Here, we developed a magnetogenetic approach for manipulation of vesicle positioning by engineering the endogenous expression of the recycling regulator Rab25 such that it is expressed with an anti-GFP nanobody tag (NB^GFP^). This allows coupling of Rab25 vesicles to magnetic particles and their relocalization when placed within a magnetic field. We used this approach to demonstrate that the positioning of Rab25 vesicles to the PM is sufficient to generate nascent F-actin protrusions in cancer cells. These protrusions are dependent on the actin-polymerizing protein FMNL1, and Rab25 coordinates the activity of RhoA with the arrival of FMNL1 and integrin β1 cargos to form protrusions that promote invasive migration of cancer cells in the 3D matrix.

Optogenetic approaches have been developed to manipulate endosome positioning, including via Rab11 family proteins (*27*, *29*). Light-inducible dimerization was used to couple Rab11 to motor protein kinesin or dynein, which was able to promote or perturb the dynamics of neuronal growth cones, respectively, after illumination (*27*). However, these approaches rely on the coupling between the target protein and a motor attached to a cytoskeletal element, and hence, relocalization is therefore limited by the polarization/orientation of the cytoskeleton. To overcome this limitation, we used a magnetogenetic technique, building on previous approaches (*30*–*32*, *55*–*57*). The nonspecific manipulation of endolysosomes (where endosomes and lysosomes are loaded through the fluid phase uptake of 991 ± 378 magnetic particles) was shown to bias cancer cell movement in 2D toward magnetic micropillars (reaching a force of 22 ± 16 pN per cell at a 10-μm distance) (*55*) and a small shift in directional neurite outgrowth when cultured in a magnetic field gradient (*58*). Similarly, magnetic particles functionalized with TrkB agonist antibodies, delivered nonspecifically into neurons by endocytosis, were able to stop neurite growth in response to a magnetic field (15-pN force) (*57*). For our purposes, microinjection of magnetic particles proved a suitable delivery method for loading of cells expressing NB^GFP^-mCherry proteins and enabled specific manipulation of both cytosolic proteins and Rab25 coupled to endosomal membranes (Figs. 1 and 2, B to E, and movies S2 and S3), even in cells migrating in the 3D matrix (Figs. 3, A to G, and 5D; fig. S7, A to C; and movies S4, S7, and S8). Magnetic manipulation of endosomal proteins was slower than that of cytosolic proteins (Fig. 2C), suggesting that endosomal proteins are coupled to endosomes and to the cytoskeleton and that magnetic redistribution may be enabled by transient detachment of endosomes from the cytoskeleton or associated motor proteins.

Rab11 has previously been shown to recruit F-actin regulators, which can generate tracks to permit vesicle trafficking over long distances within oocytes (*17*). Similarly, the trafficking regulator Rab27 has been demonstrated to cooperate with actin nucleators to drive long-range actin-dependent transport of melanosomes in melanocytes (*59*). Our data confirm that Rab25 may similarly promote the formation of F-actin tracks (figs. S1, H and I, and S11). Furthermore, Rab25 vesicle movement shows a modest but significant dependence on formins (fig. S4B) and Rab25 and FMNL1 show a tendency to move together with local effects on F-actin under different conditions (fig. S9, B to H). Forcing an interaction between NB^GFP^-mChRab25 Rab25 and GFP-FMNL1 leads to a change of F-actin morphology (fig. S10B), which can result in the collapse of peripheral Rab25 recycling endosomes to a large F-actin– and FMNL1-positive cluster (Fig. 4, E and F). These data suggest that the F-actin tracks involved in Rab25 vesicle positioning are also formin-dependent in this system, which could be mediated by FMNL1 recruitment. The fact that Rab25 vesicles are closely associated with the F-actin cytoskeleton is also reflected in the adaptation of cells to the introduction of magnetic force, which is only seen in a pliable 3D matrix, and the adaptation observed is dependent upon the contractile F-actin cytoskeleton (Fig. 3B and fig. S7B). This observation could also indicate that endosomal vesicles are involved in mechanosensing and cellular mechanoprotection (*60*) and is consistent with studies describing roles for Rab11, Rab25, and the F-actin network in the positive regulation and propagation of actomyosin contractile forces in response to developmental morphological change (*61*–*63*).

Our direct manipulation of Rab25 vesicle positioning demonstrates that upon reaching the cell periphery, Rab25 can initiate the formation of nascent F-actin–based protrusions in a formin-dependent, Arp2/3–independent manner. Rab25 associates with FMNL1 to a greater extent when it is able to stably interact with vesicles (Fig. 4, B and C), and Rab25 vesicles simultaneously coordinate the delivery of FMNL1 to the PM with the arrival of active Rho GTPases (Fig. 6, C to E). Previous studies demonstrate that the interaction of FMNL1 (and FMNL2) with Rho GTPases supports actin-based protrusions by properly orienting formin toward the membrane and forming an outward membrane bulge for growth of both lamellipodia and filopodia (*64*) and RhoA has also been implicated in filopodium and lamellipodium formation (*65*). The longer residence time of vesicular Rab25 at the periphery, as opposed to rapid diffusion of Rab25dC, may prolong the activation of FMNL1 and RhoA, resulting in filopodium-like protrusions through the actin-polymerizing activity of the formin and RhoA-mediated suppression of Rac signaling (which promotes lamellipodium formation).

On 2D substrates, while Rab25 wt repositioning can drive modest changes in cell protrusions, the changes associated with the repositioning of Rab25 dC are more notable. In 2D migration on a rigid flat surface, lamellipodia are particularly important for driving cell motility, but in 3D matrices, more plasticity in migration mode is observed and structures such as filopodia can promote prominent invasiveness (*42*). Our previous work has shown that the Rab11 family promotes invasiveness of cancer cells in the 3D matrix, while effects on migration in 2D are more subtle (*11*, *54*). The effectiveness of Rab25 dC in promoting protrusions on 2D substrates may be explained by faster relocalization kinetics (Fig. 2, B and C), and its modest association with FMNL1 is likely enhanced upon repositioning to the cell periphery where a pool of FMNL1 resides and colocalizes with Rab25 dC (fig. S9H). Notably, relocalization of Rab25 to the cell periphery induced robust formation of F-actin–rich narrow protrusions tipped predominantly by filopodium-like structures in 3D matrices, but uncoupling of Rab25 from endosomes abrogated this effect (figs. S6A and S8, A and B). We also found that integrin β1 is able to associate with vesicular Rab25 but is not detectable when Rab25 is truncated to inhibit vesicle recruitment (fig. S9I). Invasion and protrusion elongation driven by Rab25 are dependent upon integrin β1 (fig. S8, F and G) (*11*), and using this innovative magnetogenetic approach, we now demonstrate that the ability of Rab25 vesicles to drive the formation of F-actin–rich protrusions in 3D matrices is a product of the ability to coordinate the activity of Rho GTPases together with the delivery of FMNL1 and integrin β1.

We found that a truncated form of Rab25, in which the C-terminal prenylation sites are deleted, is able to facilitate protrusion despite the lack of stable vesicle interaction (Fig. 2, D and E, and movie S3). While it is possible that truncated Rab25 can interact with effectors and thus associate with vesicles to some extent, the limited association of Rab25 dC with FMNL1 (Fig. 4, B and C) and the lack of detectable association with integrin β1 (fig. S9C) suggest that this is minimal. Instead, we suggest that Rab25 dC encounters FMNL1 at the PM to support the initiation of protrusions, perhaps because Rab25 dC is still able to support the activation of Rho GTPases that are required for FMNL1 activity (Fig. 6E and movie S10). This leads us to suggest that Rab25 plays a dual role in the regulation of F-actin by modulating Rho GTPases and associating with actin-polymerizing proteins. However, while these activities alone are sufficient to initiate cell protrusion, the formation of more stable protrusions requires the vesicular association of Rab25 to link to other cargos, including integrins (Fig. 6F).

### Limitations and future directions

We show that repositioning of Rab25-positive recycling endosomes promotes protrusion, where Rab25 appears to be enriched at a defined subdomain of the outer vesicular membrane (Fig. 1F). However, we cannot exclude the possibility that in contrast to Rab25 dC, magnetically manipulated Rab25-positive endosomes also contain other Rab GTPases. Rab11/Rab4/Rab5 can be present on the same endosomes in the recycling pathway in different membrane domains at different ratios (*66*). It will be interesting to determine the influence of other Rab GTPases (e.g., Rab11a/b in recycling endosomes; Rab5 or Rab4 in early endosomes; Rab7 in late endosomes) on cell protrusion in the future, as many cargos can be shared. Our data indicate that Rab25 may promote filopodium-like protrusion formation, consistent with our previous observations (*22*). It is therefore possible that distinct sets of cargos driven by specific trafficking routes will influence cell morphology in different ways. Similarly, we have identified FMNL1 and integrin β1 as key cargos that mediate protrusion. Given the broad range of adhesion receptors, signaling receptors, and protein cargos carried by different vesicular pathways, identifying how these contribute to cell protrusion and motility is a priority. Our data also suggest that the vesicular association is important to regulate the function of Rab25 beyond the delivery of conventional cargos however. Targeting of vesicles provides a mechanism to spatially restrict a range of cargos, and vesicular Rab25 plays a role in regulating the spatiotemporal activity of RhoA via a mechanism yet to be determined. We therefore suggest that Rab25 regulates vesicle trafficking, and vesicle association focuses on Rab25-dependent cargos and signaling events to promote cell protrusion for invasion and metastasis.

## METHODS

Detailed lists of antibodies, chemicals, sequences, plasmids, and resources used are described in tables S1 to S5 or accessible from https://doi.org/10.6084/m9.figshare.27084217.

### Constructs

A number of plasmid DNA constructs were used and generated in this study, and these are listed with details in table S1. Plasmid maps are provided here: https://doi.org/10.6084/m9.figshare.27084205. The online tool Benchling was used to design guide RNA sequences for specific CRISPR-Cas9 cleavage (table S2) and primers for cloning using Gibson assembly (www.benchling.com).

Plasmid constructs and donors for single-stranded DNA (ssDNA) synthesis (pJET-24_Rab25 DExCon, pJET-78-FMNL1-HR-SFFV-BlaR,and pJET-77-FMNL1-HR-SFFV-PuroR) were prepared as described previously using Gibson assembly or restriction endonuclease–based cloning (see final plasmid maps) (*21*). New sequences (gene specific exon/intron sequences for knockin or cDNA for overexpression) were synthesized commercially by GENEWIZ (FragmentGENE) or IDT (gBlocks) with 25 to 50–base pairs–long homologous overhangs for Gibson assembly depending on the synthesis limitations (GC-rich/repetitive sequences; sequences provided in table S2) and cloned into pJET1.2 (CloneJET PCR Cloning Kit, no. K1231; Thermo Fisher Scientific) or different vectors according to the requirements of the experiment (pCDH, pEGFP, pSBtet, …). Universal linkers (including Spe I/Sal I, Xba I/Xho I, Age I, Nhe I, Ecor I or other cleavage sites) were also included and used to add, switch, or remove additional sequences (see final plasmid maps). Lentiviral pCDH-NB^GFP^-mCherry-Rab25 was cloned using pCDH-NB^GFP^-mCherry-Rab11a (as an NB^GFP^-mCherry template) (*21*) and Xba I/Xho I–cleaved pCDH-tagBFP-T2A-mycBirA-Rab25 (*22*). To create the Rab25 dC mutant, Rab25 wt was cut with Bsp EI (=Kpn2I) and Sal I to remove the C terminus, filled with PfuX7, and religated (pCDH-tagBFP-T2A-mycBirA-Rab25 dC) or generated with specific Gibson assembly primers (pCDH-tagBFP-T2A-mycBirA-Rab25 dC) for Rab25 dN (T26N) with the mutagenesis site. pLenti Lifeact-iRFP670 BlastR was purchased from Addgene (no. 179888) (*67*). The optimized Sleeping Beauty transposon–based system pSBtet-puroR-BFP (Addgene Plasmid no. 60496) (*68*) was opened by SfiI cleavage, and pB72_Kif5b-GFP-PDZ, a gift from the Lukas Kapitein lab (*27*), was used as a template to generate pSBtet Kif5b-GFP-pMagFast1_(3x)_ [last module synthesized and tandemly added via Spe I, Not I, Asc I, and Kpn I restriction sites for possible optogenetic manipulation (*69*)]. The localization-based probe for active RhoA (tandem RBD, four in total separated by a linker) together with iRFP670 (three repeats to increase sensitivity) was synthetized as proposed (*52*) and inserted into the pCDH vector via Xba I and Sal I restriction sites to create pCDH_iRFP670_3x_-RBD_4x_. pLVX-GFP-FMNL1β was a gift from the Blystone lab (*70*). All constructs were verified first by restriction analysis and then by sequencing.

### Preparation of GFP-MNPs

The construct used to purify modified GFP {pET21_H6-mEGFP-Mms6[112 to 132]} before functionalizing nanoparticles to prepare GFP-MNPs has been previously described (*32*). The construct comprises a 6xHis-tag fused with the N terminus of mEGFP, connected by a seven–amino acid linker with the iron-binding domain of Mms6 (22 C-terminal amino acids MKSRDIESAQSDEEVELRDALA). The pET21_H6-mEGFP-Mms6[112 to 132] construct was transformed into *Escherichia coli* BL21-CodonPlus-RIL (Agilent) for preparative overexpression of H6-mEGFP-Mms6[112 to 132]. Cells were grown at 37°C in LB medium supplemented with ampicillin (100 μg/ml) and chloramphenicol (32 μg/ml). Subsequently, the expression of H6-mEGFP-Mms6[112 to 132] was induced with 0.5 mM isopropyl β-d-1-thiogalactopyranoside (Thermo Fisher Scientific) at an optical density at 600 nm of 0.6, followed by culturing at 16°C overnight. The harvested cells (>4000 g for 10 min) were resuspended in lysis buffer (50 mM Hepes, 150 mM NaCl, and 8 M urea, pH 7.5 to 8.0), followed by tip sonication (4 × 3 min; 50% duty cycle, Sonifier 250, Branson) and centrifugation (20,000*g*, 30 min) to pellet insoluble material. The protein was first purified using immobilized metal affinity chromatography by loading the supernatant on a 5-ml hiTrap chelating HP column loaded with nickel(II) chloride and equilibrated in 20 mM Hepes and 150 mM NaCl, pH 8.0 (HBS). The protein was eluted with HBS and 500 mM imidazole, pH 8.0, using a linear gradient that spanned 10 times the bed volume. Subsequent purification based on size exclusion chromatography was performed, if needed (aggregates observed upon 1-hour incubation with 8 M urea, which leads to a profound increase in total protein yield), using a fast protein liquid chromatography system (Äkta Explorer, GE Healthcare) by loading protein solution onto a size exclusion chromatography column (HiLoad 16/60 Superdex 75, prep grade or HiLoad 26/60 Superdex 75, prep grade) that had been equilibrated in HBS. Protein integrity purity and integrity were confirmed by 12% SDS–polyacrylamide gel electrophoresis (SDS-PAGE; expected size, 31.2 kDa).

To prepare functional GFP-MNPs, characterized and described previously (*32*), purified H6-mEGFP-Mms6[112 to 132] (HBS buffer) was first transferred into water (pH 7.0) using a buffer exchange column (PD10) and the resulted concentration was increased (>100 μM; molar extinction coefficient at 488 nm = 56,000), if below, by ultrafiltration [Amicon Ultra-4, 10-kDa cutoff, 20 mM Hepes, pH 7.5, room temperature (RT)]. Magnetic core particles with average diameters of 12 nm (iron concentration of 900 μM, pH 2), synthesized as described before (*32*), were diluted to a final 90 μM concentration (water, pH 2) and sonicated (ultrasound bath, 10 min, 10° to 15°C). Immediately after, these nanoparticles were added to a >75-fold molar excess of purified H6-mEGFP-Mms6[112 to 132] (>100 μM; water, pH 7.0), followed by additional sonication (ultrasound bath, 5 min, 15° to 20°C), and incubated for >1 hour (RT). The protein excess was removed by ultrafiltration (Amicon Ultra-4, 100-kDa cutoff, 20 mM Hepes, pH 7.5, RT) until the filtrate was devoid of unbound coating protein. Last, GFP-MNPs were incubated in PFA for 1 hour (4% PFA in 20 mM Hepes, RT), followed by extensive washing (>3×) using ultrafiltration (Amicon Ultra-4, 100-kDa cutoff, 20 mM Hepes, pH 7.5, RT). The final GFP-MNPs [20 mM Hepes, pH 7.5, and ciprofloxacin (10 μg/ml) to prevent bacterial growth] were stored for up to 14 days at RT before live-cell experiments, and no noticeable deterioration in magnetic attraction was observed. Directly before use inside living cells, GFP-MNPs were centrifuged to remove aggregates (2500*g* to 4000*g*, 5 min, RT).

### Cell culture

The ovarian cancer cell line A2780-DNA3 (*11*) was cultured in RPMI-1640 medium (R8758, Sigma-Aldrich). Telomerase-immortalized fibroblasts (TIFs) (*11*)) and human embryonic kidney 293T cells were cultured in Dulbecco’s modified Eagle’s medium (DMEM; D5796, Sigma-Aldrich). All cell culture media were supplemented with 10% (v/v) fetal bovine serum and ciprofloxacin (0.01 mg/ml; Sigma-Aldrich), and cells were maintained at 37°C in a humidified atmosphere with 5% (v/v) CO_2_. The cells were confirmed to be free of mycoplasma contamination by polymerase chain reaction (PCR) analysis. A2780 cells were selected for antibiotic resistance as follows: puromycin (0.5 to 1 μg/ml); blasticidin (5 μg/ml).

### CRISPR-Cas9 ribonucleoprotein–based transfection and homologous recombination

CRISPR-Cas9–based modifications (FMNL1), including the DExCon approach (Rab25), were performed as previously described in detail (*21*). ssDNA coding sequences, flanked by specific homologous sequences (FMNL1 exon 2 and Rab25 exon 1), were used to generate a specific CRISPR-based knockin outcome, eliminating the necessity for clonal selection (*21*, *71*). Purified ssDNAs were prepared as previously described (*21*, *72*) using double-stranded DNA (pJET-24_Rab25 DExCon, pJET-78-FMNL1-HR-SFFV-BlaR, and pJET-77-FMNL1-HR-SFFV-PuroR) and PCR with reverse and biotinylated forward primers (primer sequences in table S3). The biotinylated PCR product was purified using Dynabeads MyOne Streptavidin C1 (cat. no. 65001, Thermo Fisher Scientific), anti-sense ssDNA strand eluted by 20 mM NaOH (later neutralized by HCl), and final ssDNA purified using SPRI beads (AMPure XP, Agencourt). The anti-sense ssDNA strand was verified by agarose gel electrophoresis (denaturated) and/or sequencing and used for knockin [detailed protocol provided in https://doi.org/10.48420/16878859 (*21*)]. High-fidelity Cas9 (Alt-R S.p. HiFi Cas9 Nuclease V3, IDT) and guide RNA (crRNA:tracrRNA, synthetized by IDT) were delivered together as a ribonucleoprotein (final 10 nM) with an ssDNA donor (300 to 530b homologous arms; 15 to 50 ng) via CrisperMAX (Thermo Fisher Scientific) combined with nanoparticles for magnetofection (for sequences, see table S2). The Combimag nanoparticles (OZBiosciences) were used in accordance with the manufacturer’s instructions using a magnetic plate.

For the DExCon approach, A2780 cells stably expressing tagBFP-T2A-TetOn3G (sorted for cytoplasmic tagBFP, which is separated from the TetOn3G transactivator by the self-cleaving peptide T2A) were used to integrate TRE3GS-NB^GFP^-mCherry into the Rab25 exon1 locus, fluorescence-activated cell sorting (FACS) sorted (upon reversible dox-mediated Rab25 reactivation; see fig. S1), and specific knockin validated by fluorescence microscopy, Western blot, and sequencing of PCR-amplified genomic DNA as previously described [primers are listed in table S3; details published in (*21*)].

To inactivate the human FMNL1 gene in A2780 cells, we used our previously published strategy for gene inactivation, which obviates the necessity for clonal selection through the precise integration of the puromycin (PuroR) and/or blasticidine (BlaR) resistance cassette (exon-SFFV-PuroR/BlaR-polyA-exon) (*21*). For data details, see extended fig. S10 (C to H) accessible from https://doi.org/10.6084/m9.figshare.28554908; supporting text below, while we were able to readily prepare FMNL−/+ heterozygotes, the use of any BlaR/PuroR combination proved unsuccessful in obtaining FMNL1−/− homozygotes. This finding suggests that FMNL1 is essential for A2780 survival, as has been demonstrated in mice (*73*), or that the efficiency of the homozygous knockin is too low. Therefore, we generated clones of A2780 (33 in total) with one allele targeted by ssDNA carrying puroR followed by puromycin treatment in the hope that the second allele would be inactivated by the more common indel-rich out-of-frame nonhomologous end-joining pathway. Unexpectedly, all puromycin-surviving clones were once again heterozygous, with the FMNL1 protein oscillating around the expected size on the Western blot, indicating the presence of in-frame indels. However, these indels were always in frame, suggesting that FMNL1 plays an essential role in A2780.

### DNA transfection and siRNA-mediated protein depletion

Unless otherwise stated, Lipofectamine 2000 (Invitrogen) was used to transiently transfect plasmid DNA into A2780 cells in accordance with the manufacturer’s instructions. A2780 cells transfected with pSB-pSBtet-Kif5b-GFP-pMagFast1 were codelivered with Sleeping Beauty transposase SB100X in the ratio as suggested (*68*) followed by puromycin selection (1 μg/ml) and FACS [BFP/GFP; dox (500 ng/ml) treated, 48 hours].

All FMNL1-targeting siRNAs were nucleofected into A2780 cells or A2780 FMNL1−/+ (PuroR/+) harvested from a subconfluent 150-mm cell culture dish using a nucleofector (Amaxa, Lonza) with solution T (VCA-1002, Lonza), program A-23, and 5 μl of 20 μM siRNA (total of 100 pmol; 1 μM) as per the manufacturer’s instructions. Silencer select siRNA reagents, with locked nucleic acid chemical modifications to increase specificity and stability, were purchased from Invitrogen: negative control (NS) (4390843); FMNL1 siRNA nos. s26 (4392420, ID: s2226), s27 (4392420, ID: s2227), and s28 (4392420, ID: s2228). FMNL1 siRNA nos. s26, s27, and s28 were combined together to create a smart pool.

### Lentivirus packaging generation

Lentiviral constructs (pCDH, pLenti, and pLVX) coding different FMNL1 and Rab25 fusion constructs (see table S1) were used to generate stable cell lines as follows: Lentiviral particles were produced in human embryonic kidney 293T cells via a polyethylenimine-mediated transfection with packaging plasmids pM2G and psPAX2 with a lentiviral vector (pCDH, pLenti, and pLVX) in a ratio of 1.5:1:2. Supernatants were collected at 94 hours posttransfection, filtered through a 0.45-μm filter, and added to target cells. Transduced cells were selected by appropriate antibiotics (GFP-FMNL1β; puromycin) or sorted by FACS (Aria II, BD; iRFP670-Lifeact, NB^GFP^-mCherry fusions, tagBFP for T2A-mycBirA* fusion protein constructs and for tagBFP-T2A-TetOn3G before Rab25 DExCon modification). NB^GFP^-mCherry-fused Ctrl, Rab25 dC, or Rab25 wt was sorted for matched expression levels.

### CDM preparation and wide-field live-cell imaging

CDMs were generated as previously described (*11*, *74*) on either 40-mm coverslips (#1.5H; for magnetic live imaging experiments) or tissue-culture plastic 12-well plates (for long-term time-lapse microscopy). Briefly, plates were coated with 0.2% gelatin (v/v; Sigma-Aldrich), cross-linked with 1% glutaraldehyde (v/v; Sigma-Aldrich), and quenched with 1 M glycine (Thermo Fisher Scientific) in phosphate-buffered saline (PBS) before washing and equilibration in DMEM. TIFs were plated onto the prepared plates at a density that would ensure confluency within the following day or two. TIFs were cultivated for a period of 8 to 10 days, with the medium being replaced with DMEM supplemented with ascorbic acid (50 μg/ml; Sigma-Aldrich) 24 hours after seeding and subsequently every 48 hours. Cells were denuded with extraction buffer [0.5% (v/v) Triton X-100; 20 mM ammonium hydroxide (NH_4_OH)] to leave only the matrix and washed twice with PBS+ (Dulbecco’s PBS with CaCl_2_ and MgCl_2_, Sigma-Aldrich). Last, the matrices were incubated with deoxyribonuclease I (10 μg/ml; Roche) and washed three times with PBS+ before A2780 cells were plated at a low density into a 12-well plate or 40-mm coverslip inserted into a 60-mm dish. The cells were then allowed to spread and start migrating overnight (40-mm coverslip; see the “Spinning-disk confocal cell imaging and analysis” section) or at least 4 hours before being imaged (12-well plate). The images were obtained using the confocal spinning-disk microscopes described below (magnetic experiments; see the “Spinning disc confocal cell imaging and analysis” section) or on an DMi8 inverted wide-field microscope (Leica) using either a 20×/0.40L Plan Fluor dry-corrected or 10×/0.25N Plan Fluor objective and a light-emitting diode light source for transmitted light. Images were collected using a DFC 9000–monochromatic scientific complementary metal-oxide semiconductor (CMOS) camera, and cells were maintained at 37°C and 5% CO_2_ for the duration of imaging. LAS X software (Leica) was used to acquire images of multiple positions per well, every 5 min for 16 hours. For cell migration analysis, at least 70 cells (in total) were individually manually tracked per condition from three independent replicates using CellTracker software (*75*), and this was used to calculate the average cell speed and persistence, where persistence is equal to the path length divided by the Euclidean distance.

Additional fluorescence imaging and bright-field imaging were conducted using the aforementioned wide-field microscope with a 10×/0.25N Plan Fluor objective on an FN-coated 96-well plate (Cellvis, #1.5H cover glass) with FluoroBrite DMEM (Thermo Fisher Scientific) supplemented with 20 mM GlutaMax (Thermo Fisher Scientific) and 10% (v/v) fetal bovine serum. LAS X software (Leica) was used to acquire images of two to four random positions per well (cell length analysis), every 10 min for 16 hours (protrusion lifetime analysis). Treatments and analyses are specified in the figure legends. The protrusion lifetime was scored from F-actin (Lifeact-iRFP670). For the protrusion lifetime, the lifetime measurement was made from randomly selected narrow protrusions (width below 5 μm; one to three per cell) from the time they start to appear until the moment they disappeared or widened >5 μm.

### Magnetic tweezers

Magnetic tweezers were home-built by combining a sharp paramagnetic tip and neodymium magnets as follows: To create a sharp paramagnetic tip, an iron wire (0.4-mm diameter) was pulled in the flame of a Bunsen burner. The string was pulled at a slow rate, resulting in the formation of two sharp extremities with a diameter of 20 μm. These were used as paramagnetic tips on a permanent magnet in two distinct configurations but generated the same steady-state gradient profiles of GFP-MNPs at a distance of 0 to 150 μm (measured in fig. S2): The sharp tip (length of 1.5 mm) was placed on top of a tower of small resizing magnets made of neodymium iron boron N-45 (dimensions diameter by height: 1 by 2, 3 by 6, 5 by 10, 8 by 30, and 10 by 20; Supermagnete, Gottmadingen, Germany) or similarly as described (*31*, *32*) by attaching a paramagnetic tip (length of 3 to 5 mm) on top of a block neodymium magnet (5 by 1.5 by 1 mm, N-45, Supermagnete) and gluing it into a plastic pipette tip, ensuring that the tip extends 0.5 mm beyond the end of the magnet. Subsequently, magnetic tweezers were attached to the micromanipulation apparatus (mikromanipulator 5171 or InjectMan NI2), which was situated in close proximity to the Zeiss microscope with a CSU-X1 spinning-disk confocal (Yokagowa) or mounted on a Leica microscope equipped with a Dragonfly 503 spinning-disk confocal (Andor). Both the apparatus and microscope were surrounded by heating chambers.

### Microinjection

Before microinjection, cells were seeded on 40-mm coverslips (#1.5H) with FN coating or with prepared CDMs and inserted into a nonmagnetic FCS2 chamber (Bioptechs, US) or into newly designed custom-made lidded imaging chamber insert (Vivanto, Czechia; available for purchase). Subsequently, these were inserted into a Zeiss microscope with a CSU-X1 spinning-disk confocal (Yokagowa) or a Leica microscope equipped with a Dragonfly 503 spinning-disk confocal (Andor Technologies). GFP-MNPs (0.8 to 2 μM solution in 20 mM Hepes) were injected into cells using commercial injection capillaries (Femtotip, 0.5-μm inner diameter and 1.0-μm outer diameter; Eppendorf) and a micromanipulation system (mikromanipulator 5171 or InjectMan NI2 and 10 FemtoJet Express, Eppendorf) in semiautomatic mode. The capillary pressure was maintained at a constant level of 18 to 20 psi, while the injection pressure (80 to 180 psi) and time (0.3 to 2 s) were subject to inverse variation. To microinject cells migrating in CDMs, higher injection pressure was usually needed (180 psi). Following microinjection, the cells were allowed to recover (5 to 10 min) before being imaged. The microinjected cells were then subjected to a quality control assessment to ascertain their unharmed capacity for migration and to check the quantity and quality of the microinjected GFP-MNPs. Subsequently, the injection capillary was replaced with magnetic tweezers followed by live-cell imaging.

### Spinning-disk confocal cell imaging and analysis

Magnetic spatiotemporal control of GFP-MNPs in living cells was performed with A2780 cells (±expressing NB^GFP^-mCherry fusion protein variant) situated on FN-coated or CDM-covered 40-mm coverslips (#1.5H), inserted into the aforementioned chamber insert (see the “Microinjection” section) and the aforementioned micromanipulator with magnetic tweezers (see the “Magnetic tweezers” section) at a distance of 30 to 120 μm from cells. GFP-MNPs were delivered by microinjection (see the “Microinjection” section). Imaging was recorded using a CSU-X1 spinning-disk confocal (Yokagowa) on a Zeiss Axio-Observer Z1 microscope or a Dragonfly (Andor Technologies) spinning-disk confocal Leica microscope with an APO 40×/1.25–numerical aperture oil-immersion or APO 63×/1.20 water-immersion objectives. Microscopes were equipped with an Evolve electron-multiplying charge-coupled device camera (Photometrics) or a Zyla 4.2 PLUS scientific CMOS camera, both with a motorized XYZ stage and 405, 488, 561, and 637 lasers. Fluorescence images were captured with the appropriate excitation/emission spectrum, and a white light-emitting diode source was used for transmitted light to monitor the position of the magnetic tip. Images were captured using SlideBook 6.0 (3i) or Fusion software. The objective drift was corrected automatically using the HWada coordinate shift plug-in in Fiji-ImageJ (NIH, US) wherever necessary. During the imaging and manipulation processes, the cells were maintained in a 1× Opti-Klear medium (6 ml; Marker Gene Technologies Inc.) supplemented with 10% (v/v) fetal bovine serum in a heating chamber. The time interval acquisition was always set according to the specific requirements of the experiment, with a duration of either 2 s for rapid kinetics and magnetic tip positioning or 30 s for long-term imaging. The laser powers were set to an optimal level that would not induce stress on the cells. Laser-based (point visiting) Definite Focus was used to maintain the correct plane in focus, with manual readjustment if refocus occurred because of magnetic tip movement. On each day of the experiment, the functionality of the magnetic tweezers and GFP-MNPs was initially evaluated on control A2780 cells (which express NBGFP-mCherry). A *Z* stack was acquired before and after 10 to 60 min of manipulation and used for the analysis of protrusion growth (LifeAct reporter), as described in Fig. 1 (A to D), using Fiji-ImageJ (NIH, US). Only cells with visible GFP-MNP relocalization/enrichment were included in the analysis, and protrusions were induced by Rab25 before various inhibitor treatments to observe their effects. Magnetic attraction and release kinetics of NB^GFP^-mCherry-(X) and GFP-MNPs in living cells were quantified as described in Fig. 2 (B and C). Note that NB^GFP^-mCherry-Rab25 wt–overexpressing cells exhibiting a higher proportion of diffusively localized Rab25 with fast kinetics (~25%; not observed with A2780 DExCon-modified NB^GFP^-mCherry-Rab25 cells) were not included in this analysis. The relative stress maps were analyzed using bright-field images of cells migrating in CDMs, taken before and after the magnetic attraction of GFP-MNPs via the particle image velocimetry analysis plug-in (*76*) in Fiji-ImageJ. Kymographs were created from time-lapse images using KymoToolBox in Fiji-ImageJ. The AI-based Kymobutler tool (*77*) was used to analyze particle traces in kymographs via the cloud application or as a plug-in in Fiji-ImageJ.

Additional fluorescence imaging was conducted using the aforementioned spinning-disk confocal microscopes with a 63×/1.40 Plan-Apochromat objective on randomly chosen representative fixed (see the “Immunostaining” section) or live cells situated on an FN-coated μ-Plate 96 plate (#1.5 IbiTreat) or FN-coated 96-well plate (Cellvis, #1.5H cover glass) and analyzed using Fiji-ImageJ (NIH, US), as specified in the figure legends. For colocalization analysis, Colocalization finder plugin in Fiji-ImageJ was used to generate Pearson’s correlation coefficient (no thresholding and noise subtraction using the ScatterPlot function).

### Magnetic force calculation

Quantification of forces exerted by magnetic manipulation was quantified from images taken by spinning-disk confocal microscopes (see the “Spinning-disk confocal cell imaging and analysis” section). The rosette plot was generated in Prism 8.0 (GraphPad Prism software). The angles between cells with a GFP-MNP gradient and a magnet-aligned line extending from the magnet tip were divided into 12 30° segments. The relative sizes of the triangular segments are directly proportional to the number of cells with angles contained in each bin. The attraction and relaxation kinetics and the applied force on GFP-MNPs were determined in the cell cytoplasm at the steady state of attraction. The distance-dependent decrease in particle intensity *I*(*r*) was fitted exponentially. The theoretical profile was assumed to follow the Boltzmann law

$$I\left(r\right)=e^{-\frac{\mathrm{Fr}}{k_{B}T}}$$ (1)

The applied force *F* was determined from the fitted exponential decay ( $k_{B}$ : Boltzmann constant; *T*: temperature).

### Immunostaining

For all antibody staining procedures, cells were fixed in 4% (w/v) PFA (Sigma-Aldrich) for 10 to 15 min at RT (or 2% PFA for 10 min on ice to preserve an endogenous fluorescent signal, e.g., mCherry), followed by mild permeabilization with 0.1% Triton X-100 (Sigma-Aldrich, 2 to 10 min) in PBS (Dulbecco’s PBS) or 0.1% saponin (Sigma-Aldrich) added to all antibody incubation steps (to better preserve endosomes). Fixed cells were blocked in 3% (w/v) bovine serum albumin (BSA) (Sigma-Aldrich) in PBS and incubated with corresponding primary and secondary antibodies diluted in 3% BSA in PBS for 1 to 2 hours at RT. Cells were washed three times with PBS between each incubation step and once with double-distilled H_2_O before mounting with either ProLong Diamond Antifade Mountant (Molecular Probes, Invitrogen) or ProLong Gold Antifade Mountant (Molecular Probes, Invitrogen).

### Antibodies and reagents

The following antibodies and reagents were used (details in table S4): α-tubulin [mouse monoclonal antibody (mAb) DM1A, no. ab7291], Rab25 (rabbit mAb DH46P, no. 13048S), RFP (rat mAb 5F8), GFP (mouse mAb 1E10H7, no. 66002-1-Ig), FMNL1 [rabbit polyclonal antibody (pAb), no. 27834-1-AP], GAPDH (glyceraldehyde-3-phosphate dehydrogenase; rabbit pAb, no. G9545), integrin β1 (mouse mAb 18/CD29, no. 610467; immunoblot), integrin β1 (mouse mAb, 5PD2; blocking), integrin β1 (rat mAb, AIIB2; DSHB; blocking), IgG (immunoglobulin G; goat pAb; no. AB_2337925), and Streptavidin DyLight-800 (1:5000) (Thermo Fisher Scientific). Fluorescent secondary antibodies (Jackson ImmunoResearch Laboratories or LI-COR, Invitrogen) were used as recommended by the manufacturer. The reagents used were puromycin dihydrochloride (Thermo Fisher Scientific), blasticidin S HCl (Gibco), FN (Sigma-Aldrich), Dynabeads MyOne Streptavidin C1 (Thermo Fisher Scientific), Agencourt AMPure XP (Beckman Coulter), and MagReSyn Streptavidin microspheres (ReSyn Biosciences). Dox hydrochloride (Sigma-Aldrich) was dissolved at 250 μg/ml in water (stored at −20°C in aliquots) and used within 14 days if kept at 4°C.

### Knock-sideways imaging and analysis

A2780 cells were nucleofected (VCA-1002, Lonza) with 2 μg of pMito-iRFP670-FRB and 1 μg of the required GFP-FKBP (Ctrl or -Rab25) using programme A-23 (Lonza). The following day, rapamycin was added to the cell growth medium at a final concentration of 200 nM for 4 hours. Cells were then fixed and stained (see the “Immunostaining” section) for FMNL1 (rabbit pAb, no. 27834-1-AP; 1:400), followed by Cy3 secondary antibody detection (Molecular Devices). *Z*-Stacks were captured using a CSU-X1 spinning disk (Yokogawa) on a Zeiss Axio-Observer Z1 microscope with a 63×/1.46 α Plan-Apochromat objective, a Prime 95B scientific CMOS camera (Photometrics) and Slidebook software (3i). All images were processed using ImageJ, and relocalization of GFP-FKBP or GFP-FKBP-Rab25 proteins to mitochondria was analyzed using the Colocalization finder plug-in and scored on the basis of Pearson’s correlation coefficient (no thresholding and noise subtraction using the ScatterPlot function). For FMNL1 redistribution, a custom-made script was used to create a mitochondrial mask (available at https://doi.org/10.48420/19391597) and the mean pixel intensity of the FMNL1 protein was compared between that in mitochondria and that in the cell body.

### Proliferation

The cell proliferation rate was analyzed via live, real-time, label-free, image-based measurements using the xCELLigence RTCA eSight system (Agilent) situated in a temperature- and CO_2_-controlled incubator. A total of 5000 cells was seeded into each well of the E-Plate VIEW 96 (quadruplicates per condition) and automatically imaged at 2-hour intervals over a 70-hour period using a 10× objective. Subsequently, RTCA eSight software was trained to create object masks to determine cell numbers in the field of view.

### STEM tomography

GFP-MNPs (1.1 μM, Opti-MEM) were electroporated [0.2-cm aluminum cuvettes (100 μl), Lonza] into A2780 DExCon-modified NB^GFP^-mCherry-Rab25 cells (pretreated with dox for >94 hours; 250 ng/ml) by using an Amaxa Nucleofector II Electroporation machine (Lonza) on program A-023. This approach results in a high efficiency of GFP-MNP delivery (*32*), and the ratio of GFP-MNPs:Rab25 was close to 1:1 or less, ensuring that most GFP-MNPs were Rab25-bound. Cells were then left to spread (3 hours) on an FN-coated 12-mm round coverslip before rinsing with Sörensen’s phosphate buffer (SB; 0.1 M, pH 7.2 to 7.4) prewarmed to 37°C and fixing in a mixture of 1% glutaraldehyde and 2.5% PFA in SB for 30 min at RT and then overnight at 4°C. The next day, fixatives were washed out with SB and cells were postfixed with a 1% OsO_4_ solution in SB for 1 hour in the dark. Samples were washed again with SB and Milli-Q water and dehydrated with a series of water/acetone mixtures finished with dried acetone. Last, embedding was performed into Epon-Durcupan resin, followed by polymerization at 60°C for 3 days. Embedded samples were cut into semithin sections of 500 nm in thickness and placed on a gilded copper slot (2 by 0.5 mm) without any supporting film so that the section exceeds the whole slot and is attached to its rim. To enhance the contrast, the section on a slot was immersed into a droplet of a 2% uranyl acetate/water solution for 30 min. However, such a sample is still too fragile for STEM tomography. To make it more durable, it was coated with a 4-nm carbon layer from both sides (Leica EM ACE600 sputter coater). Last, fiducial markers (6-nm gold nanoparticles) were introduced on both sides of the section to facilitate the tilt series (TS) reconstruction. Cells positive for GFP-MNPs and high-contrast recycling endosomes (50 to 200 nm) were first found by standard TEM imaging in the Jeol JEM-1400 flash transmission electron microscope equipped with a tungsten cathode and a bottom-mounted FLASH 2kx2k CMOS camera and operated at 80 kV. The sample was mapped with Limitless Panorama software at high magnifications (up to ×30,000), and the stitched map was used to refind the regions of interest in a high-end TEM Jeol JEM-F200. Following the acquired Limitless Panorama map, the regions of interest were located in the Jeol JEM-F200-high-end S/TEM electron microscope equipped with a cold field emission gun and a TVIPS TemCam–XF416 cooled 4kx4k CMOS camera and operated at 200 kV. The series of tilted images (TS) of individual regions within the section was acquired in the scanning mode (STEM), in which a dark field STEM detector was used. TS acquisition was controlled by SerialEM software (program developed by D. Mastronarde, University of Colorado) including the STEM tomography module. The TS was collected automatically in one direction from −60° to +60° with a step of 1°, the recorded image time frame was 40 s, and the used magnification was ×100,000. Reconstruction of the obtained TS was performed in a module of IMOD software called “Etomo,” where the fiducial markers were used for the fine alignment of TS frames. TS frames were binned to a final size of 1024 by 1024 pixels, the reconstruction itself was performed with a SIRT-like filter of 15, and the LUT of the finished tomogram was inverted to look like a bright-field image—this sequence generated the best image quality to distinguish the studied objects for a consequent segmentation. GFP-MNPs and endosomes were manually segmented using MATLAB-based Microscopy Image Browser software (*78*) and visualized using Imaris (Bitplane).

### Proximity labeling

Cells stably expressing mycBirA* fusion protein constructs (see the “Lentivirus packaging generation” section) were plated onto tissue culture plates (1 by 100 mm for FMNL1 detection or 4 by 150 mm for integrin β1) at a density to ensure subconfluency the following day, and BioID was performed as described previously (*22*). Briefly, more than 4 hours after cells were plated, 1 μM biotin (Sigma-Aldrich) was added to the cell culture medium and cells were incubated for 16 hours. Subsequently, cells were washed in PBS before addition of the BioID lysis buffer [50 mM tris, pH 7.4, 500 mM NaCl, 5 mM EDTA, 0.4% SDS, and 1 mM dithiothreitol, supplemented with the protease inhibitors aprotinin (100 μg/ml; Sigma-Aldrich), leupeptin (100 μg/ml; Sigma-Aldrich), 0.5 mM 4-(2-aminoethyl)benzenesulfonyl fluoride hydrochloride (Calbiochem), 50 μM PD150606 (Calbiochem), and 500 μM ALLN (Calbiochem); RT]. Cell lysates were collected using a cell scraper, mixed with an equal volume of 50 mM tris (pH 7.4) and Triton X-100 (final concentration of 2%, Sigma-Aldrich), and then lysed with a needle. Lysates, clarified by centrifugation (16,000*g* for 10 min at 4°C), were equalized to the total protein amount and incubated with 15 μl of MagReSyn Streptavidin microspheres (per 10-cm plate; ReSyn Biosciences) overnight at 4°C with rotation. Beads were extensively washed: twice in wash buffer 1 (BioID lysis buffer with 2% SDS), once in wash buffer 2 (0.1% deoxycholate, 1% Triton X-100, 500 mM NaCl, 1 mM EDTA, and 50 mM Hepes, pH 7.4), and once in wash buffer 3 (0.5% NP-40, 0.5% deoxycholate, 1 mM EDTA, and 10 mM tris, pH 8.1). Bound proteins were eluted by the addition of 40 μl of 2× sample buffer saturated with biotin (250 mM tris-HCl, pH 6.8, 2% SDS, 10% glycerol, 0.2% bromophenol blue, and 20 mM dithiothreitol, with >1 mM biotin) at 70°C for 5 min. Last, eluted samples were analyzed by Western blot analysis.

### SDS-PAGE and Western blotting

Cells were lysed in a denaturing lysis buffer [2% SDS, 20% glycerol, 120 mM tris-HCl (pH 6.8), and 0.1% bromophenol blue] and heated for 10 min at 98°C followed by SDS-PAGE under denaturing conditions (4 to 12% bis-tris gels; Invitrogen). Subsequently, resolved proteins were transferred to a nitrocellulose membrane using the Trans-Blot Turbo Transfer System (Bio-Rad), then blocked with 4% BSA-TBS (tris-buffered saline; 1 hour), and incubated overnight at 4°C with the appropriate primary antibody in 2 to 3% BSA TBST (2 to 3% BSA in TBS with 0.05% Tween 20; overnight at 4°C) and fluorophore-conjugated secondary antibody (2.5% milk in TBS with 0.05% Tween 20; 1 hour at RT). The membranes were imaged using an infrared imaging system (Odyssey; LI-COR Biosciences).

### RNA extraction, reverse transcription, and PCR

RNA extraction and reverse transcription were performed as previously described (*21*). PCR was conducted using PfuX7 home-made polymerase (*79*), in accordance with the standard Pfu polymerase protocol. Reactions were performed with 500 nM primers designed for this study (listed in table S3). PCR products were run on a 2% agarose gel with SYBR green to check primer specificity and for quantification of band intensities.

### Statistical analysis and bioinformatics

The data were tested for normality and analyzed using one-way or two-way analysis of variance (ANOVA) with corresponding post hoc tests as indicated in legends. If the data were not normally distributed, ANOVA on ranks was used instead. All statistical analyses were done with GraphPad Prism software. *P* values are described in relevant figure legends, where ***P < 0.001, **P < 0.01, and *P < 0.05. The number of independent experiments (*N*) and data points (*n*) is indicated in each of the figure legends. In the violin plots showing the whole data distribution, data are presented using SuperPlots with the superposition of the medians of each experiment shown as dots.

Bioinformatic reanalysis of Rab25-BioID (proximity labeling) was done on the dataset available via the PRIDE partner repository with the dataset identifier PXD033693 (*22*). Proteins exhibiting a minimum of 1.8-fold MS1 intensity–based (mass spectrometry–based label-free quantification) increase in the Rab25-BioID sample relative to the BioID control sample (BirA alone), observed at least in three of four biological replicates, were considered to be statistically significant. The selected Rab25proximity interactome was visualized by STRING protein-protein interaction analysis (https://string-db.org/).

## Supplementary Materials

### The PDF file includes:

Figs. S1 to S12 Tables S1 to S5 Supplementary Text Legends for movies S1 to S10 Legends for extended supplementary movies S11 to S22 References
