# Supplementary material for: Live-cell magnetic manipulation of recycling endosomes reveals their direct effect on actin protrusions to promote invasive migration
Source: Sci Adv. 2025 Jul 4;11(27):eadu6361. doi: 10.1126/sciadv.adu6361 (PMC12227070; doi:10.1126/sciadv.adu6361)
Supplement: Supplementary file 1 — Figs. S1 to S12 Tables S1 to S5 Supplementary Text Legends for movies S1 to S10 Legends for extended supplementary movies S11 to S22 References [file sciadv.adu6361_sm.pdf]

Science Advances
AAAS

Supplementary Materials for

Live-cell magnetic manipulation of recycling endosomes reveals their direct effect on actin protrusions to promote invasive migration

Jakub Gemperle *et al.*

Corresponding author: Patrick Caswell, patrick.caswell@manchester.ac.uk;
Jakub Gemperle, jakub.gemperle@img.cas.cz

Sci. Adv. **11**, eadu6361 (2025)
DOI: 10.1126/sciadv.adu6361

The PDF file includes:

Figs. S1 to S12
Tables S1 to S5
Supplementary Text
Legends for movies S1 to S10
Legends for extended supplementary movies S11 to S22
References

Other Supplementary Material for this manuscript includes the following:

Movies S1 to S10

Supplementary Figures

fig. S1

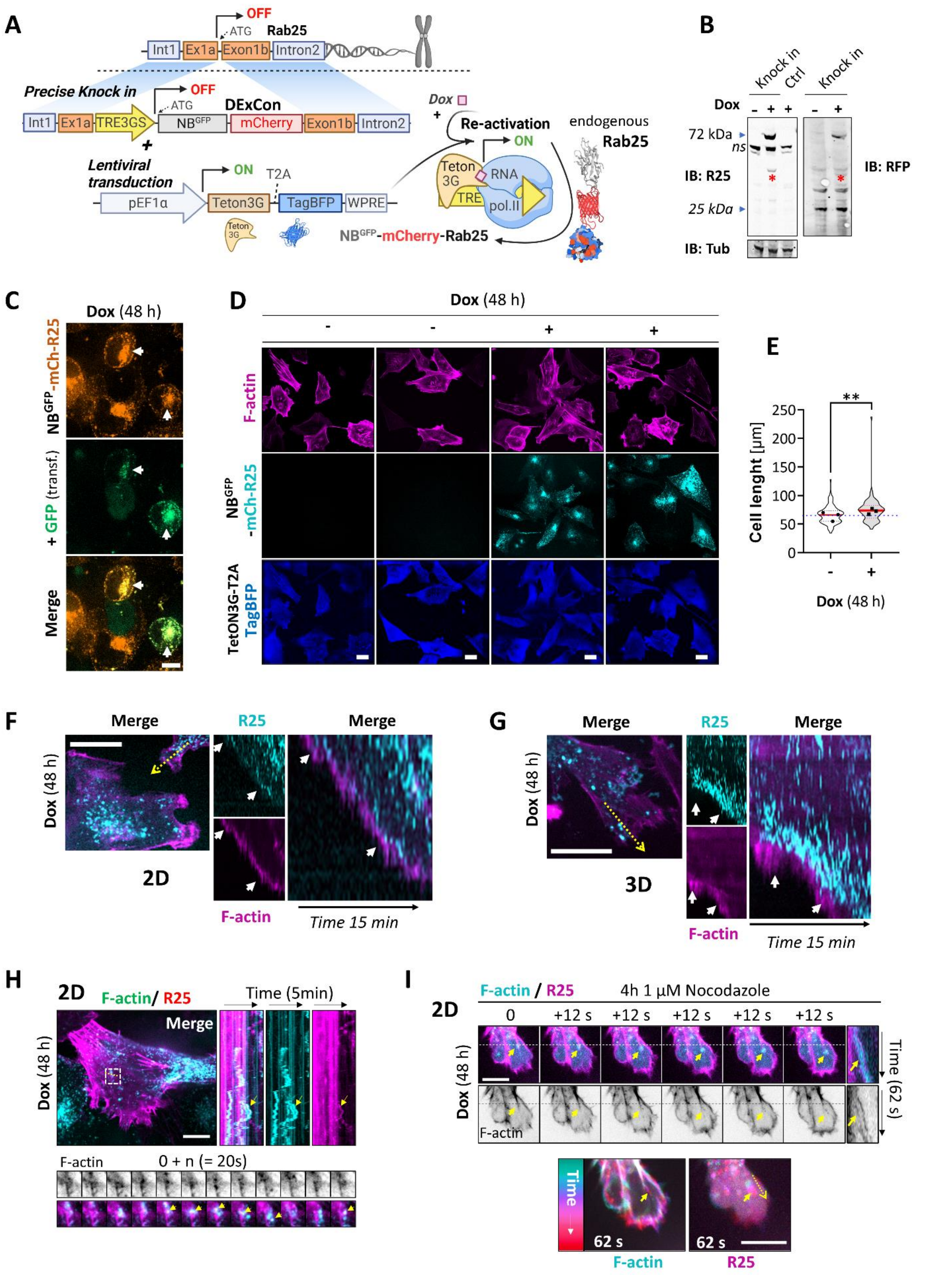

Figure S1. Positive correlation between the trafficking of Rab25 endosomes towards the plasma membrane (PM) and actin polymerisation. A) Schematic representation of DExCon approach based on CRISPR/Cas9 editing used

Figure S1. (Continued from previous page.) to generate A2780 DExCon-modified NB^{GFP}-mCherry-Rab25 cells ((*21*). TetOn3G transactivator is co-expressed with TagBFP from constitutive promoter and separated by the self-cleaving peptide T2A. Upon dox treatment TetOn3G binds TRE3GS promoter to re-activate expression of modified Rab25 from endogenous locus; see Methods for more details). Created with BioRender.com (https://BioRender.com/ds02ifg). **B)** Immunoblots A2780 DExCon-modified NB^{GFP}-mCherry-Rab25 cells (± dox 48h; 250 ng/ml) stably expressing Lifeact-iRFP670. Fluorescent antibodies: anti-Rab25 (R25), anti-mCherry (RFP) shown as black and white. Tubulin (Tub), loading control. Ctrl = un-modified wt A2780; arrow, size of predicted endogenous Rab25 (bottom) or dox-reactivated modified NB^{GFP}-mCherry-Rab25 (top); ns, non-specific band; star, mCherry/iRFP670 fusion band caused by hydrolyzed C==N acylimine bond due to boiling (*80*). **C, D, F, G, H, I)** Representative confocal spinning-disk live cell images of A2780 DExCon-modified NB^{GFP}-mCherry-Rab25 (R25; dox 48 h, 250 ng/ml) stably expressing Lifeact-iRFP670 (F-actin) and TetOn3G (positivity visible as cytoplasmic TagBFP). Scale bar 20 μm. **C)** Cells also transfected with GFP. Arrows, colocalization of mCherry/GFP at perinuclear recycling compartment. **E)** Violin plot shows the quantification of cell length (shown in D) measured as maximum ferret diameter based on Lifeact-iRFP670 (F-actin). Dots, median for each independent experiment. n>74 cells ± dox from, N=3. Two-tailed Mann–Whitney U test (**P<0.01). **F)** Live imaging of cells on FN, dotted line shows orientation of kymograph. Arrow, positive correlation between trafficking of Rab25 endosomes towards the plasma membrane and actin polymerisation/protrusion. **G)** Live imaging of cells in 3D CDM. Dotted line shows orientation of kymograph. Arrow, positive correlation between trafficking of Rab25 endosomes towards the plasma membrane and actin polymerisation/protrusion. **H, I)** Correlation between the movement of Rab25 endosomes and the tracks of actin polymerisation events. **H)** Live imaging of cells on FN, dotted line shows orientation of kymograph. Boxed area, zoom inset showing timelapse frames. Arrow, colocalizing fluorescent signal of NB^{GFP}-mCherry-Rab25 endosomes (R25) and actin tracks visualized by LifeAct-iRFP670 (F-actin). **I)** Live imaging of cells on FN-treated 4 h with 1 μM Nocodazole**.** Dashed line, position of Rab25 endosome (R25) at the time 0. Colour-grade (cyan-red) LUT image show Rab25 endosome movement (R25) and F-actin fiber polymerisation in time not blocked by depolymerisation of microtubules. Arrow, colocalizing fluorescent signal of NB^{GFP}-mCherry-Rab25 endosomes (R25) and actin tracks.

fig. S2

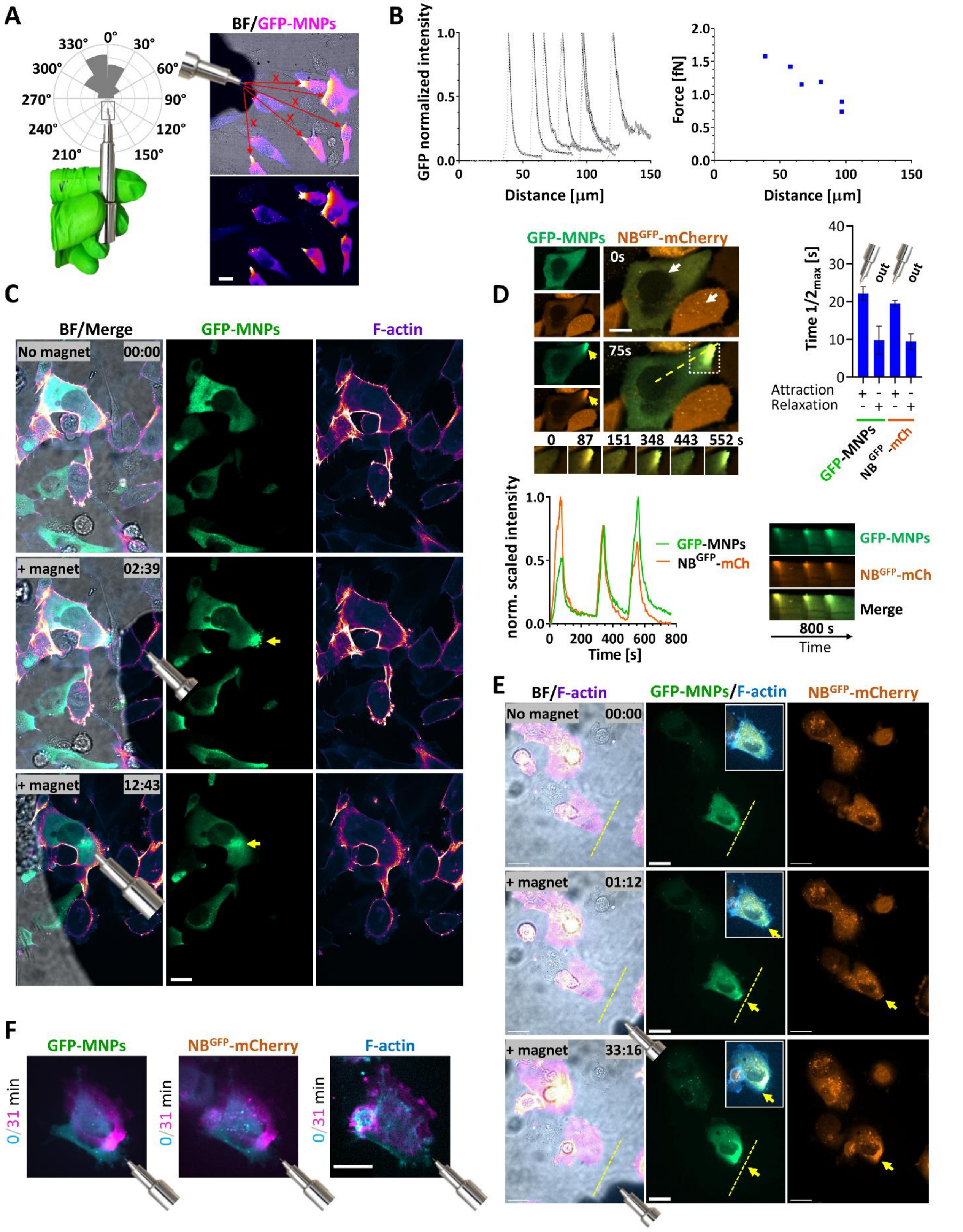

Figure S2. Magnetic manipulation of GFP-MNPs and NB^{GFP}-mCherry inside living cells. A-F) Images generated by confocal spinning-disk microscopy (live cells). GFP-MNPs delivered by microinjection to cells on FN-coated coverslips. Brightfield (BF). **A-B)** Steady-state GFP-MNPs gradient profiles inside the cytoplasm of living A2780 cells generated by a magnetic gradient. **A)** Confocal spinning-disk live cell images of A2780 and angle between micro-magnet and centre of the microinjected cells with observed attraction gradient, N = 3 (n = 40). Scale bar 20 μm. **B)** The decay into the cytoplasm was fitted by an exponential decay to estimate the force exerted on the GFP-MNPs (see Methods,

Figure S2. (Continued from previous page.) Equation 1) and plotted as function of magnetic tip distance from cells. **C)** Key time-lapse image frames showing magnetic manipulation of GFP-MNPs inside living A2780 cells stably expressing Lifeact-iRFP670 (F-actin). Shadow in brightfield indicates magnetic tip (exact position indicated by cartoon). Arrow, GFP-MNPs enrichment **(**see movie S1). Scale bar 20 µm. **D-E)** A2780 cells stably expressing NB^{GFP}-mCherry (amber), Lifeact-iRFP670 (F-actin, blue or gem LUT) microinjected with GFP-MNPS (green). Movies S11-S13 (S12 shows magnetic manipulation across whole cells) accessible via https://doi.org/10.6084/m9.figshare.22155083. **D)** Magnetic attraction and release kinetics of NB^{GFP}-mCherry-(X) and GFP-MNPs in living cells; individual representative frames (scale bar 10 µm) from highlighted white box, kymographs from dashed line and profiles (bottom left) determined from the changes in fluorescence intensity across kymograph (black arrow; normalized 0-1 scaled fluorescent intensities shown), exponentially fitted and attraction and relaxation kinetics quantified (right) as time needed to reach 50 % of maximal gradient of the steady-state (max; top right); all conditions n=3 (N=3). Out: no magnet. Arrows, accumulation of NB^{GFP}-mCherry in the cytoplasm (out from nucleus) in GFP-MNPs positive cells. **E-F)** Representative example of ctrl migrating cell with magnetically attracted GFP-MNPs/ NB^{GFP}-mCherry, arrow. Magnetic tip indicated by shadow and/or cartoon. Dashed line, cell edge (F-actin) at the time 0. Inset, merge GFP-MNPs/ NB^{GFP}-mCherry/F-actin. Scale bar 20 µm. **F)** MIPs used for scoring protrusion growth (F-actin) shown in Fig. 1D.

fig. S3

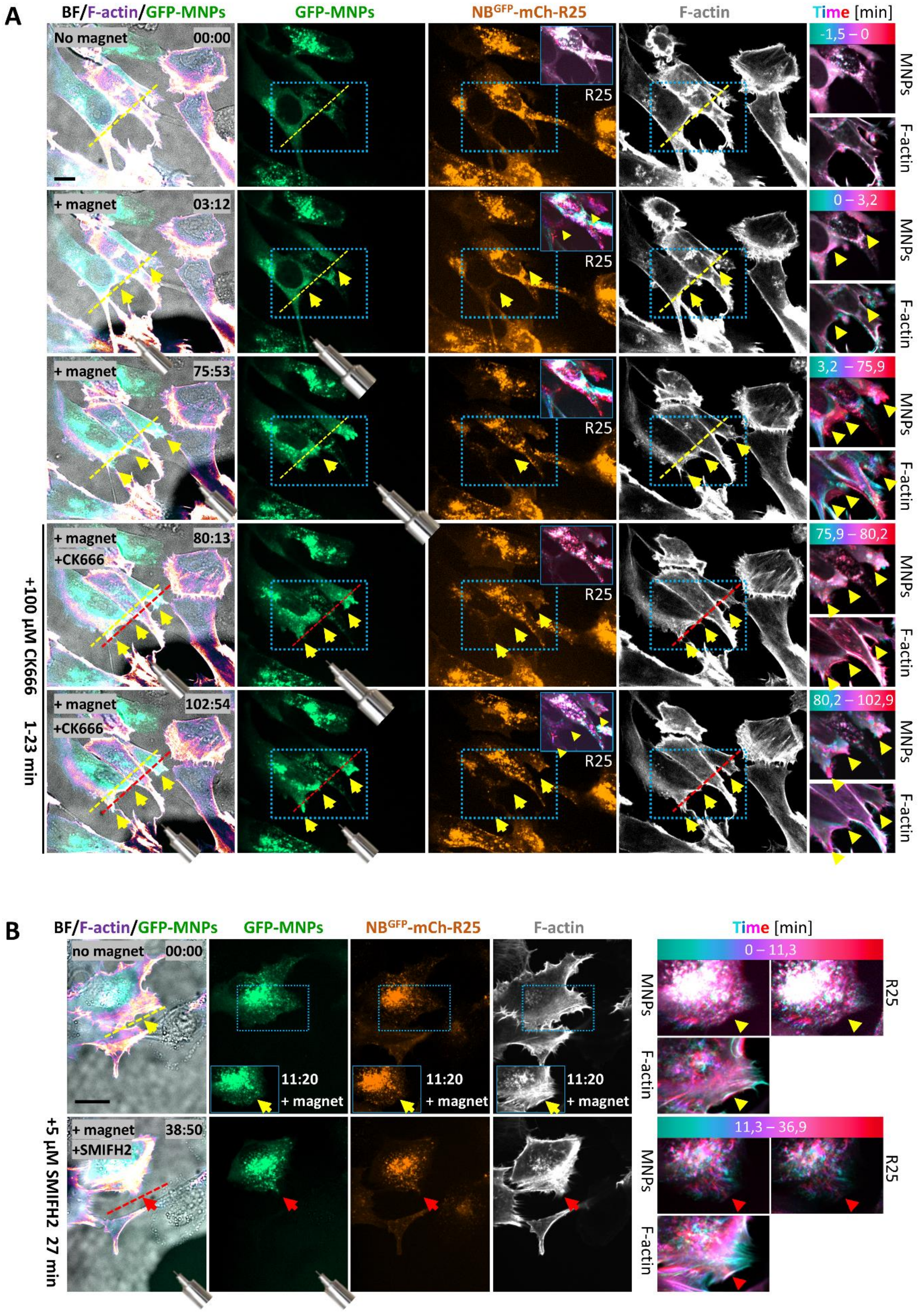

Figure S3. Remote manipulation of Rab25 endosomes controls protrusion outgrowth in an Arp2/3 independent and formin-dependent manner. A, B) Representative spinning-disk confocal live cell imaging, A2780 DExCon-modified NB^{GFP}-mCherry-Rab25 cells dox pre-treated (>94 h; 250 ng/ml) stably expressing Lifeact-iRFP670 (F-actin), microinjected with GFP-MNPs. The effect of Rab25 endosomes remotely redistributed to promote protrusions is not inhibited by **A)** CK666 treatment (100 µM, movie S14 accessible via https://doi.org/10.6084/m9.figshare.22155083, but

Figure S3. (Continued from previous page.) blocked with **B)** SMIFH2 (5 uM; movie S15 accessible via https://doi.org/10.6084/m9.figshare.22155083). Shadow in brightfield (BF) and cartoon indicates position of the magnetic tip. Yellow arrow, changes in GFP-MNPS/ NB^{GFP}-mCherry fluorescent signal or protrusion growth (F-actin). Red arrow, protrusion retraction/no response (F-actin). Boxed area, cyan-red LUT illustrates changes in GFP-MNPs and vesicle distribution (R25) and protrusion changes (F-actin) over time through colour grading. Yellow dashed line, cell edge (F-actin) at the time 0. Red dashed line, cell edge (F-actin) when added inhibitor. FN-coated coverslips, Scale bar 20 μm.

fig. S4

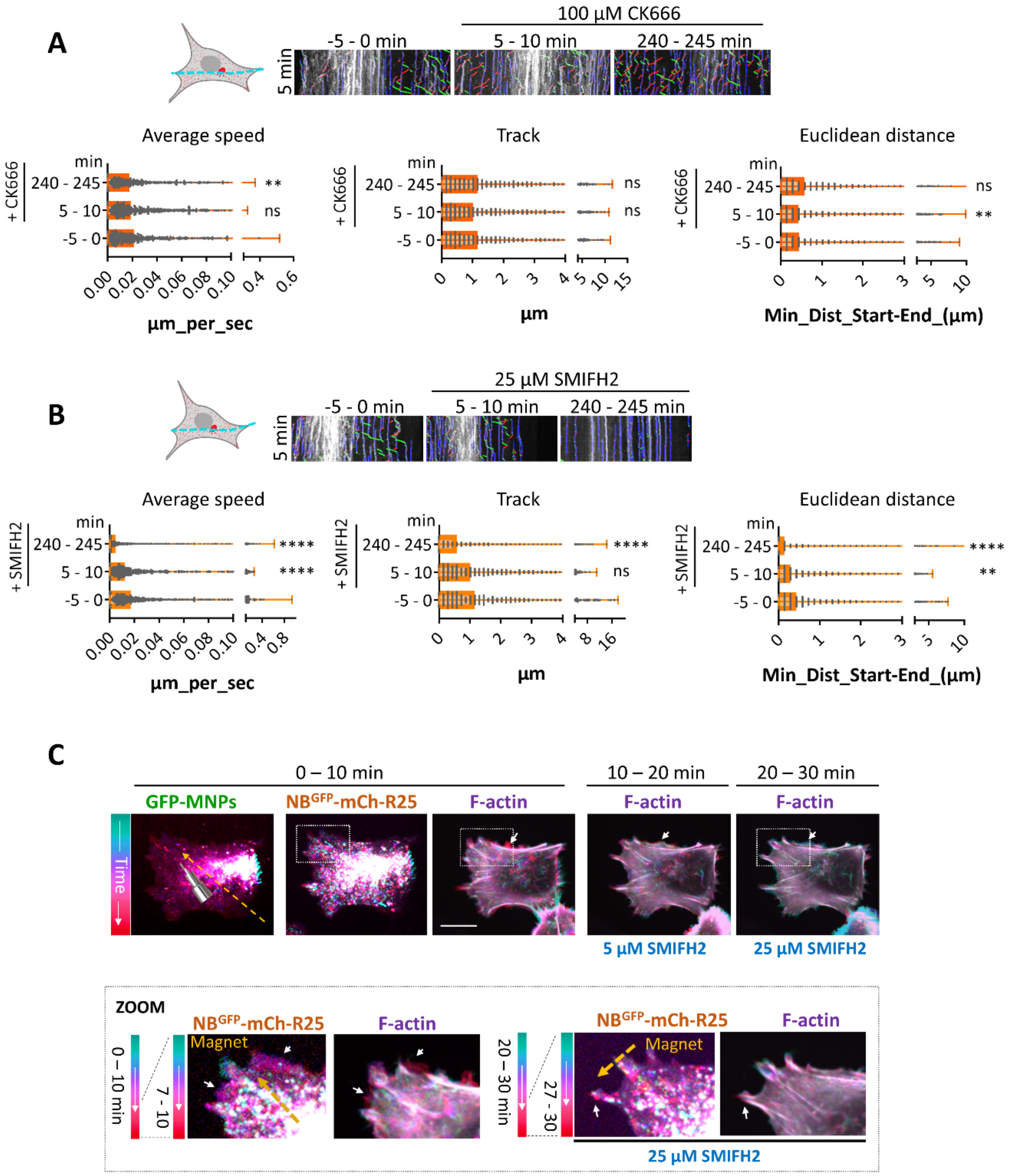

Figure S4. Active movement of Rab25 endosomes in A2780 cells requires formins, but not Arp2/3.
A, B, C) Confocal spinning-disk live cell imaging of A2780 DExCon-modified NB^{GFP}-mCherry-Rab25 (R25; dox treated > 48 h, 250 ng/ml) stably expressing Lifeact-iRFP670 (F-actin) on FN. **A, B)** AI based tracking of vesicles (NB^{GFP}-mCh-R25) movement (labelled blue, static; brown, towards rear; green, towards front) using kymographs (generated from dashed line, schematic cartoon) and Kymobutler plugin before and after treatments: **A)** CK666 (100 µM) or **B)** SMIFH2 (25 µM) as indicated. Representative kymographs are shown, -5 – 0 min/ 5-10 min same cell; 240-245 min different cell. Average speed, vesicle total track or euclidean distance quantified; *n*>9 cells/condition (N=3); Anova on ranks, Dunn's test (compared to no treatment). ***P*<0.01; ****P*<0.001; *****P*<0.001. **C)** Magnetic relocalization of GFP-MNPs and Rab25 endosomes (NB^{GFP}-mCh-R25) towards upper part of the cell, cells treated by SMIFH2 as indicated and position/movement of magnet (cartoon) indicated by orange arrows. Boxed area, cyan-red LUT illustrates changes in GFP-MNPs and vesicle distribution (R25) and protrusion changes (F-actin) over time through colour grading. Representative cyan-red LUT images of F-actin show actin-dependent protrusion dynamics before and after SMIFH2 treatment (N=3). Boxed area, zoom inset bellow. White arrow, protrusion and vesicle movement highlighted. FN-coated cover-slip. Scale bar 20 µm.

fig. S5

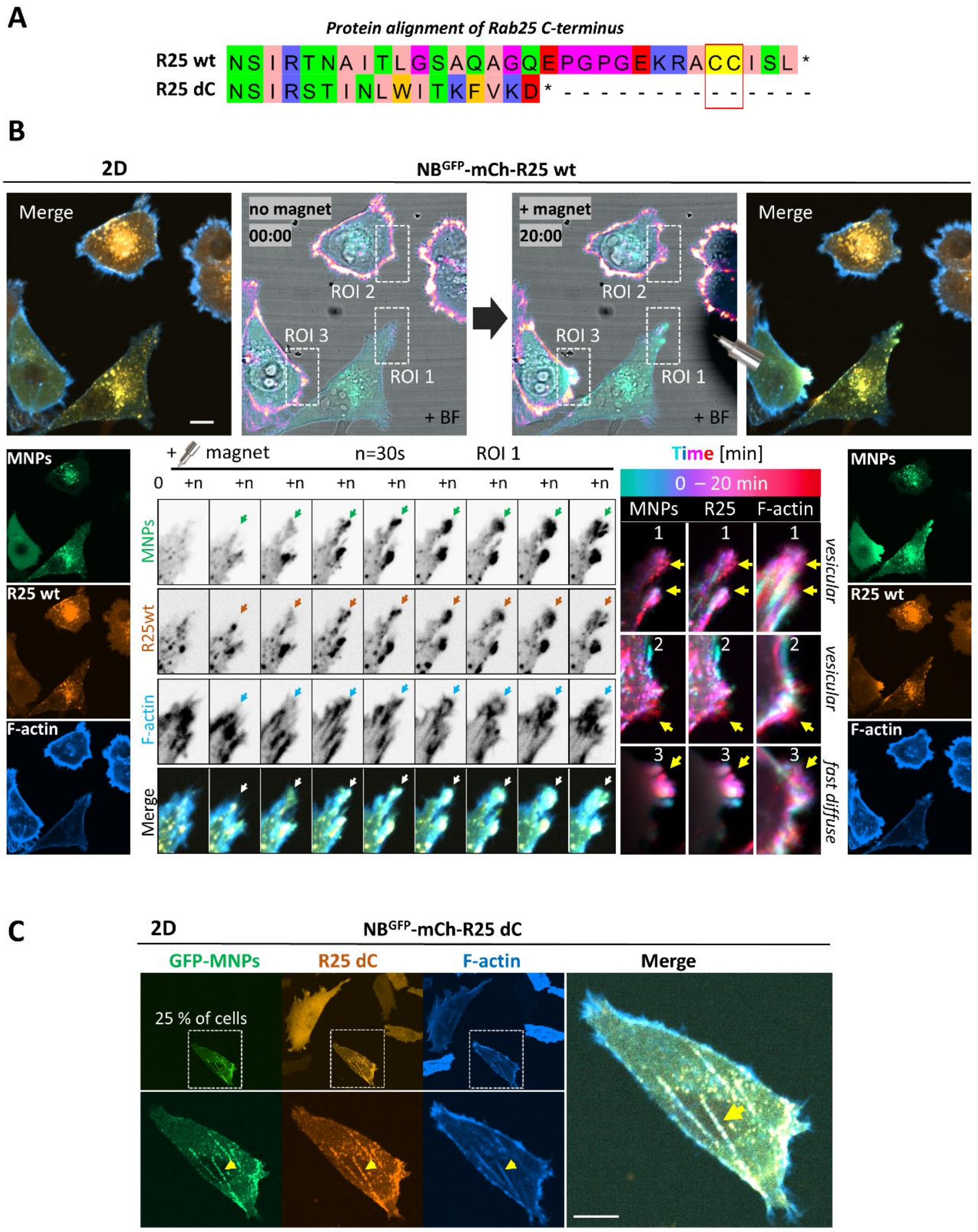

Figure S5. Rab25 promotes actin-dependent protrusion growth. A) Protein alignment of C-terminus of Rab25 wt and dC mutant. Zappo color coding in Jalview. Red rectangle, membrane attachment site (CC). **B)** Magnetic attraction of Rab25 (R25) endosomes promotes protrusion growth independently of initial cell polarity. Confocal spinning-disk live cell timelapse imaging of A2780 cells stably expressing NB^{GFP}-mCherry-Rab25 wt (biop LUT amber) with Lifeact-iRFP670 (F-actin, biop LUT azure) on FN, microinjected with GFP-MNPs (biop LUT spring green). Magnetic tip visible as shadow in brightfield (BF) and indicated by cartoon. Boxed area, Region of interest (ROI) 1-3 shown as cyan-red LUT illustrating changes in GFP-MNPs and vesicle distribution (R25) and protrusion changes (F-actin) over time through colour grading. ROI 1 also shown as individual time frames. Arrows, correlation of changes in individual channels. Movie S17 accessible via https://doi.org/10.6084/m9.figshare.22155083. Scale bar 10 µm. **C)** Confocal spinning-disk live cell imaging of A2780 cells stably expressing NB^{GFP}-mCherry-Rab25 dC (biop LUT amber) with Lifeact-iRFP670 (F-actin, biop LUT azure) on FN, microinjected with GFP-MNPs (biop LUT spring green). Representative example of ~25 % cells with Rab25 dC signal co-aligned with F-actin stress fibers induced by GFP-MNPs microinjection, arrow (not visible in Ctrl NB^{GFP}-mCherry expressing cells). Scale bar 10 µm.

fig. S6

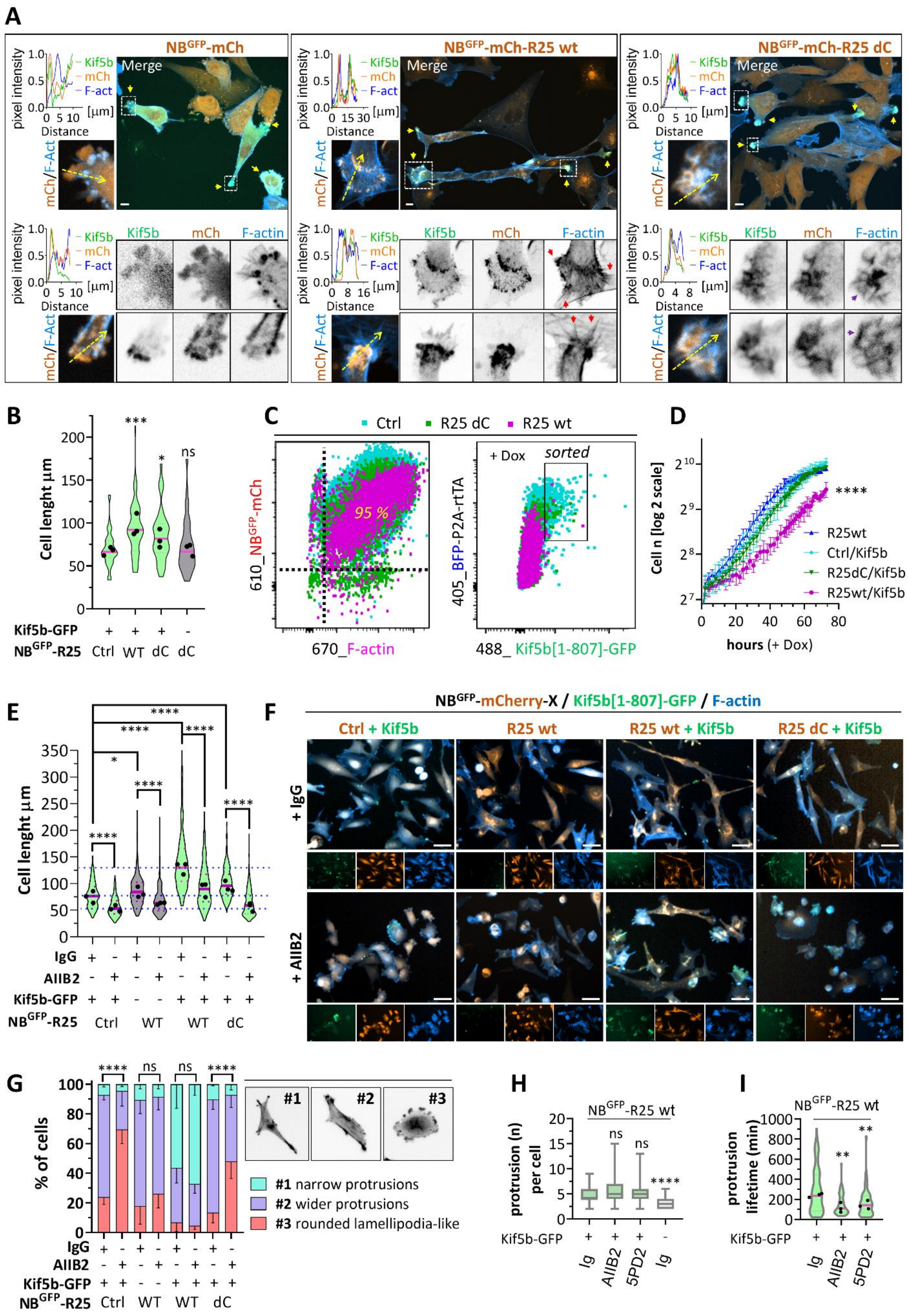

Figure S6. Rab25 targeted to cell periphery via Kif5b locally stimulates actin polymerisation. A) Confocal spinning-disk live cell imaging of A2780 stably expressing NB^{GFP}-mCherry (Ctrl, left) or fused with Rab25 wt (middle) or

Figure S6. (Continued from previous page.) Rab25 dC (right) with Lifeact-iRFP670 (F-actin) prepared by lentiviral transduction (sorted for matched expression levels) and truncated GFP-fused Kif5b [1-807] generated by sleeping Beauty system constitutively expressing BFP, rtTA and puromycin resistance gene ((*68*); selected by puromycin). All dox treated (48 h; 500 ng/ml). Merge, MIPs. Boxed area, zoom inset shown as one Z plane, two examples for each variant shown. Dotted line, line scan profile of normalized 0-1 scaled fluorescence intensities. Yellow arrows, Kif5b positive protrusions. Red arrows, filopodia. Purple arrows, lamellipodia-based structures. FN-coated μ-Plate 96 plate (#1.5 IbiTreat). Scale bar 10 μm. Cell length quantified in **B)** based on F-actin as maximum ferret diameter. Only cells co-expressing BFP/GFP (readout of Kif5b positivity) were analysed (except Kif5b negative Rab25 dC cells). One-way ANOVA analysis Dunnet post hoc test (compared to Ctrl). Dots, median for each independent experiment. n>42 cells all conditions; N=3. **P*<0.05; ****P*<0.001. **C)** Cells described in A) sorted for matched GFP-KIF5b expression levels as indicated. **D)** Representative example of proliferation rate of cells described in C) analysed by eSight real-time proliferation assay; N=4 (all dox treated; 500 ng/ml). Cell numbers (n) derived from brightfield trained object masks, normalized for matched numbers at the time 0. The linear part of the cell proliferation rate (10-50 h) plotted by log2 was fitted by simple linear regression and compared using an Analysis of Covariance (ANCOVA). *****P*<0.001 (between Rab25 wt ± Kif5b). **E-I)** Widefield live cell imaging of sorted cells described in C). Integrin β1 blocking antibodies added 24 h after seeding cells into FN-coated 96 well plate (cellvis, #1.5H cover glass) and imaged starting 24 h thereafter. AIIB2 10 μg/ml; 5PD2 10 ug/ml. IgG (10 μg/ml), control treatment. All dox treated (48-72 h; 500 ng/ml). Representative images shown in **F)** as merge or individual fluorescent channels (GFP, mCherry, iRFP670). Scale bar 50 μm. Cell length quantified in **E)** based on F-actin as maximum ferret diameter (24h AII/IgG treatment; dividing cells or cells not able to spread are not included in the analysis). Dots, median for each independent experiment. n>74 cells ± dox from, N=3. Anova on ranks, Dunn's test (compared altogether or to Ctrl in IgG treated condition). **G)** Quantification of cell protrusion and morphology where 1= narrow protrusion < 5μm width; 2= wide protrusions > 5μm width, and 3= rounded cells exhibiting broad lamellipodia/ruffles (dividing cells or cells not able to spread are not included in the analysis). n>165 cells; N=3. Two-way ANOVA analysis Tukey post hoc test (cross-correlation comparison of the similarity of treatment-induced changes). The number (n) of narrow protrusions (width ≤ 5 μm; specified in fig. 2G) per cell and their lifetime is quantified in **H)** n≧70 cells (N=3) and **I)** n>60 cells (N=3), respectively. Anova on ranks, Dunn's test test (compared to IgG treated Kif5b positive cells). All graphs *P<0.05; **P<0.01; ***P<0.001; ****P<0.001.

fig. S7

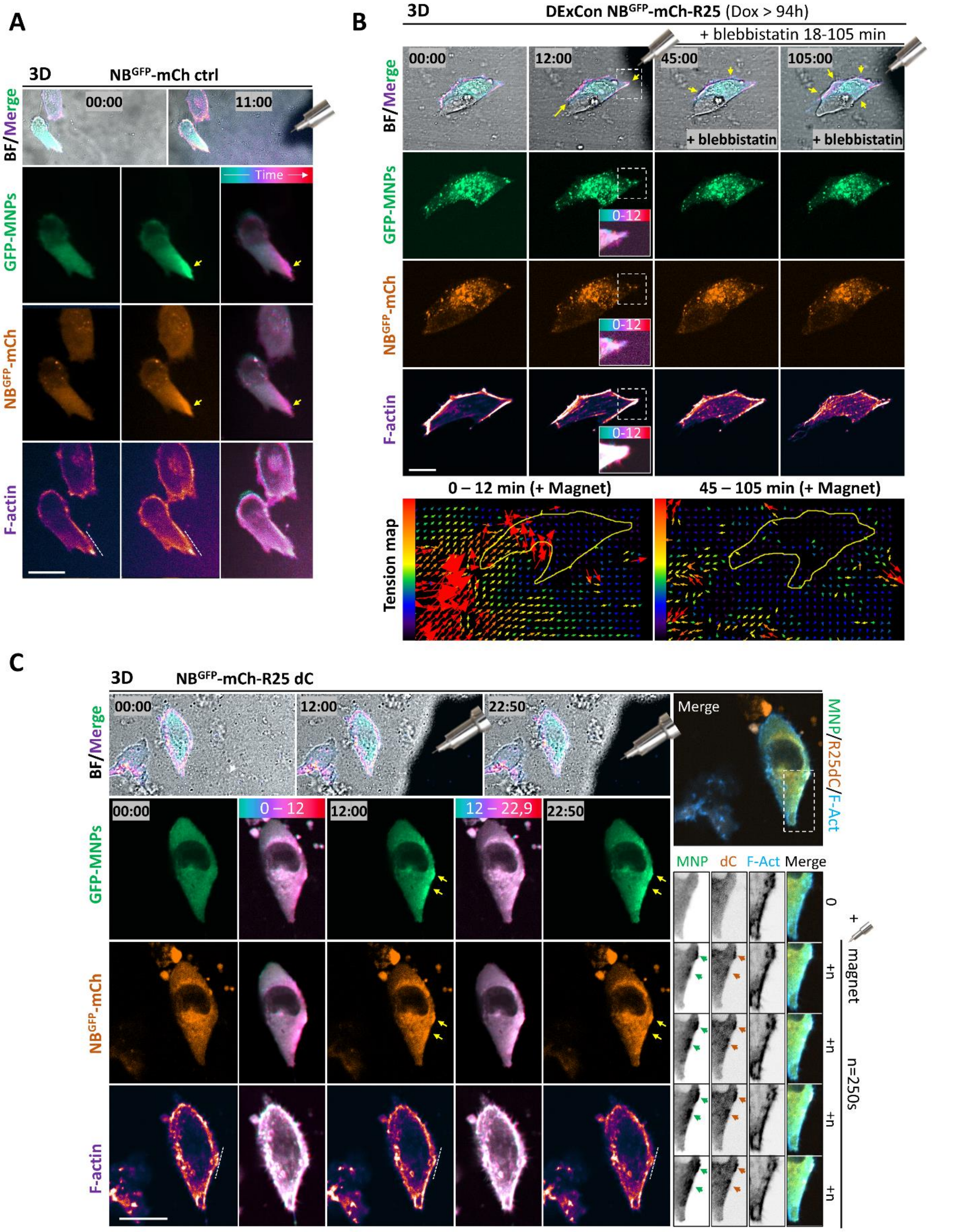

Figure S7. Rab25's ability to interact with vesicles enables the mechanosensing of magnetic forces by MNP-bound Rab25 endosomes and is essential to promote protrusion growth in CDM. A-C) Representative images from confocal spinning-disk live cell imaging of cells in 3D CDM; Scale bar 20 μm; GFP-MNPs delivered by microinjection and position of magnetic tip visible as shadow in brightfield (BF) or indicated by cartoon. The cyan-red LUT illustrates changes in NB^{GFP}-mCherry-X/GFP-MNPs distribution and cell shape (F-actin) over time through colour grading. Representative example; N = 3 (4 for Rab25 dC shown in C). **A)** A2780 stably expressing NB^{GFP}-mCherry A2780 with Lifeact-iRFP670 (F-actin). Dashed line, cell edge (F-actin) at the time 0. Arrow, gradient of fluorescent signal. **B)** Confocal spinning-disk live cell imaging of A2780 DExCon-modified NB^{GFP}-mCherry-Rab25

Figure S7. (Continued from previous page.) (R25; dox treated > 94 h, 250 ng/ml) stably expressing Lifeact-iRFP670 (F-actin). Cell adaptation to mechanosensing of magnetic force by MNP-bound Rab25 endosomes (time 0-12 min) is perturbed by blebbistatin (5 μM) treatment (18-105 min), no magnet in between (30 min blebbistatin pre-incubation). Arrows, cell shape changes (shrinking/growth 0-12 min or swelling 45-105 min). Relative stress maps are displayed using as a vectorial plot with red-blue LUT, size and direction of vectorial arrows illustrate force distribution exterted on CDM. **C)** A2780 cells stably co-expressing NB^{GFP}-mCherry-Rab25 dC and Lifeact-iRFP670 (F-actin). Dashed line, cell edge (F-actin) at the time 0. Arrow, gradient of fluorescent signal. Movies S18-20 accessible via https://doi.org/10.6084/m9.figshare.22155083.

fig. S8

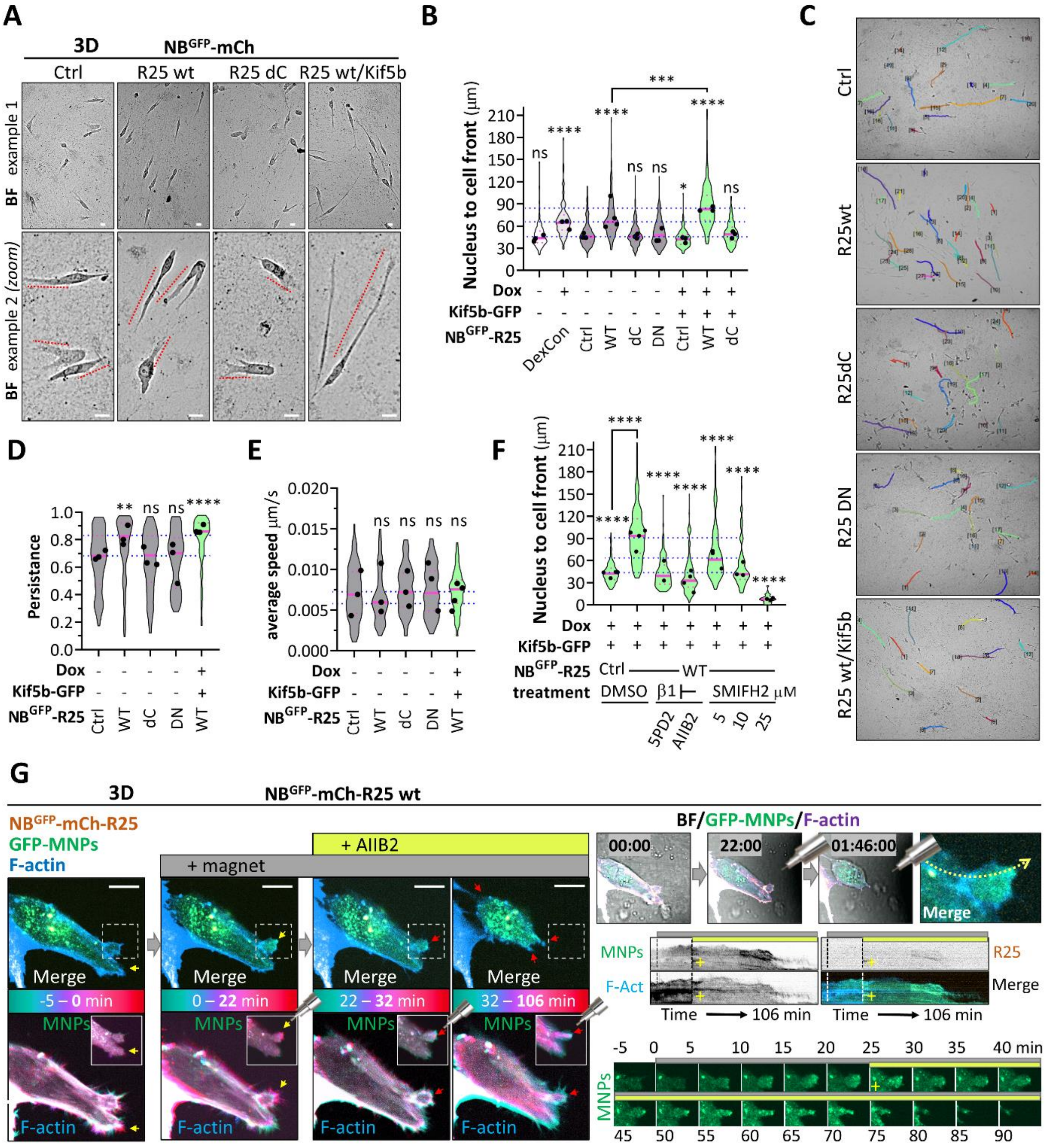

Figure S8. Rab25's ability to interact with integrin β1-containing recycling vesicles is essential to promote protrusion growth in CDM. A-F) Cell migration in 3D CDM; A2780 DExCon-modified NB^{GFP}-mCherry-Rab25 (± dox treated 48 h, 250 ng/ml) or A2780 cells stably co-expressing Lifeact-iRFP670 (F-actin) with NB^{GFP}-mCherry (Ctrl) or fused with Rab25 wt or Rab dC and truncated GFP-fused Kif5b [1-807] sorted for matched GFP-KIF5b expression levels (dox treated 24-48 h, 500 ng/ml) shown in fig. S6C. Dots, median for each independent experiment. **A)** Widefield live cell imaging (brightfield (BF)). CDM (in for 8-24h). Zoom inset from diff. area. Scale bar 10 µm. Dotted line, length of pseudopodial protrusion (nucleus to the cell front of migrating cells) quantified in **B)** and shown as violin plot; n=150-300 cells/condition, N=3-4. **C)** Representative example of cell tracks, quantification of migration persistence **D)** and average speed **E)**; cells in CDM for 8-24h; n>70, N=3. **F)** Quantification of length of pseudopodial protrusion (nucleus to the cell front of migrating cells) of cells described in A) ± treatment shown as violin plot. Cells in CDM for 8-24h; n > 150 cells/condition (50 for 25uM SMIFH2 as majority of cells are immobile); N=3 if not stated otherwise (vehicle or no treatment N=4); SMIFH2 added 4h after spreading (µg/ml as indicated); Integrin β1 blocking antibodies added before seeding cells into CDM: AIIB2 10 µg/ml (N=3); 5PD2 10-15 ug/ml (N=2). All graphs Anova on ranks, Dunn's test (compared to ctrl or Rab wt/Kif5b as indicated); **P*<0.05; ***P*<0.01; ****P*<0.001; *****P*<0.001. **G).** Representative images from confocal spinning-disk timelapse images of A2780 NB^{GFP}-mCherry-Rab25 wt cell expressing Lifeact-iRFP670 (F-actin), migrating in CDM (3D), microinjected with GFP-MNPs before (-5-0 min) or after GFP-MNPs

Figure S8. (Continued from previous page.) magnetically attracted towards magnetic tip (0-106 min) visible as shadow in brightfield (BF); see cartoon). Rab25 promoted protrusion growth (0-22 min) is blocked by integrin β1 blocking antibody (+; yellow rectangle) treatment (AIIB2, 10 μg/ml; 22-32 min) followed by protrusion retraction/cell rounding (32-106 min) despite sustained GFP-MNPs magnetic attraction (grey rectangle, see timelapse frames and kymographs). The cyan-red LUT illustrates changes in protrusion growth (F-actin) over time through colour grading (GFP-MNPs inset; cropped cell front). Yellow arrow, protrusion growth. Red arrow, protrusion no change/retraction. Boxed area, individual representative time frames. Dotted line, kymograph. N=3. Scale bar 10 μm. See movie S5.

fig. S9

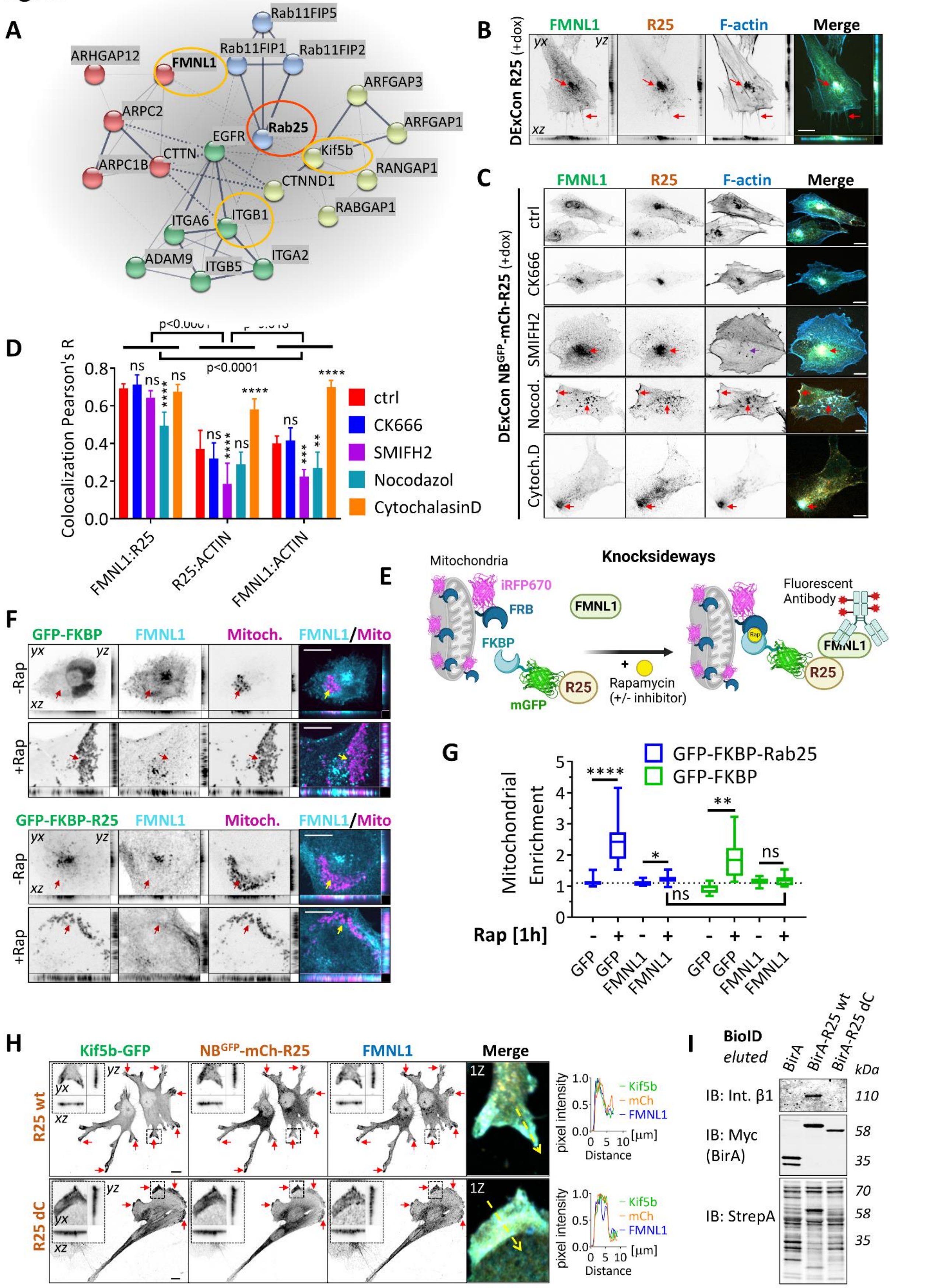

Figure S9. The interaction between Rab25 and FMNL1 is of a transient nature, but FMNL1 and integrin β1 are cargoes of Rab25 vesicles. A) Rab25-BioID (proximity labelling) dataset (*22*) re-analysis (see methods). Rab25-BirA (red circle) enriched cell matrix interactors (green); Actin/GTPase regulators (red and white), Rab effectors (blue) are represented using STRING protein-protein interaction analysis **(**https://string-db.org/**).** Interesting targets are

Figure S9. (Continued from previous page.) circled in yellow (ITGB1/integrin β1; FMNL1; KIF5b). **B-C)** Representative confocal spinning-disk images of A2780 DExCon-modified NB^{GFP}-mCherry-Rab25 cells (mCherry) on FN-pre-treated with dox (> 94 h; 250 ng/ml) immunolabeled for FMNL1 (rabbit anti-FMNL1; Alexa-488) and stained for F-actin (Phalloidin-Alexa633). Scale bar=10 µm. **B)** MIP with cross-sections. Arrows, filopodia or perinuclear recycling compartment. **C)** MIPs. Ctrl (no treatment); CK666 (100 µM; 4h); SMIFH2 (25 µM; 4h); Nocodazol (1 µM; 4h); Cytochalasin D (100 ng/ml (200 nM); 4h). Red arrows, Enriched colocalized FMNL1/Rab25/F-actin signal (purple arrow; missing F-actin signal upon SMIFH2 treatment). FMNL1/Rab25/F-actin signal colocalization in **D)** using Pearson's R. Two-way ANOVA analysis Tukey post hoc test (compared with ctrl or cross-correlation of the similarity of treatment-induced changes between FMNL1:R25, R25:ACTIN, and FMNL1:ACTIN, as indicated); $^{**}P<0.01$; $^{***}P<0.001$; $^{****}P<0.001$. **E)** Schematic diagram (created with BioRender.com, https://BioRender.com/lb9y1fd) of knock-sideways experiment whose representative images are shown in **F)** and quantified in **G)** as colocalization of GFP signal or proportional co-enrichment of FMNL1 in mitochondria (mask from mitochondrial targeting sequence (Mito) fused with iRFP670-FRB). A2780 co-transfected with Mito-iRFP670-FRB (iRFP670) and FKBP-GFP-Rab25 wt (GFP), ± Rapamycin (Rap; 1h, 200 nM), fixed and immunolabeled for FMNL1 (rabbit anti-FMNL1; cy3). MIP with cross-section is shown. Arrows, mitochondria based on Mito-iRFP670-FRB (iRFP670) channel. Scale bar 10 µm. **G)** One-way ANOVA analysis Tukey post hoc test (compared between GFP; GFP-Rab25 ± rap or between FMNL1 ± rap); $^{*}P<0.05$; $^{**}P<0.01$; $^{****}P<0.001$. **H)** Representative confocal spinning-disk images of A2780 cells stably co-expressing NB^{GFP}-mCherry-Rab25 wt or Rab dC and truncated GFP-fused Kif5b [1-807] sorted for matched GFP-KIF5b expression levels (dox treated 24-48 h, 500 ng/ml) immunolabeled for mCherry (rat anti-RFP; Alexa-555); GFP (mouse anti-GFP; Alexa-488), and FMNL1 (rabbit anti-FMNL1; Alexa-633). MIPs. Boxed area, zoom inset (MIP) shown with cross-sections or shown as merge (1Z). Dashed line, line scan profile of normalized 0-1 scaled fluorescent intensities from 1Z plane. Arrows, narrow protrusions (width ≤ 5 µm) with vesicular FMNL1 (Rab25 wt) or lamellipodia-like (width > 5 µm; Rab25 dC). Scale bar 10 µm. **I)** A2780 stably expressing BirA or BirA fused with Rab25 WT or Rab25 dC mutant cultured with biotin (1 µM biotin, 16 h). Lysates equalized to total protein amount and biotinylated proteins pulled down with Streptavidin beads. Immunoblots of pulled down integrin β1, BirA (Myc epitope), Streptavidin (StrepA) as loading control. Fluorescent antibodies shown as black and white.

fig. S10

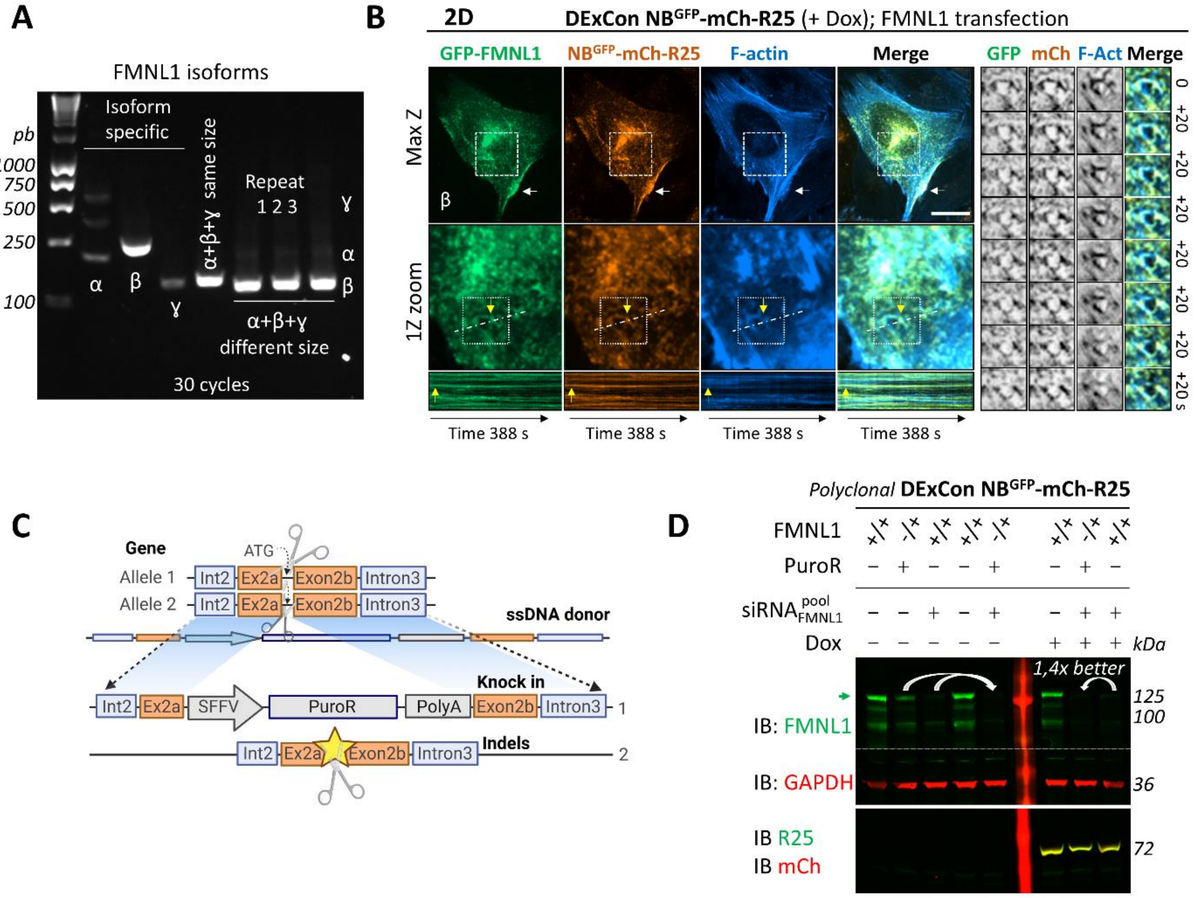

Figure S10. Rab25 endosomes predominantly functionally associates with FMNL1 β isoform and strategies how to efficiently deplete FMNL1 in A2780 cells. A) Semiquantitative PCR (30 cycles). Agarose gel, PCR products of FMNL1 isoforms (α, β, γ) amplified using combination of non-distinguishing, isoform specific or semi-specific primers leading to different sizes of PCR products of FMNL1 isoforms (3 independent biological repeats shown). **B)** Representative confocal spinning-disk live cell imaging of A2780 DExCon-modified NB^{GFP}-mCherry-Rab25 (R25; dox treated 48 h, 250 ng/ml) stably expressing Lifeact-iRFP670 (F-actin) transfected by GFP fused FMNL1 β isoform. MIP or 1Z plane shown as indicated. Boxed dashed area, zoomed inset. Solid boxed area, individual timelapse frames (n + 20s). Dashed line, kymograph. White arrow, FMNL1/Rab25/F-actin colocalizing signal in protrusion. Yellow arrow, FMNL1/Rab25/F-actin stable endosomal colocalization. Scale bar 20 µm. **C)** Strategy to enrich pool of DExCon-modified A2780 cells with inactivated FMNL1 gene by indel formation and homologous recombination of cassette carrying SFFV promoter with puromycin resistance into exon2 of FMNL1 (see also Fig. 5A; for additional details Methods and extended fig. S10C-H). Created with BioRender.com (https://BioRender.com/nsezjwg). **D)** Comparison of gene editing (described in C), siRNA knockdown and siRNA/CRISPR combined strategy (arrows). Immunoblot of DExCon-modified NB^{GFP}-mCherry-Rab25 cells FMNL1+/+ or FMNL1 PuroR/+ (-/+) nucleofected (5 days after) with siRNA pool (s26, s27, s28) anti-FMNL1, ± dox (48 h, 250 ng/ml) as indicated. Overlay of FMNL1 and GAPDH (loading control) or Rab25 (R25) and mCherry (mCH), respectively. fluorescent antibodies.

fig. S11

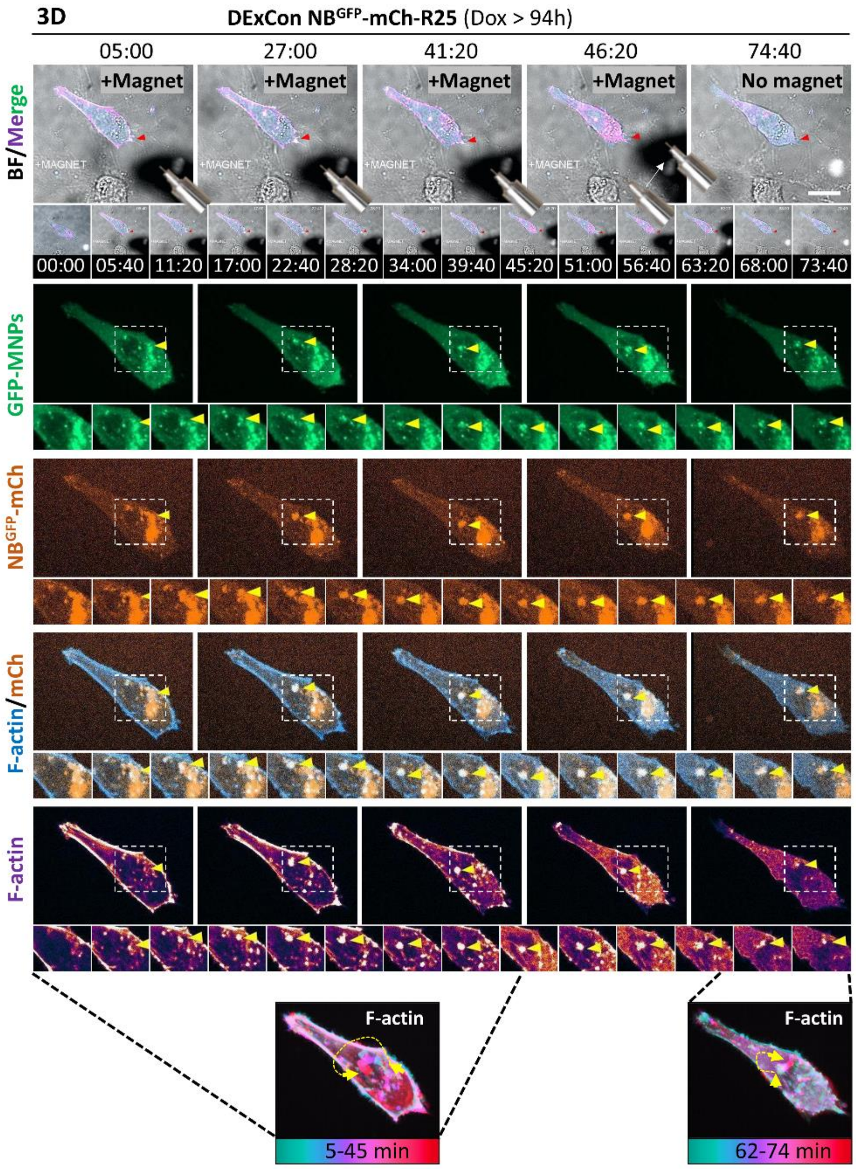

Figure S11. Magnetic relocalization of endosomal cluster via Rab25 locally induce actin polymerisation hot-spot. Representative images from confocal spinning-disk live cell imaging. A2780 DExCon-modified NB^{GFP}-mCherry-Rab25 cells dox pre-treated (72 h; 250 ng/ml) expressing Lifeact-iRFP670 (F-actin) migrating in CDM (3D). GFP-MNPs delivered by microinjection. Magnetic attraction of GFP-MNPs/NB^{GFP}-mCherry-Rab25 endosomal cluster towards magnetic tip (0-46 min) visible as shadow in brightfield (BF); see cartoon) promotes formation of co-moving actin polymerisation hotspot (arrow; F-actin shown as cyan-red LUT 5-45 min) followed by reverse movement and intensity decrease upon magnet removal (arrow; F-actin shown as cyan-red LUT 62-74 min). Boxed area, individual timelapse frames (n + 5 min 40s). Scale bar 20 μm. Movie S21 accessible via https://doi.org/10.6084/m9.figshare.22155083.

fig. S12

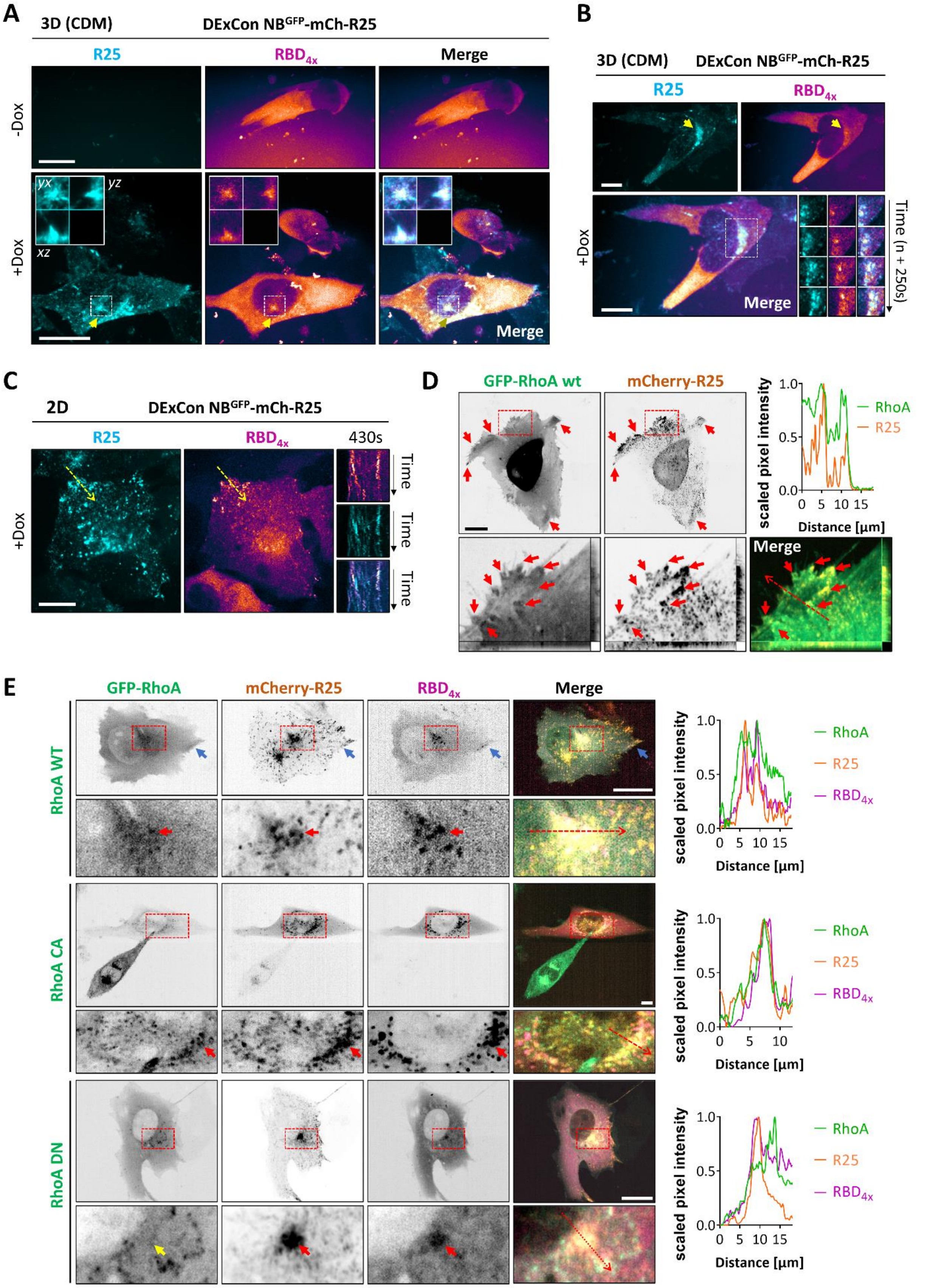

Figure S12. Specificity of iRFP670(3x)-RBD(4x) sensor to report RhoA activity and its colocalization with Rab25. **A-E)** Representative images from confocal spinning-disk live cell imaging. Scale bar 20 µm. **A-C)** A2780 DExCon-modified NB^{GFP}-mCherry-Rab25 (R25; dox treated ≥ 48 h, 250 ng/ml) stably expressing active RhoA probe iRFP670$_{3x}$-RBD$_{4x}$ (RBD$_{4x}$). **A, B)** 3D CDM. MIPs. Boxed area, zoom inset with cross-sections A) or timelapse frames

Figure S12. (Continued from previous page.) (from 1Z plane; B). Arrow, PNRC. **C-E)** FN-coated μ-Plate 96 plate (#1.5 IbiTreat). **C)** Line, kymograph area, zoom inset with cross-sections. Arrow, PNRC. **D)** A2780 cells co-transfected by GFP-RhoA wt and mCherry-Rab25 wt. MIPs. Boxed area, zoomed inset with cross-sections. Arrows, filopodia based protrusions. **E)** A2780 cells co-transfected by mCherry-Rab25 wt (R25), iRFP670$_{3x}$-RBD$_{4x}$ (RBD$_{4x}$) and GFP fused RhoA wt or mutants (DN = dominant negative; T19N, CA = constitutively active; Q63L). MIPs. Boxed area, zoom inset. Blue arrow, RhoA/R25/RBD$_{4x}$ signal at protrusion. Red arrow, RhoA/R25/BD$_{4x}$ signal enriched at PNRC/endosomes (RhoA WT or CA, but missing from DN, yellow arrow). **D-E)** Red dashed line, line scan profile of normalized 0-1 scaled fluorescence intensities.

Supplementary information

Plasmid maps with sequences generated in this study can be download from https://doi.org/10.6084/m9.figshare.27084205. List of material and other sequences are provided in Table S1-S5 or accessible from https://doi.org/10.6084/m9.figshare.27084217. Plasmids can be provided upon reasonable request from P.C. (patrick.caswell@manchester.ac.uk) or J.G. (jakub.gemperle@img.cas.cz). All representative full-resolution S1-S10 movies (Full HD) have been deposited in the Figshare repository and can be accessed via from https://doi.org/10.6084/m9.figshare.22155083, as can the additional movies S11-S22 which could not be incorporated directly with this study. Full description of included movies is in the accompanying movie legend.

Table S1-S5 with list of primers used for ssDNA preparation, screening of knock in outcomes and semi-quantitative PCR, synthetized DNA sequences, crRNA sequences and list of all plasmids, regencies and antibodies used in this study.

Table S1. List of plasmids

Name	Source	Identifier
CRISPR/Homologous recombin. donor		
pJET-24_Rab25 DExCon donor (TRE3GS_NB_{GFP}-mCherry)	Gemperle et al., 2022	NA
pJET-78-FMNL1-HR-SFFV-BlaR	this paper	NA
pJET-77-FMNL1-HR-SFFV-PuroR	this paper	NA
Lentiviral or Sleeping beauty vectors		
pLVX-GFP-FMNL1 beta	E. W. Miller and Blystone, 2019	NA
pCHD-EF1a-tagBFP-T2A-TetOn3G	Gemperle et al., 2022	Addgene Plasmid #179888
pLenti Lifeact-iRFP670 BlastR	Padilla-Rodriguez et al., 2018	Addgene Plasmid #84385
pCDH-tagBFP-T2A-mycBirA-Rab25 wt	Wilson et al., 2023	NA
pCDH-tagBFP-T2A-mycBirA	Wilson et al., 2023	NA
pCDH-tagBFP-T2A-mycBirA-Rab25 dC	this paper	NA
pCDH-tagBFP-T2A-mycBirA-Rab25 dN (T26N)	this paper	NA
pCDH-tagBFP-T2A-mycBirA-Rab25 dN(T26N)/dC	this paper	NA
pCDH NB_{GFP}-mCherry-Rab25 wt	this paper	NA
pCDH NB_{GFP}-mCherry-Rab25 dC	this paper	NA
pCDH NB_{GFP}-mCherry-Rab25 dN (T26N)	this paper	NA
pCDH NB_{GFP}-mCherry-Rab25 dN(T26N)/dC	this paper	NA
pCDH NB_{GFP}-mCherry	this paper	NA
pCDH GFP-RhoA wt	Caswell lab	NA
pCDH GFP-RhoA Q63L	Caswell lab	NA
pCDH GFP-RhoA T19N	Caswell lab	NA
pSBtet-Kif5b-GFP-pMag$(fast1)_{3x}$	this paper	Na
pCDH_$iRFP670_{3x}$-RBD_{4x}	this paper	Na
Bacterial Expression		
pET21_H6-mEGFP-Mms6[112-132]	Kappen, 2023	NA
Transient transfection		
pEGFP-FKBP	Stephen Royle lab	NA
pEGFP FKBP R25	Wilson et al., 2023	NA
pEGFP FKBP	Wilson et al., 2023	NA
pmCherry-Rab25 human	Caswell et al., 2007	NA
pMito-iRFP670-FRB	Gemperle et al., 2022	NA
Other plasmids used for cloning		
pB72 Kif5b GFP PDZ	van Bergeijk et al., 2015	NA
pCDH-NB_{GFP}-mCherry-Rab11	Gemperle et al., 2022	
pSBtet puroR BFP	Kowarz et al., 2015	Addgene Plasmid #60496

***Note** : Maps of newly generated plasmids are accessible from Figshare*

plasmids created in this paper will be send to Addgene or provided upon reasonable request.

Table S2. RNP and synthetized sequences

IDT company		Sequence	Note
crRNA	Rab25 specific	CCTCCATGCGGAGCCAAGAT	complementary to anti-sense ssDONOR, targeting exon 1
crRNA	FMNL1 specific	AGCAGCTGGACCTTGTCTGG	complementary to sense ssDONOR, targeting exon 2
crRNA	FMNL1 specific	CATGAACTTGCCCCCAGACA	complementary to anti-sense ssDONOR, targeting exon 2
tracrRNA	Alt-R® CRISPR-Cas9 tracrRNA		cat. n. 1072532
Cas9	Alt-R S.p. HiFi Cas9 Nuclease V3		cat. n. 1081061

SYNTHETIZED SEQUENCES		Company	Note
iRFP670$_{1x}$-RBD$_{4x}$	ATACTTGCCAACGCCAGGGTGGTATCTAGAGGATCCGCCACCATGGCGAGAAAGGTGGACCTGACCAGCTGCGACAGAGAGCCCATTCACATCCCCGGCAGCATCCAGCCTT<br>GTGGATGTCTGCTGGCCTGTGATGCCCAGGCCGTGCGGATCACAAGAATCACCGAAAACGCTGGCGCCTTCTTCGGCCGGGAAACACCTAGAGTTGGAGAGCTGCTGGCCGA<br>CTACTTCGGCGAGACTGAAGCCCACGCTCTGAGAAATGCCCTGGCTCAGAGCAGCGACCCTAAGAGGCCTGCTCTGATCTTCGGCTGGCGAGATGGACTGACCGGCAGAACC<br>TTCGACATCAGCCTGCACAGACACGACGGCACCAGCATCATCGAGTTTGAACCTGCCGCCGCTGAGCAGGCCGACAATCCTCTGAGACTGACCAGACAGATCATTGCCCGGAC<br>CAAAGAGCTGAAGTCCCTGGAAGAAATGGCCGCCAGAGTGCCCAGATACCTGCAGGCCATGCTGGGCTACCACAGAGTGATGCTGTACAGATTCGCCGACGACGGCAGCGG<br>CATGGTTATTGGAGAGGCCAAGAGAAGCGACCTCGAGTCCTTCCTGGGACAACACTTTCCCGCAAGTCTGGTCCCACAGCAAGCACGCCTGCTCTACCTTAAGAATGCTATTC<br>GCGTGGTGTCTGACTCTCGGGGCATCAGCTCAAGAATTGTCCCAGAGCACGACGCTAGTGGCGCAGCCCTGGATCTTAGTTTCGCCCATCTGCGGTCCATTAGTCCTTGTCATC<br>TGGAATTCCTTCGCAATATGGGTGTTAGCGCTAGCATGTCTCTCTCCATCATTATCGACGGGACGTTGTGGGGACTGATTATATGTCATCACTATGAGCCCCGTGCCGTTCCAA<br>TGGCACAACGGGTCGCAGCAGAAATGTTTGCTGATTTTCTCTCCCTCCATTTCACTGCCGCACACCACCAGAGGATCAGCGGCACCGGTAGCATTCTGGAAGATCTGAACATG<br>CTGTATATCCGGCAGATGGCCCTGTCACTGGAAGATACAGAGCTGCAGCGGAAGCTGGACCACGAGATCAGAATGAGAGATGGCGCCTGCAAACTGCTCGCCGCCTGTTCTC<br>AGAGAGAGCAGGCTCTGGAAGCCACCAAGTCTCTGCTCGTGTGCAACTCCAGAATCCTGAGCTACATGGGAGAACTGCAGCGGCGGAAAGAGGCCCAGGTCCTGGAAAAGA<br>CCGGAAGAACCGCCTCCATCCTTGAGGACCTGAATATGCTCTACATTCGCCAGATGGCACTGAGCTTGGAGGACACCGAGCTGCAGAGAAAACTGGATCATGAGATTCGGAT<br>GCGCGACGGGGCATGCAAGCTGTTGGCTGCTTGTAGCCAGCGCGAACAAGCCCTTGAGGCCACCAAAAGCCTGCTTGTGTGTAACAGCCGCATCCTGTCCTACATGGGCGAG<br>CTTCAACGCAGAAAAGAAGCACAGGTTTTGGAAAAGACTGGCCGCAGCGGCGTGATCTTGGAGGATCTCAATATGTTGTACATCAGACAAATGGCCCTCAGCCTTGAGGATA<br>CTGAACTCCAGAGAAAGCTCGATCACGAAATCCGGATGCGTGATGGGGCTTGTAAACTCCTGGCCGCCTGCAGCCAGAGAGAACAAGCACTCGAAGCTACAAAATCCCTGCT<br>CGTCTGTAATAGCCGGATTCTCTCTTATATGGGCGAATTGCAAAGACGCAAAGAAGCCCAAGTCCTTGAGAAAACAGGCAGCGGAGCCGGAAGCGGAGGCATCCTCGAAGAT<br>CTTAATATGCTCTATATTAGGCAAATGGCTCTGAGTCTCGAAGATACGGAACTTCAACGGAAACTCGACCATGAGATACGGATGAGAGATGGTGCATGTAAATTGCTGGCTGC<br>ATGCTCCCAACGCGAGCAGGCCCTTGAAGCTACTAAGAGCCTCCTCGTCTGCAATTCTCGGATTCTCAGCTATATGGGAGAGCTTCAAAGACGGAAAGAGGCTCAAGTTTTGG<br>AGAAAACCGGCTGAGTCGACATAGTCTAC	IDT Gene block, dsDNA	*combined with pCDH vector and iRFP670 $_{2x}$ via Gibson assembly and XhoI cloning site*
iRFP670$_{2x}$-XhoI	CAGCGGCATGGTTATTGGAGAGGCCAAGAGAAGCGACCTCGAGAGCTTTCTGGGCCAGCACTTCCCTGCTTCTCTGGTGCCTCAGCAGGCCAGACTGCTGTACCTGAAGAAC<br>GCCATCAGAGTGGTGTCCGACAGCAGAGGCATCAGCAGCAGAATCGTGCCTGAGCACGATGCCAGCGGAGCTGCTCTGGATCTGAGCTTTGCCCACCTGAGATCTATCAGCC<br>CCTGCCACCTGGAATTTCTGCGGAACATGGGAGTGTCCGCCAGCATGTCCCTGAGCATCATCATCGATGGCACCCTGTGGGGCCTGATCATCTGCCACCACTACGAGCCTAGA<br>GCCGTGCCTATGGCACAGAGGGTTGCCGCCGAAATGTTCGCCGATTTCCTGAGCCTGCACTTCACCGCCGCTCACCATCAAAGACAAGGCGGATCTGGCGGAGCCCGCAAAG<br>TGGATCTGACCTCCTGTGATCGCGAACCTATCCACATTCCTGGCTCCATCCAGCCATGCGGCTGCCTGCTTGCTTGTGACGCTCAGGCTGTCAGAATCACCCGGATCACCGAGA<br>ATGCCGGGGCTTTCTTTGGCCGCGAGACTCCAAGAGTGGGCGAACTGCTGGCTGATTATTTGGCGAAACCGAGGCTCACGCCCTGAGGAATGCTCTTGCCCAGTCCAGCGA<br>TCCCAAAAGACCCGCACTGATCTTTGGCTGGCGTGACGGCCTCACAGGCCGGACCTTTGATATCTCTCTGCACCGGCACGACGGAACCTCTATCATTGAGTTCGAGCCAGCTGC<br>TGCCGAACAGGCTGACAACCCACTGAGGCTCACCCGGCAGATTATCGCCAGAACAAAAGAACTCAAGAGCCTCGAAGAGATGGCTGCCCGGGTGCCAAGATATCTCCAAGCT<br>ATGCTGGGATATCACCGGGTCATGCTCTATCGCTTCGCCGATGATGGCTCTGGCATGGTCATCGGGGAAGCCAAGCGATCCGACCTGGAAAGCTTCCTCGGACAGCATTTTCC<br>AGCCAGCCTCGTTCCACAGCAGGCTCGGCTGCTGTATCTCAAAAATGCCATCCGGGTTGTGTCCGATAGCCGGGGCATCTCTTCCAGGATTGTGCCAGAACATGACGCCTCTG<br>GCGCCGCTCTGGACCTGTCTTTTGCTCATCTGCGGAGCATCTCCCCATGCCATCTTGAGTTTCTCAGAAATATGGGCGTCAGCGCCTCCATGAGCCTGTCCATCATCATTGACG<br>GAACACTCTGGGGACTCATCATTTGTCACCATTACGAACCACGGGCTGTGCCAATGGCTCAACGAGTGGCCGCTGAGATGTTTGCCGACTTTCTGTCTCTGCACTTTACAGCTG<br>CCCACCATCAGCGTCAAGGTGGAAGTGGCGGAGCTAGGAAAGTGGACCTCACATCCTGCGACCGCGAGCCAATCCATATTCCAGGATCTATTCAACCCTGCGGCTGTCTCCTG<br>GCATGCGACGCTCAAGCAGTGCGCATCACCAGAATTACAGAGAATGCAGGCGCATTTTTCGGCAGAGAAACCCCACGTGTCGGCGAGCTGCTCGCAGATTACTTTGGAGAGA<br>CAGAGGCACATGCACTGCGGAACGCTCTGGCTCAGTCCTCTGATCCTAAACGCCCCGCTCTGATTTTTGGCTGGCGCGACGGATTGACCGGCCGCACATTTGACATTTCCCTGC<br>ATCGGCATGATGGCACCTCCATTATCGAATTTGAGCCCGCAGCCGCAGAACAGGCAGATAACCCTTTGCGGCTGACACGCCAGATTATTGCTCGCACGAAAGAACTGAAATCA<br>CTTGAAGAGATGGCAGCTAGGGTGCCCCGGTATCTTCAGGCTATGCTCGGTTACCATCGTGTCATGTTGTATCGCTTTGCTGACGACGGCTCCGGAATGGTCATAGGCGAGGC<br>TAAACGCAGCGATCTCGAGTCCTTCCTGGGACAACACTTTCCCGCAAGTCTGGTCC	IDT Gene block, dsDNA	*combined with pCDH vector and iRFP670 $_{1x}$ -RBD $_{4x}$ via Gibson assembly and XhoI cloning site*
UNIVERSAL human FMNL1 donor (HR)	GAGAGCGGCCGCCAGATCTTCCGGATGGCTCGAGTTTTTCAGCAAGATGCACTGGTGCCCTGGGCCTGGTCTGTGTACACGTGGGATCTGGTGGCACGTGGA<br>GGTGGGAcactggccactggccactggccacACTTTGGCCTGACTGGTGATGGCTCCCCTGTTAGCTTCCCACCAGAATGGGGGAAGAGATGATGCCCCAACAGGAA<br>AACTGGGGCCCAGAAGTGTCTCAGGTCCCCAGAGAGGTAGGCTGACAACCCACGCTCCTGCCTGCACACCAGGCAGGCCAGGGAGGCGGTGTGGGGGGTG<br>ACACAGCGcaggcagactcggattcaaatcctgctctcccttctagctggtgtgccccgggaaaggtgctctgtctctcttggccttggtcttctcattggataacactacgtatctcctagggtcagagga<br>ttcatgggggtgatgcaCCTCCTAGGGAGCCAAGTGGGTTTCCCCCAAATGCCCATTCTCCCCTCTCCCCTTGTTGGGGCCCCAGGCTCAGGGGTCTTTGTCCTCCCTG<br>TCCCCAGAACTGCATGAACTTGCCCCCAGCTAGCATGGGATCGGGTGGGACTAGGTAACGCCATTTTGCAAGGCATGGAAAAATACCAAACCAAGAATAGAG<br>AAGTTCAGATCAAGGGCGGGTACATGAAAATAGCTAACGTTGGGCCAAACAGGATATCTGCGGTGAGCAGTTTCGGCCCCGGCCCGGGGCCAAGAACAGAT<br>GGTCACCGCAGTTTCGGCCCCGGCCCGAGGCCAAGAACAGATGGTCCCCAGATATGGCCCAACCCTCAGCAGTTTCTTAAGACCCATCAGATGTTTCCAGGCTC<br>CCCCAAGGACCTGAAATGACCCTGCGCCTTATTTGAATTAACCAATCAGCCTGCTTCTCGCTTCTGTTCGCGCGCTTCTGCTTCCCGAGCTCTATAAAAGAGCTCA<br>CAACCCCTCACTCGGCGCGCCAGTCCTCCGACAGACTGAGTCGCCCGGGGGGGATCTGGAGCTCTCGAGAATTCTCACGCGTgccaccatggtcACTAGTGGCAG<br>CGGAGAGACCGGTGACAAGGTCCAGCTGCTGAGCCAGTATGACAACGAGAAGAAGTGGGAGCTCATCTGTGATCAGGTAGGTGCCAGCACTGCCCAGCCAC<br>CCCTTCCCTCCCACTGTCAGTACCCCACCCTGACCAGGCTGGGGCTATGGGGAGAGGGGCACCGCCATACTGCCCATGGAGCCAAGACTCTGCTCAGTGGCCA<br>ATGTAGCACCTGGGGAGGGTGCTGGGGTCTGCAGGACTCCCAAATCCCTTTCTCTTGAACTCCCCAAAAGACCTTGTGAGTCACTAGCAGGGCTGGGTCAGCT<br>TCAGAGGAGCTGGCAGAGCTGGTTCAGGCTGGGGCAGCTGGGGCCTCGCACTGTCACTGTCCTCCTGCTGAGCTTCCTGAGAAACCTCTCTGGACTGGGGAA<br>GGGAGAGGGCGACCCTGGGGTTGGACATCTTCACCTGACCCCATGGGAGGGAGGGAGGAAGGGGCGGGAATTCCTGCCCTTTTGCCTTGGGTCCAGAGGA<br>GCTGCAGTTGACCAGCAACTCCCCAAGGCCAATCTTTCTAGAAGATCTCCTACAATATTCTCAGCTGCCATGG	GENEWIZ - FragmentGENE, dsDNA	*3'homology arm of ssDNA produced as 530 b (long) or 300 (short)*
MTS-pMagFast1$_{(3x)}$	GCCACCGCTAGCAGATCTGAGCTCAAGCTTCGAATTCCCGCCACCATGAAGAGCTTCATTACAAGGAACAAGACAGCCATTTTGGCAACCGTTGCTGCTACAGGTACTGCCATC<br>GGTGCCTACTATTATTACAACCAATTGCAACAGCCAactagtggatccggcggctccggaggcagcggcggttctggaggttccggtggcggctcccacactctttacgcccctggaggatacgacattatgggata<br>tttgcggcagattaggaaccgcccaaaccctcaggtcgaactggggcctgtggacacgtcatgtgccctgATTctgtgcgatctgaagcaaaaggacactccggtggtctacgcctcggaagccttcttgtatatgaccggataca<br>gcaatgcagaggtgctcggcaggaactgcagattcctgcagtcccccgacgggatggtgaaaccaaagtctactcgcaaatatgtggacTCCaacacgatcaacaccatgcggaaggccatcgaccggaacgccgaggtccag<br>gtcgaggtcgtgaactttaagaagaacggccagcggttcgtgaactttctgaccatgattccggtccgggatgaaaccggagagtacagatactccatgggattccagtgcgaaaccgaaGGCTCGAGTggcggctcgggcg<br>gatcagggggaagcggacacacactctacgcTccgggaggctacgatattatgggctacctgaggcagatccggaacagaccgaacccccaagtggaactcggccccgtggacacttcttgcgctctgatcctgtgcgacctgaa<br>gcagaaggataccccgtggtgtacgcatccgaagcgttcctctacatgactggttacagcaacgcggaggtgctgggtcgcaactgccggttcctgcagagccccgacggaatggtcaagcccaagtccactcggaaatacgtg<br>gattccaacaccatcaatactatgaggaaggctatcgataggaacgcggaagtccaagtggaggtggtcaatttcaagaagaatggccagagattcgtcaatttccttaccatgatccctgtgcgcgatgagactggagagtaccg<br>atactcaatgggcttccagtgtgaaaccgagggaACCGGTggttccggggggtcgggaggttccggacataccctctacgcgccggggggttatgacatcatgggttacctcagacagatcagaaaccggccgaacccacaag<br>tggagctgggacccgtggacacctcctgcgccctcatcctgtgtgaccttaagcagaaggacacccctgtggtgtacgcctccgaagcattcctgtatatgaccgggtacTCCaacgccgaagtgctgggacggaactgccgcttc<br>ctgcaatccccggatggaatggtgaagcctaagtcaacccgcaaatacgtggactccaacactatcaacaccatgcgcaaggccattgaccgcaatgctgaggtgcaagtggaagtggtgaacttcaagaagaacggacagcgc<br>ttcgtcaacttcctgactatgattcccgtgcgggacgaaaccggcgaataccgctacagcatggggtttcagtgcgagactgaggcggccgctggctc	IDT Gene block, dsDNA	*Cloned into pSBtet Kif5b-GFP-pMagFast1(3x). Modul pMagFast1 inserted in tandem via SpeI, NotI, AscI and KpnI restriction sites for possible optogenetic manipulation (69)*
anti-sense ssDNA of Rab25 DExCon donor (TRE3GS_NBGFP-mCherry)	ACCTCCATCTCTTGTGCTGCACTGGCTGACACCAGGGATTTGGGTTTCTATTAGGAACGGGGCAGAGGAGCCTAACTCAGCACTCTTAAGGAGGAATCAACATCATTATTTAC<br>CACAGTAGAGCATACTCAGAAGCTCCCTGACTTTTGCCCCTGCAACCAGACCCTCCCCCACTCCCTGCAGCTTTCTTTCTGAAGGAATGAGGAGCCCCAGGAGGTCTTGGTAAG<br>AGATAAAAAAACAGGAGGGGGCCCTCTCTGGCTCTATTCAATCTCTTTCTGGGTCTCTCAGGCTTCCTCTTGCTTCAGGGAACCCAACTCACCCTTGAAGACAAAGTTATAATC<br>TTCCTCAGTTCCATTCCCACCACTTCCAGCACCGGTTCCggCATCATATCGGTAAAGGCCTTTTGCCACTCCTTGAAGTTGAGCTCGGTcGAAGTTCCTATaCtttctagaGAATAGG<br>AACTTCggCTTGTACAGCTCGTCCATTCCGCCTGTGCTGTGTCTGCCTTCGGCTCTCTCGTACTGTTCCACGATGGTGTAGTCCTCGTTGTGGCTGGTGATGTCCAGCTTGATGT<br>TCACGTTGTAGGCGCCAGGCAGCTGCACGGGTTTCTTGGCCTTGTAGGTGGTTTTCACTTCGGCGTCGTAGTGGCCGCCATCCTTCAGTTTCAGCCGCTGCTTGATCTCGCCCT<br>TCAGGGCGCCATCTTCAGGGTACATTCTCTCGCTGCTGGCTTCCCAGCCCATGGTCTTTTTCTGCATCACGGGGCCATCAGAGGGAAAGTTGGTGCCCCGCAGCTTCACTTTGT<br>AGATGAACTCGCCGTCCTGCAGAGAGCTATCTTGAGTCACGGTGACCACGCCGCCGTCCTCGAAGTTCATCACTCTCTCCCACTTGAAGCCCTCGGGGAAGCTCAGCTTCAGGT<br>AGTCGGGAATATCGGCGGGGTGCTTCACGTAGGCCTTGCTGCCGTACATAAACTGAGGGCTCAGAATGTCCCAGGCAAAAGGCAGAGGGCCGCCTTTGGTCACTTTCAGCTT<br>GGCGGTCTGTGTTCCCTCGTAAGGTCTGCCCTCGCCTTCGCCTTCAATCTCGAACTCGTGGCCGTTCACGCTGCCTTCCATGTGCACCTTGAACCGCATGAATTCTTTGATGATG<br>GCCATGTTGTCCTCTTCGCCCTTGGACACCATGCCTCCGCTCGAGCCTCCGTCAGGACTAGACACGGTCACTTGGGTTCCCTGGCCCCAGTACTCGAAGCCCACGTTCACGTTG<br>CAGTAGTACACGGCGGTGTCCTCAGGCTTCAGGCTGTTCATCTGCAGGTACACGGTGTTTCTGGCGTCGTCCCTGCTGATGGTGAATCTGCCCTTCACGCTGTCCTCGTAGCTG<br>CTTCTATCGCCAGCGCTAGACATTCCGGCCACCCACTCTCGTTCTTTGCCAGGGGCCTGTCTATACCACCGCATGCTGTAGCGGTTCACGGGGAAGCCAGAGGCGGCACAAGA<br>CAGTCTCAGAGATCCGCCAGGCTGAACCAGTGCTCCGCCAGATTCAACCAGCTGCACCTGAGCCATgGAAGTTCCTATaCtttctagaGAATAGGAACTTCcGCCACTAGTCCCAC<br>CCGATCCAACCATCTTGAATTCTTTACGAGGGTAGGAAGTGGTACGGAAAGTTGGTATAAGACAAAAGTGTTGTGGAATTGAAGTTTACTCAAAAAATCAGCACTCTTTTATA<br>GGCGCCCTGGTTTACATAAGCAAAGCTTATACGTTCTCTATCACTGATAGGGAGTAAACTGGATATACGTTCTCTATCACTGATAGGGAGTAAACTGTAGATACGTTCTCTATC<br>ACTGATAGGGAGTAAACTGGTCATACGTTCTCTATCACTGATAGGGAGTAAACTCCTTATACGTTCTCTATCACTGATAGGGAGTAAAGTCTGCATACGTTCTCTATCACTGAT<br>AGGGAGTAAACTCTTCATACGTTCTCTATCACTGATAGGGAGTAAAGAATTCCTTGGCTCCGCATGGAGGGTGCAGGTGTCTTCGGGGACAGAGGCGACAAATCTGTGTGTT<br>GGCTCAATGCCCTCAACCCTCAGCTCTCAAAAGATCTGGTCCTGCCCCTCCTCTCATTGGGGGTAAGGGGACTTCTGCAGCCAGAGGGATTGGGGGCCAACTCAAGGTTAGGC<br>AGGGCCTAGGGAGGATCAGCTCTGGCAGGTGGGGGTGCTGTAAGGAAGCAGAGAGGACGGAAGCTGAGAACAGGAAGAGCGGGGTGGCTGGAGACCTGGGGCCCAGGT<br>GGGTGGAGAGATGATGACGTCAGACAGGTTCGACAGGTTGGGTGTCTGCTCTCAGCCTGGGCT	home-made syntetized ssDNA	*Combined with A2780 stably expressing tagBFP-T2A-TetOn3G (lentiviraly transduced with pCHD-EF1a-tagBFP-T2A-TetOn3G and sorted for tagBFP; (21))*

Anti-sense ssDNA of donor 77 (pJET-77-FMNL1-HR-SFFV-PuroR), short 3' HR = 300 pb	GCCACTGAGCAGAGTCTTGGCTCCATGGGCAGTATGGCGGTGCCCCTCTCCCCATAGCCCCAGCCTGGTCAGGGTGGGGTACTGACAGTGGGAGGGAAGGGGTGGCTGGGC<br>AGTGCTGGCACCTACCTGATCACAGATGAGCTCCCACTTCTTCTCGTTGTCATACTGGCTCAGCAGCTGGACCTTGTCACCGGTTCCGTCGACCCATAGAGCCCACCGCATCCCC<br>AGCATGCCTGCTATTGTCTTCCCAATCCTCCCCCTTGCTGTCCTGCCCCACCCCACCCCCCAGAATAGAATGACACCTACTCAGACAATGCGATGCAATTTCCTCATTTTATTAGG<br>AAAGGACAGTGGGAGTGGCACCTTCCAGGGTCAAGGAAGGCACGGGGGAGGGGCAAACAACAGATGGCTGGCAACTAGAAGGCACAGTCGAGGCTGATCAGCGGGTTTA<br>AACGGGCCCTCTAGATCACGCCCCTGGTTTCCTTGTCATGCACCATGTCCGAGGTCCTTCAGGAACCTCAACATCGGCCGTGACAGTAAATCCCAAGCGTTCATAAAAAGGAA<br>GGTTCCTTGGGGCTGAGGTCTCCAGAAACGCAGGAACACCCGCGCGCTCTGCCGCTTCCACTCCTGGAAGGACAACAGCACTTCCGAGACCCTTACCCTGGTGGTCGGGGGA<br>CACCCCTACCGTAGCAAGAAACCAGGCGGGCTCTTTGGGTCGATGCGGTGCGAGAAGTCCTTCCATTTGCTGTTGAGCCGCAAGTCTACTGCCGGACAATTCTGCCATCCGTG<br>GACCGATCTCAGCAAATACCGCTCCCGCTTCTACGCTCTCTGGAGTCGTCCATACGGCGACAGCGGCGCCGTCGTCGGCTACCCAGACCTTCCCAATATCCAAACCGACCCTAG<br>TCAGAAAGAGCTCCTGAAGTTCGGTTACTCTTTCGATATGCCTATCAGGGTCAACAGTATGTCGCGTAGCAGGATAGTCTGCAAAAGCGGCTGCGAGCGTCCGCACGGCTCGC<br>GGAACATCATCTCTCGTAGCCAATCTGACTGTGGGCTTATATTCCGTCATCTCGAGCCTAGGGCCGGGATTCTCCTCCACGTCACCGCATGTTAGAAGACTTCCTCTGCCCTCTC<br>CGCTGCCACTAGTgaccatggtggcACGCGTGAGAATTCTCGAGAGCTCCAGATCCCCCCCGGGCGACTCAGTCTGTCGGAGGACTGGCGCGCCGAGTGAGGGGTTGTGAGCTCT<br>TTTATAGAGCTCGGGAAGCAGAAGCGCGAACAGAAGCGAGAAGCAGGCTGATTGGTTAATTCAAATAAGGCGCAGGGTCATTTCAGGTCCTTGGGGGAGCCTGGAAACA<br>TCTGATGGGTCTTAAGAAACTGCTGAGGGTTGGGCCATATCTGGGGACCATCTGTTCTTGGCCTCGGGCCGGGGCCGAAACTGCGGTGACCATCTGTTCTTGGCCCCGGGCC<br>GGGGCCGAAACTGCTCACCGCAGATATCCTGTTTGGCCCAACGTTAGCTATTTTCATGTACCCGCCCTTGATCTGAACTTCTCTATTCTTGGTTTGGTATTTTTCCATGCCTTGCA<br>AAATGGCGTTACCTAGTCCCACCCGATCCCATGCTAGCTGGGGGCAAGTTCATGCAGTTCTGGGGACAGGGAGGACAAAGACCCCTGAGCCTGGGGCCCCAACAAGGGGAG<br>AGGGGAGAATGGGCATTTGGGGGAAACCCACTTGGCTCCCTAGGAGGtgcatcacccccatgaatcctctgaccctaggagatacgtagtgttatccaatgagaagaccaaggccaagagagacagagca<br>cctttcccggggcacaccagctagaagggagagcaggatttgaatccgagtctgcctgCGCTGTGTCACCCCCCACACCGCCTCCCTGGCCTGCCTGGTGTGCAGGCAGGAGCGTGGGTTGTCAGC<br>CTACCTCTCTGGGGACCTGAGACACTTCTGGGCCCCAGTTTTCCTGTTGGGGCATCATCTCTTCCCCCATTCTGGTGGGAAGCTAACAGGGGAGCCATCACCAGTCAGGCCAAA<br>GTgtggccagtggccagtggccagtgTCCCACCTCCACGTGCCACCAGATCCCACGTGTACACAGAC	home-made syntetized ssDNA
Anti-sense ssDNA of donor 77 (pJET-77-FMNL1-HR-SFFV-PuroR), long 3' HR = 530 pb	CTGGACCCAAGGCAAAAGGGCAGGAATTCCCGCCCCTTCCTCCCTCCCTCCCATGGGGTCAGGTGAAGATGTCCAACCCCAGGGTCGCCCTCTCCCTTCCCCAGTCCAGAGAG<br>GTTTCTCAGGAAGCTCAGCAGGAGGACAGTGACAGTGCGAGGCCCCAGCTGCCCCAGCCTGAACCAGCTCTGCCAGCTCCTCTGAAGCTGACCCAGCCCTGCTAGTGACTCAC<br>AAGGTCTTTTGGGGAGTTCAAGAGAAAGGGATTTGGGAGTCCTGCAGACCCCAGCACCCTCCCCAGGTGCTACATTGGCCACTGAGCAGAGTCTTGGCTCCATGGGCAGTAT<br>GGCGGTGCCCCTCTCCCCATAGCCCCAGCCTGGTCAGGGTGGGGTACTGACAGTGGGAGGGAAGGGGTGGCTGGGCAGTGCTGGCACCTACCTGATCACAGATGAGCTCCC<br>ACTTCTTCTCGTTGTCATACTGGCTCAGCAGCTGGACCTTGTCACCGGTTCCGTCGACCCATAGAGCCCACCGCATCCCCAGCATGCCTGCTATTGTCTTCCCAATCCTCCCCCTT<br>GCTGTCCTGCCCCACCCCACCCCCCAGAATAGAATGACACCTACTCAGACAATGCGATGCAATTTCCTCATTTTATTAGGAAAGGACAGTGGGAGTGGCACCTTCCAGGGTCAA<br>GGAAGGCACGGGGGAGGGGCAAACAACAGATGGCTGGCAACTAGAAGGCACAGTCGAGGCTGATCAGCGGGTTTAAACGGGCCCTCTAGATCACGCCCCTGGTTTCCTTGT<br>CATGCACCATGTCCGAGGTCCTTCAGGAACCTCAACATCGGCCGTGACAGTAAATCCCAAGCGTTCATAAAAAGGAAGGTTCCTTGGGGCTGAGGTCTCCAGAAACGCAGGA<br>ACACCCGCGCGCTCTGCCGCTTCCACTCCTGGAAGGACAACAGCACTTCCGAGACCCTTACCCTGGTGGTCGGGGGACACCCCTACCGTAGCAAGAAACCAGGCGGGCTCTTT<br>GGGTCGATGCGGTGCGAGAAGTCCTTCCATTTGCTGTTGAGCCGCAAGTCTACTGCCGGACAATTCTGCCATCCGTGGACCGATCTCAGCAAATACCGCTCCCGCTTCTACGCT<br>CTCTGGAGTCGTCCATACGGCGACAGCGGCGCCGTCGTCGGCTACCCAGACCTTCCCAATATCCAAACCGACCCTAGTCAGAAAGAGCTCCTGAAGTTCGGTTACTCTTTCGAT<br>ATGCCTATCAGGGTCAACAGTATGTCGCGTAGCAGGATAGTCTGCAAAAGCGGCTGCGAGCGTCCGCACGGCTCGCGGAACATCATCTCTCGTAGCCAATCTGACTGTGGGC<br>TTATATTCCGTCATCTCGAGCCTAGGGCCGGGATTCTCCTCCACGTCACCGCATGTTAGAAGACTTCCTCTGCCCTCTCCGCTGCCACTAGTgaccatggtggcACGCGTGAGAATT<br>CTCGAGAGCTCCAGATCCCCCCCGGGCGACTCAGTCTGTCGGAGGACTGGCGCGCCGAGTGAGGGGTTGTGAGCTCTTTTATAGAGCTCGGGAAGCAGAAGCGCGCGAACA<br>GAAGCGAGAAGCAGGCTGATTGGTTAATTCAAATAAGGCGCAGGGTCATTTCAGGTCCTTGGGGGAGCCTGGAAACATCTGATGGGTCTTAAGAAACTGCTGAGGGTTGGG<br>CCATATCTGGGGACCATCTGTTCTTGGCCTCGGGCCGGGGCCGAAACTGCGGTGACCATCTGTTCTTGGCCCCGGGCCGGGGCCGAAACTGCTCACCGCAGATATCCTGTTTG<br>GCCCAACGTTAGCTATTTTCATGTACCCGCCCTTGATCTGAACTTCTCTATTCTTGGTTTGGTATTTTTCCATGCCTTGCAAAATGGCGTTACCTAGTCCCACCCGATCCCATGCT<br>AGCTGGGGGCAAGTTCATGCAGTTCTGGGGACAGGGAGGACAAAGACCCCTGAGCCTGGGGCCCCAACAAGGGGAGAGGGGAGAATGGGCATTTGGGGGAAACCCACTTG<br>GCTCCCTAGGAGGtgcatcacccccatgaatcctctgaccctaggagatacgtagtgttatccaatgagaagaccaaggccaagagagacagagcacctttcccggggcacaccagctagaagggagagcaggatttgaa<br>tccgagtctgcctgCGCTGTGTCACCCCCCACACCGCCTCCCTGGCCTGCCTGGTGTGCAGGCAGGAGCGTGGGTTGTCAGCCTACCTCTCTGGGGACCTGAGACACTTCTGGGCCC<br>CAGTTTTCCTGTTGGGGCATCATCTCTTCCCCCATTCTGGTGGGAAGCTAACAGGGGAGCCATCACCAGTCAGGCCAAAGTgtggccagtggccagtggccagtgTCCCACCTCCACGT<br>GCCACCAGATCCCACGTGTACACAGAC	home-made syntetized ssDNA
Anti-sense ssDNA of donor 78 (pJET-78-FMNL1-HR-SFFV-BlaR), short 3' HR = 300 pb	GCCACTGAGCAGAGTCTTGGCTCCATGGGCAGTATGGCGGTGCCCCTCTCCCCATAGCCCCAGCCTGGTCAGGGTGGGGTACTGACAGTGGGAGGGAAGGGGTGGCTGGGC<br>AGTGCTGGCACCTACCTGATCACAGATGAGCTCCCACTTCTTCTCGTTGTCATACTGGCTCAGCAGCTGGACCTTGTCACCGGTTCCGTCGACCCATAGAGCCCACCGCATCCCC<br>AGCATGCCTGCTATTGTCTTCCCAATCCTCCCCCTTGCTGTCCTGCCCCACCCCACCCCCCAGAATAGAATGACACCTACTCAGACAATGCGATGCAATTTCCTCATTTTATTAGG<br>AAAGGACAGTGGGAGTGGCACCTTCCAGGGTCAAGGAAGGCACGGGGGAGGGGCAAACAACAGATGGCTGGCAACTAGAAGGCACAGTCGAGGCTGATCAGCGGGTTTA<br>AACGGGCCCTCTAGAttagccctcccacacataaccagagggcagcaattcacgaatcccaactgccgtcggctgtccatcactgtccttcactatggctttgatcccaggatgcagatcgagaagcacctgtcggcaccgtc<br>cgcaggggctcaagatgcccctgttctcatttccgatcgcgacgatacaagtcaggttgccagctgccgcagcagcagcagtgcccagcaccacgagttctgcacaaggtcccccagtaaaatgatatacattgacaccagtgaag<br>atgcggccgtcgctagagagagctgcgctggcgacgctgtagtcttcagagatggggatgctgttgattgtagccgttgctctttcaatgagggtggattcttcttgagacaaaggcttggccatCTCGAGCCTAGGGCCGGG<br>ATTCTCCTCCACGTCACCGCATGTTAGAAGACTTCCTCTGCCCTCTCCGCTGCCACTAGTgaccatggtggcACGCGTGAGAATTCTCGAGAGCTCCAGATCCCCCCCGGGCGACTC<br>AGTCTGTCGGAGGACTGGCGCGCCGAGTGAGGGGTTGTGAGCTCTTTTATAGAGCTCGGGAAGCAGAAGCGCGCGAACAGAAGCGAGAAGCAGGCTGATTGGTTAATTCA<br>AATAAGGCGCAGGGTCATTTCAGGTCCTTGGGGGAGCCTGGAAACATCTGATGGGTCTTAAGAAACTGCTGAGGGTTGGGCCATATCTGGGGACCATCTGTTCTTGGCCTCG<br>GGCCGGGGCCGAAACTGCGGTGACCATCTGTTCTTGGCCCCGGGCCGGGGCCGAAACTGCTCACCGCAGATATCCTGTTTGGCCCAACGTTAGCTATTTTCATGTACCCGCCC<br>TTGATCTGAACTTCTCTATTCTTGGTTTGGTATTTTTCCATGCCTTGCAAAATGGCGTTACCTAGTCCCACCCGATCCCATGCTAGCTGGGGGCAAGTTCATGCAGTTCTGGGGA<br>CAGGGAGGACAAAGACCCCTGAGCCTGGGGCCCCAACAAGGGGAGAGGGGAGAATGGGCATTTGGGGGAAACCCACTTGGCTCCCTAGGAGGtgcatcacccccatgaatcctctg<br>accctaggagatacgtagtgttatccaatgagaagaccaaggccaagagagacagagcacctttcccggggcacaccagctagaagggagagcaggatttgaatccgagtctgcctgCGCTGTGTCACCCCCCACACC<br>GCCTCCCTGGCCTGCCTGGTGTGCAGGCAGGAGCGTGGGTTGTCAGCCTACCTCTCTGGGGACCTGAGACACTTCTGGGCCCCAGTTTTCCTGTTGGGGCATCATCTCTTCCCC<br>CATTCTGGTGGGAAGCTAACAGGGGAGCCATCACCAGTCAGGCCAAAGTgtggccagtggccagtggccagtgTCCCACCTCCACGTGCCACCAGATCCCACGTGTACACAGAC	home-made syntetized ssDNA
Anti-sense ssDNA of donor 78 (pJET-78-FMNL1-HR-SFFV-BlaR), long 3' HR = 530 pb	CTGGACCCAAGGCAAAAGGGCAGGAATTCCCGCCCCTTCCTCCCTCCCTCCCATGGGGTCAGGTGAAGATGTCCAACCCCAGGGTCGCCCTCTCCCTTCCCCAGTCCAGAGAG<br>GTTTCTCAGGAAGCTCAGCAGGAGGACAGTGACAGTGCGAGGCCCCAGCTGCCCCAGCCTGAACCAGCTCTGCCAGCTCCTCTGAAGCTGACCCAGCCCTGCTAGTGACTCAC<br>AAGGTCTTTTGGGGAGTTCAAGAGAAAGGGATTTGGGAGTCCTGCAGACCCCAGCACCCTCCCCAGGTGCTACATTGGCCACTGAGCAGAGTCTTGGCTCCATGGGCAGTAT<br>GGCGGTGCCCCTCTCCCCATAGCCCCAGCCTGGTCAGGGTGGGGTACTGACAGTGGGAGGGAAGGGGTGGCTGGGCAGTGCTGGCACCTACCTGATCACAGATGAGCTCCC<br>ACTTCTTCTCGTTGTCATACTGGCTCAGCAGCTGGACCTTGTCACCGGTTCCGTCGACCCATAGAGCCCACCGCATCCCCAGCATGCCTGCTATTGTCTTCCCAATCCTCCCCCTT<br>GCTGTCCTGCCCCACCCCACCCCCCAGAATAGAATGACACCTACTCAGACAATGCGATGCAATTTCCTCATTTTATTAGGAAAGGACAGTGGGAGTGGCACCTTCCAGGGTCAA<br>GGAAGGCACGGGGGAGGGGCAAACAACAGATGGCTGGCAACTAGAAGGCACAGTCGAGGCTGATCAGCGGGTTTAAACGGGCCCTCTAGAttagccctcccacacataaccagagg<br>gcagcaattcacgaatcccaactgccgtcggctgtccatcactgtccttcactatggctttgatcccaggatgcagatcgagaagcacctgtcggcaccgtccgcaggggctcaagatgcccctgttctcatttccgatcgcgacgat<br>acaagtcaggttgccagctgccgcagcagcagcagtgcccagcaccacgagttctgcacaaggtcccccagtaaaatgatatacattgacaccagtgaagatgcggccgtcgctagagagagctgcgctggcgacgctgtagtct<br>tcagagatggggatgctgttgattgtagccgttgctctttcaatgagggtggattcttcttgagacaaaggcttggccatCTCGAGCCTAGGGCCGGGATTCTCCTCCACGTCACCGCATGTTAGAAGACTTC<br>CTCTGCCCTCTCCGCTGCCACTAGTgaccatggtggcACGCGTGAGAATTCTCGAGAGCTCCAGATCCCCCCCGGGCGACTCAGTCTGTCGGAGGACTGGCGCGCCGAGTGAGGG<br>GTTGTGAGCTCTTTTATAGAGCTCGGGAAGCAGAAGCGCGCGAACAGAAGCGAGAAGCAGGCTGATTGGTTAATTCAAATAAGGCGCAGGGTCATTTCAGGTCCTTGGGGG<br>AGCCTGGAAACATCTGATGGGTCTTAAGAAACTGCTGAGGGTTGGGCCATATCTGGGGACCATCTGTTCTTGGCCTCGGGCCGGGGCCGAAACTGCGGTGACCATCTGTTCT<br>TGGCCCCGGGCCGGGGCCGAAACTGCTCACCGCAGATATCCTGTTTGGCCCAACGTTAGCTATTTTCATGTACCCGCCCTTGATCTGAACTTCTCTATTCTTGGTTTGGTATTTT<br>TCCATGCCTTGCAAAATGGCGTTACCTAGTCCCACCCGATCCCATGCTAGCTGGGGGCAAGTTCATGCAGTTCTGGGGACAGGGAGGACAAAGACCCCTGAGCCTGGGGCCC<br>CAACAAGGGGAGAGGGGAGAATGGGCATTTGGGGGAAACCCACTTGGCTCCCTAGGAGGtgcatcacccccatgaatcctctgaccctaggagatacgtagtgttatccaatgagaagaccaaggcc<br>aagagagacagagcacctttcccggggcacaccagctagaagggagagcaggatttgaatccgagtctgcctgCGCTGTGTCACCCCCCACACCGCCTCCCTGGCCTGCCTGGTGTGCAGGCAGGAGC<br>GTGGGTTGTCAGCCTACCTCTCTGGGGACCTGAGACACTTCTGGGCCCCAGTTTTCCTGTTGGGGCATCATCTCTTCCCCCATTCTGGTGGGAAGCTAACAGGGGAGCCATCAC<br>CAGTCAGGCCAAAGTgtggccagtggccagtggccagtgTCCCACCTCCACGTGCCACCAGATCCCACGTGTACACAGAC	home-made syntetized ssDNA

Table S3. List of primers

PCR prior ssDNA preparation

ssDNA purification (donor for CRISPR/Cas9 and homologous recombination (HR) based knock-in) **using PCR product from double biotinylated forward primer**
Dynabeads™ MyOne™ Streptavidin C1 (Cat# 65001, Thermo), anti-sense ssDNA strand eluted by 20mM NaOH

Gene	primer	Name	sequence	modification	template
human Rab25	forward	169_F_Rab25_doubleBIO	AGCCCAGGCTGAGAGCAGAC	52-BIO IDT... 100 nmol, HPLC purified	pJET-24_Rab25 DExCon donor
	reverse	09_R_Rab25	ACCTCCATCTCTTGTGCTGC	none, standard desalted DNA oligo	(TRE3GS_NB$_{GFP}$-mCherry)
human FMNL1	forward	238F_dBio-FMNL1	GTCTGTGTACACGTGGGATCT	52-BIO IDT... 100 nmol, HPLC purified	pJET-78-FMNL1-HR-SFFV-BlaR or
	reverse	239R_FMNL1 long (3' HR = 530 pb)	CTGGACCCAAGGCAAAAGGG	none, standard desalted DNA oligo	pJET-77-FMNL1-HR-SFFV-PuroR
human FMNL1	forward	238F_dBio-FMNL1	GTCTGTGTACACGTGGGATCT	52-BIO IDT... 100 nmol, HPLC purified	pJET-78-FMNL1-HR-SFFV-BlaR or
	reverse	240R_FMNL1 short (3' HR = 300 pb)	GCCACTGAGCAGAGTCTTGG	none, standard desalted DNA oligo	pJET-77-FMNL1-HR-SFFV-PuroR

Specific primers were designed using https://www.ncbi.nlm.nih.gov/tools/primer-blast/

Genomic primers (for screening knock-ins)

Gene	primer	Name	sequence
human Rab25	forward	183F_Rab25 intron in	TTTGAGAGCTGAGGGTTGAG
	reverse	142 R_Rab25 intron out	TCTCTCGTTCCCTGTACACCT
	reverse	09 R_Rab25	ACCTCCATCTCTTGTGCTGC
	forward	193F_Rab25 intron out	CAGTGGGCTGTCTCTGAAGG
	reverse	129 F-mCherry	ACAAGGCCAAGAAACCCGTG
mCherry	reverse	133 R-mCherry	CCTTCGCCTTCAATCTCGAACT
**Additional primers (Sequencing)**			
promoter TRE3GS	forward	F_TRE3GS	TGTCTTATACCAACTTTCCGTACC

NOTE: "in" = inside the homologous arms used for knock in; "out" = outsidethe homologous arms used for knock in; F = forward; R = Reverse

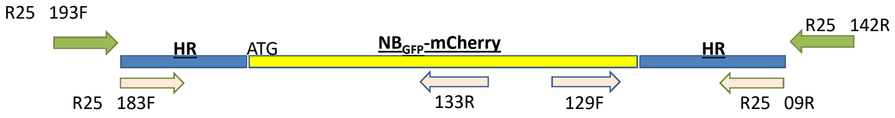

Genomic primers (for semiquantitative PCR)

Gene	primer	Name	sequence	expected size
human FMNL1, all isoforms same size	forward	251F_FMNL1_αβγ_samesize	GAATTGGGCCCAGGAGTGAA	260 pp (all same size)
	reverse	252R_FMNL1_αβγ_samesize	TCAATGAGTGTCGCCTTGCT	
human FMNL1, all isoforms, different size	forward	259F_FMNL1_αβγ_diff-size	GAGCAGGAGGTGGAACAGTG	α=338 bp; β=238 bp; γ=512 bp
	reverse	260R_FMNL1_αβγ_diff-size	CGCGGATGTAGGGCTGGTTC	(unspecific band at 1575 bp =
human FMNL1, isoforms α and γ specific	forward	253F_FMNL1_αγ	CCCAGCACCCAAGTCACC	α=196 bp; γ=374 bp
	reverse	254R_FMNL1_αγ	CTCACAGAGGAGCCGGGAT	
human FMNL1, isoforms α specific	forward	255F_FMNL1_α	AGGCCTTTGAGTCTGTGGTG	336 bp
	reverse	256R_FMNL1_α	GGGCACCGTCTTGATCACTGT	
human FMNL1, isoforms β specific	forward	2557F_FMNL1_β	GAGTTCCTGAGGGCCAACTC	393 bp
	reverse	258R_FMNL1_β	GTTCCGCAGATCTGTGATGATGTC	
human FMNL1, isoforms γ specific	forward	249R_FMNL1_γ	GCAGCAGAAGGAGCCACTCATTTATGAGAGC	254 bp
	reverse	250R_FMNL1_γ	GCACCGTCTTGATCACTGAGTGGGGGTGG	

Table S4. List of Antibodies

PRIMARY

Target		Source/Catalog number	Dilution
GAPDH	Rabbit	Sigma/G9545	WB 1:5000
α-tubulin	Mouse	Abcam/ab7291 (DM1A)	WB 1:5000
FMNL1	Rabbit	Proteintech/27834-1-AP	WB 1:2000; IF 1:400
Rab25	Rabbit	Cellsignal/13048S (D4P6P)	WB 1:1000
GFP	Mouse	Proteintech/66002-1-Ig (1E10H7)	IF 1:500
Integrin β1	Mouse	BD/610467 (18/CD29)	WB 1:1000
Integrin β1	Mouse	Abcam/5PD2	Blocking 1:100 (50)
C-Myc	Mouse	Sigma/M4439 (9E10)	WB 1:1000; IF 1:200
Integrin β1	Rat	DSHB/AIIB2	Blocking 1:100
RFP (5F8)	Rat	Chromotek/5f8-100	WB 1:750

SECONDARY

Target	Conjug	Host	Dilution	Source
Mouse IgG	80RD	Goat	WB 1:15000	Li-cor
Mouse IgG	00CW	Goat	WB 1:15000	Li-cor
Rabbit IgG	80RD	Goat	WB 1:15000	Li-cor
Rabbit IgG	00CW	Goat	WB 1:15000	Li-cor
Rat IgG	Alexa Flu	Goat	WB 1:15000	Invitrogen
Mouse IgG	Alexa Flu	Goat	IF 1:1000	Jackson Immuno Research
Rabbit IgG	Alexa Flu	Goat	IF 1:1000	Jackson Immuno Research
Rabbit IgG	Cy3	Goat	IF 1:1000	Jackson Immuno Research
Rabbit IgG	Alexa Flu	Goat	IF 1:700	Jackson Immuno Research
Rat IgG	Alexa 555	Goat	IF 1:1000	Jackson Immuno Research
Rabbit IgG	Alexa-633	Goat	IF 1:1000	Jackson Immuno Research
Rabbit IgG	non-conj	Goat	Blocking ctrl 1:100	Jackson Immuno Research

Table S5. List of resources and chemicals

Name	Source	Identifier
Software and Algorithms		
FIJI v1.51	ImageJ	fiji.sc
Prism 8.0	GraphPad	graphpad.com/scientific-software/prism
FlowJo_V10	BD Bioscience	flowjo.com
RTCA eSight software	Agilent	agilent.com
Imaris v10.0	Bitplane	imaris.oxinst.com
Matlab R2024a	MathWorks	mathWorks.com
Microscopy Image Browser	University of Helsinki	https://mib.helsinki.fi/
Other		
Dynabeads™ MyOne™ Streptavidin C1	Thermo	Cat# 65001
MagReSyn Streptavidin microspheres	ReSyn Biosciences	Cat# MR-STV010
Agencourt AMPure XP,60ml (SPRI beads)	Beckman Coulter	Cat# A63881
Phalloidin-Alexa633	Invitrogen	Cat# A22284
Streptavidin DyLight®-800 (1:5000)	Thermo	Cat# 21851
Proteinase K	Biolab	Cat# P8102S
blasticidin S HCl	Gibco	Cat# A1113903
Puromycin Dihydrochloride	Thermo	Cat# A1113803
doxycycline hydrochloride	Sigma	Cat# D3447
Ampicillin	Sigma	Cat# 59349
Chloramphenicol	Sigma	Cat# 31667
Nocodazole	Sigma	Cat# M1404-2MG
SMIFH2	Sigma	Cat# S4826
Cytochalasin D	Sigma	Cat# C8273
CK666	Sigma	Cat# 182515-25M
Biotin	Sigma	Cat# B4639
Rapamycin	Sigma	Cat# R8781-200UL
IPTG (isopropyl β-D-1-thiogalactopyranoside)	Thermo	Cat# I6758
ciprofloxacin	Sigma	Cat# 17850-25G-F
Combimag	OZ Biosciences	Cat# CM20200
E. coli BL21-CodonPlus-RIL	Agilent	Cat# 230240
1x Opti-Klear medium	Marker Gene Tech.	Cat# M1898
RPMI-1640 medium	Sigma	Cat# R8758
Dulbecco’s Modified Eagles Medium (DMEM) (D5796, Sigma	Sigma	Cat# D5796
2% gelatin solution	Sigma	Cat# G1393
tracrRNA	Alt-R® CRISPR-Cas9 tracrRNA	Cat# 1072532
Alt-R S.p. HiFi Cas9 Nuclease V3	IDT	Cat# 1081061
Lipofectamine™ CRISPRMAX™ Cas9 Transfection Reagent	Thermo	Cat# CMAX00008
Silencer select siRNA neg. control	Thermo	Cat# 4390843
Silencer select siRNA anti-FMNL1 s26	Thermo	Cat# 4392420, ID: s2226
Silencer select siRNA anti-FMNL1 s27	Thermo	Cat# 4392420, ID: s2227
Silencer select siRNA anti-FMNL1 s28	Thermo	Cat# 4392420, ID: s2228
pJET1.2 vector (CloneJET PCR Cloning Kit)	Thermo	Cat# K1231
Fibronectin	Sigma	Cat# F1141
solution T	Lonza	Cat# VCA-1002
RNA Gel Loading Dye (2X)	Thermo	Cat# R0641
hiTrap chelating HP column, 5 ml	Merck	Cat# GE17-0408-01
buffer exchange column PD10		Cat# GE17-0851-01
Amicon® Ultra-4 Centrifugal Filter Unit 100 kDa NMWCO	Merck	Cat# UFC810008
Amicon® Ultra-4 Centrifugal Filter Unit 10 kDa NMWCO	Merck	Cat# UFC8010

Supplementary movie legends

Representative live-cell imaging movies (spinning disk confocal) are separate files relevant to the study. Movies S1-S10 show spatiotemporal magnetic control of GFP-MNPs inside living A2780 cells expressing NB^{GFP}-mCherry (ctrl) or NB^{GFP}-mCherry fused with different variants of Rab25 stably co-expressing LifeAct-iRFP670 (F-actin) or active RhoA probe $iRFP670_{3x}$-RBD_{4x} (RBD_{4x}), as detailed in the accompanying movie legends. For full-resolution and additional movies see https://doi.org/10.6084/m9.figshare.22155083.

Movie S1

Timelapse video (3i Marianas spinning disk) of remote magnetic manipulation of GFP-MNPs (biop-SpringGreen LUT) inside living A2780 cells stably expressing LifeAct-iRFP670 (F-actin, gem LUT) on FN-coated coverslips. Merge: GFP-MNPs/F-actin/Brightfield, left. Magnetic tweezers/tip visible as shadow in brightfield. GFP-MNPs relocalization to various subcellular locations, red arrow. Timelapse covers total 18 min 33 s with frame taken every 3.18 s (approximately 67 s elapsed time per second of the movie). Scale bar, 10 µm. GFP-MNPs delivered by microinjection. Selected frames from this movie are shown in fig. S2C. See also additional relevant extra movies S11-S13 accessible via https://doi.org/10.6084/m9.figshare.22155083.

Movie S2

Timelapse video (3i Marianas spinning disk) showing remote manipulation of endogenous Rab25 endosomes demonstrating a direct role in cell protrusion. A2780 DExCon-modified NB^{GFP}-mCherry-Rab25 (biop-Amber LUT) cells dox pre-treated (>94 h; 250 ng/ml) stably expressing LifeAct-iRFP670 (F-actin, gem or biob-Azure LUT) on FN-coated coverslips. GFP-MNPs delivered by microinjection (biop-SpringGreen LUT). Individual or merged channels as indicated in the movie. Magnetic tweezers/tip visible as shadow in brightfield (left). GFP-MNPs/NB^{GFP}-mCherry relocalization followed by local F-actin-dependent protrusion changes, yellow arrows (with cytochalasin D treatment, red arrows). The timelapse video comprises a total duration of 76 minutes, with a frame shown every 30 s (00:00 – 67:10; approximately 450 s elapsed time per second of the movie) or 12 s (71:08 – 76:32; approximately 180 s elapsed time per second of the movie; cytochalasin D treatment from time 68:00), as indicated by the depicted time interval. Note: fast 2s or 5s time interval was captured for the adjustment and re-adjustment of the magnet tip position, but for the movie clarity resliced to 30s (or 12s, respectively) interval. Scale bar, 20 µm. Selected key frames from this movie are shown in Fig. 1E. See also additional relevant movies S14-S15 accessible via https://doi.org/10.6084/m9.figshare.22155083.

Movie S3

Timelapse video (Andor Dragonfly spinning disk) showing magnetic manipulation of membrane-free Rab25 in A2780 ovarian cancer cells to control protrusion outgrowth. A2780 stably co-expressing LifeAct-iRFP670 (F-actin, gem LUT) with NB^{GFP}-mCherry-Rab25 dC mutant (biop-Amber LUT) on FN-coated coverslips. GFP-MNPs delivered by microinjection (biop-SpringGreen LUT). Individual or merged (GFP-MNPs/F-actin/brightfield) channels as indicated in the movie. Magnetic tweezers/tip visible as shadow in brightfield (right). Gradient of NB^{GFP}-mCherry- Rab25 dC and GFP-MNPs induced by the magnet, blue arrows; protrusion growth reflected with F-actin. Timelapse covers total 45 min with frame taken every 5 s (approximately 135 s elapsed time per second of the movie). Scale bar, 20 µm. Selected key frames from this movie (time stamper here starts from t=5, just before approaching with the magnet) are shown in Fig. 2E.

Movie S4

Timelapse video (3i Marianas spinning disk) showing magnetic manipulation of endogenous Rab25 recycling endosomes in living cells migrating in 3D-Cell-Derived Matrix (CDM) to control F-actin protrusion. A2780 DExCon-modified NB^{GFP}-mCherry-Rab25 cells (Red or Red Hot LUT) dox pre-treated (72 h; 250 ng/ml) expressing Lifeact-iRFP670 (F-actin, Grey or gem LUT). GFP-MNPs (Green LUT) delivered by microinjection and repeatedly relocalized using home-made magnetic tip, shadow in merge brightfield/F-actin (middle). Enrichment of endosome-bound NB^{GFP}-mCherry- Rab25 and protrusion growth reflected with F-actin, yellow arrow. 100 µM CK666 and 5 µM SMIFH2 treatment as depicted in the movie. The timelapse video comprises a total duration of 128 minutes, with a frame shown every 30 s, as indicated by the depicted time interval (approximately 450 s elapsed time per second of the movie). Note: 4s interval was captured for the adjustment and re-adjustment of the magnet tip position as depicted in Fig. 3H, but for the movie clarity resliced to 30s interval. Scale bar, 20 µm. Selected key frames from this movie are shown in Fig. 3A-F.

Movie S5

Timelapse video, captured using the Andor Dragonfly spinning disk, illustrates the magnetic manipulation of Rab25 recycling endosomes in living cells migrating in 3D-Cell-Derived Matrix (CDM) to control F-actin

protrusion which is abrogated by integrin β1 blockade. A2780 NB^{GFP}-mCherry-Rab25 wt (biop-Amber LUT) cell stably expressing LifeAct-iRFP670 (F-actin, biob-Azure LUT) on FN-coated coverslips. GFP-MNPs delivered by microinjection (biop-SpringGreen LUT, middle). Individual or merged channels as indicated in the movie. Magnetic tweezers/tip visible as shadow in merge brightfield/F-actin (right). Original main protrusion without magnetic tip, yellow arrowheads. GFP-MNPs/NB^{GFP}-mCherry-Rab25 relocalization followed by local F-actin-dependent protrusion changes, orange arrowheads. Integrin β1 blocking antibody treatment indicated (AIIB2, 10 µg/ml; 27:00) followed by protrusion retraction/cell rounding, red arrowheads. The timelapse video comprises a total duration of 111 minutes, with a frame shown every 10 s, as indicated by the depicted time interval (approximately 150 s elapsed time per second of the movie). Scale bar 10 µm. Selected key frames from this movie are shown in fig. S8G.

Movie S6

Timelapse video (3i Marianas spinning disk) showing magnetic re-localization of endogenous membrane-bound Rab25 in FMNL1 depleted cells migrating on FN-coated coverslip. A2780 DExCon-modified NB^{GFP}-mCherry-Rab25 FMNL1+/- cells (biop-Amber LUT; dox treated > 94h; 250 ng/ml) stably expressing Lifeact-iRFP670 (F-actin, gem or biob-Azure LUT), microinjected with GFP-MNPs (biop-SpringGreen LUT) and nucleofected with chemically modified $siRNA_{pool}$ anti-FMNL1 (FMNL1 depleted cells). Magnetic tweezers/tip visible as shadow in brightfield/F-actin merge (left). Zoomed inset, merge F-actin/GFP-MNPs/NB^{GFP}-mCherry-Rab25; magnetic enrichment, red arrow (no F-actin protrusion outgrowth). Timelapse covers total 37 min with frame taken every 2s (00:00-01:10; approximately 42 s elapsed time per second of the movie) before magnetic approach, 5s (03:00-05:15; approximately 105 s elapsed time per second of the movie) for initial adjustment of the magnet tip position or 30 s (approximately 630 s elapsed time per second of the movie) for long term imaging. Scale bar 10 µm. Movie relevant to Fig. 5B bottom.

Movie S7

Timelapse video (3i Marianas spinning disk) showing magnetic manipulation of endogenous Rab25 recycling endosomes in living cells migrating in 3D-Cell-Derived Matrix (CDM) to control F-actin filopodia based protrusions. A2780 DExCon-modified NB^{GFP}-mCherry-Rab25 FMNL1+/+ cells (biop-Amber LUT; dox treated > 94h; 250 ng/ml) stably expressing Lifeact-iRFP670 (F-actin, gem or grey or biob-Azure LUT), microinjected with GFP-MNPs (biop-SpringGreen LUT) and nucleofected with non-targeting ctrl siRNA. Individual or merged channels as indicated in the movie. The magnetic tweezers/tip is discernible as a left shadow in brightfield/F-actin merge (top middle). The relocalisation of GFP-MNPs and endosomal NB^{GFP}-mCherry-Rab25 towards a magnetic tip correlates with the polymerisation of F-actin and the formation of filopodia-based protrusions, as indicated by the yellow and red arrowheads. Timelapse covers total 75 min 25 s with frame taken every 5 s, as indicated by the depicted time interval (approximately 155 s elapsed time per second of the movie). Scale bar 10 µm. Selected key frames from this movie are shown in Fig. 5D top.

Movie S8

Timelapse video (3i Marianas spinning disk) showing magnetic re-localization of endogenous membrane-bound Rab25 in FMNL1 depleted cells migrating in 3D CDM. A2780 DExCon-modified NB^{GFP}-mCherry-Rab25 FMNL1+/- cells (biop-Amber LUT; dox treated > 94h; 250 ng/ml) stably expressing Lifeact-iRFP670 (F-actin, gem or biob-Azure LUT), microinjected with GFP-MNPs (biop-SpringGreen LUT) and nucleofected with chemically modified $siRNA_{pool}$ anti-FMNL1 (FMNL1 depleted cells). Magnetic tweezers/tip visible as shadow in brightfield/GFP-MNPs or brightfield/F-actin merge (left). Merge F-actin/GFP-MNPs/NB^{GFP}-mCherry-Rab25; magnetic enrichment, red arrow (no F-actin protrusion outgrowth). Timelapse covers total 79 min with frame taken every 10 s (00:00-07:10; approximately 300 s elapsed time per second of the movie) for initial adjustment of the magnet tip position or 30 s (07:10-78:40; approximately 900 s elapsed time per second of the movie) for long term imaging. Scale bar, 10 µm. Movie relevant to Fig. 5D bottom.

Movie S9

Timelapse video (Andor Dragonfly spinning disk) showing local modulation of RhoA activity by magnetic re-localization of endogenous membrane-bound Rab25 in A2780 ovarian cancer cells migrating on FN-coated coverslip. A2780 DExCon-modified NB^{GFP}-mCherry-Rab25 (biop-Amber LUT) cells dox pre-treated (> 72 h; 250 ng/ml) stably co-expressing active RhoA probe $iRFP670_{3x}$-RBD_{4x} (RBD_{4x}; gem LUT). GFP-MNPs delivered by microinjection (biop-SpringGreen LUT). Magnetic tweezers/tip visible as shadow in brightfield/ RBD_{4x} (top middle). Individual or merged channels as indicated in the movie. Sustained attraction of Rab25 endosomes via bound GFP-MNPs and increased localisation of the active Rho probe in a punctate pattern (top right), yellow arrowheads. Timelapse covers total 25 min with frame taken every 5 s (approximately 150 s elapsed time per second of the movie, respectively). Scale bar, 20 µm. Movie relevant to Fig. 6C.

Movie S10

Timelapse video (Andor Dragonfly spinning disk) showing local modulation of RhoA activity by magnetic re-localization of membrane-free Rab25 in A2780 ovarian cancer cells migrating on FN-coated coverslip. A2780 stably co-expressing NB^{GFP}-mCherry-Rab25 dC mutant (biop-Amber LUT) with active RhoA probe $iRFP670_{3x}$-RBD_{4x} (RBD_{4x}; gem LUT). GFP-MNPs delivered by microinjection (biop-SpringGreen LUT). Magnetic tweezers/tip visible as shadow in brightfield/ RBD_{4x} (top right). Magnetic control of GFP-MNPs and NB^{GFP}-mCherry-Rab25 dC gradient is followed by local increase in the intensity of the active Rho probe, yellow arrowheads. Timelapse covers total 61 min with frame taken every 5 s (approximately 500 s elapsed time per second of the movie, respectively). Scale bar, 10 μm. Movie relevant to Fig. 6E.

Extended supplementary movie legends

Included full-resolution live-cell imaging movies (Spinning Disc) are separate files relevant to the study "Live-cell magnetic micromanipulation of recycling endosomes reveals their direct effect on actin-based protrusion to promote invasive migration" and can be accessed via https://doi.org/10.6084/m9.figshare.22155083. Movies included in S1-S10 are of particular significance for the purposes of this study. Movies S11-S22 serve to provide supplementary corroborative evidence which could not be incorporated directly with this study.

Supplementary movie S11

Timelapse video (Andor Dragonfly spinning disk) showing reversible magnetic manipulation, attraction and release kinetics of NB^{GFP}-mCherry (biop-Amber LUT) and GFP-MNPs (biop-SpringGreen LUT) in A2780 ovarian cancer cells stably co-expressing LifeAct-iRFP670 (F-actin, gem LUT) on FN-coated coverslips. F-actin shown as merge with brightfield (top), with GFP-MNPs (middle) or with NB^{GFP}-mCherry (bottom). Magnetic tweezers/tip visible as shadow in brightfield (reversible positioned in close proximity to cells or moved far away). GFP-MNPs and NB^{GFP}-mCherry relocalization to various subcellular locations, yellow arrows. GFP-MNPS delivered by microinjection. Timelapse covers total 20 min with frame taken every 3,79 s (approximately 114 s elapsed time per second of the movie). Bleaching of mCherry signal was automatically compensated for by Stack Contrast Adjustment plugin in Fiji. Scale bar, 10 µm. GFP-MNPs delivered by microinjection. Selected key frames from this movie are shown in fig. S2D. See also additional relevant extra movies S1; S12-13 accessible via https://doi.org/10.6084/m9.figshare.22155083.

Supplementary movie S12

Timelapse video (Andor Dragonfly spinning disk) of remote un-limited magnetic manipulation of GFP-MNPs (biop-SpringGreen LUT) and NB^{GFP}-mCherry (biop-Amber LUT) inside living A2780 cells stably expressing LifeAct-iRFP670 (F-actin, biob-Azure LUT) on FN-coated coverslips. Merge: GFP-MNPs/ NB^{GFP}-mCherry/F-actin, left; MNPs/ NB^{GFP}-mCherry/F-actin/Brightfield, right. Magnetic tweezers/tip visible as shadow in brightfield merge re-positioned from right to left across cells. Quantitative GFP-MNPs and NB^{GFP}-mCherry relocalization from a variety of subcellular locations, including across whole cells, in multiple cells simultaneously. GFP-MNPS delivered by microinjection. Note: In cells with GFP-MNPs microinjected levels < NB^{GFP}-mCherry levels, GFP-MNPs visible as aggregates (crosslinked) that are not amenable to movement with magnetic force (not observed with A2780 DExCon-modified NB^{GFP}-mCherry-Rab25 cells). Timelapse covers total 50 min with frame taken every 5 s (approximately 500 s elapsed time per second of the movie). Scale bar, 10 µm. See also additional relevant extra movies S1; S11; S13 accessible via https://doi.org/10.6084/m9.figshare.22155083.

Supplementary movie S13

Timelapse video (3i Marianas spinning disk) showing magnetic re-localization of both GFP-MNPs and NB^{GFP}-mCherry inside living A2780 cells without any significant visible effect on F-actin protrusions despite sustained attraction of GFP-MNPs, red arrow. A2780 cells stably co-expressing NB^{GFP}-mCherry (biop-Amber LUT; right) and LifeAct-iRFP670 (F-actin, biob-Azure LUT) on FN-coated coverslips. GFP-MNPs (biop-SpringGreen LUT; middle) delivered by microinjection. Merge: F-actin/Brightfield, left. GFP-MNPs/NB^{GFP}-mCherry/F-actin, inset (middle). Magnetic tweezers/tip visible as shadow in brightfield merge (bottom right). Note: In cells with GFP-MNPs microinjected levels < NB^{GFP}-mCherry levels, some GFP-MNPs visible as aggregates (crosslinked) that are not amenable to movement with magnetic force (not observed with A2780 DExCon-modified NB^{GFP}-mCherry-Rab25 cells). Timelapse covers total 37 min with frame taken every 12s (time 0:00 – 1:44; approx. 252 s elapsed time per second of the movie) for magnet-free phase, every 4s (3:20 – 5:00 and 33:20 – 37:20; approx. 84 s elapsed time per second of the movie) for initial and following adjustment of the magnet tip position, every 60s for long term imaging (5:00 – 33:00; approx. 1260 s elapsed time per second of the movie). Scale bar, 20 µm. Selected key frames from this movie are shown in fig. S2E. See also additional relevant extra movies S1; S11; S12 accessible via https://doi.org/10.6084/m9.figshare.22155083.

Supplementary movie S14

Timelapse video (3i Marianas spinning disk) of magnetic re-localization of endogenous membrane-bound Rab25 in A2780 ovarian cancer cells with visible effect on F-actin protrusions which was not blocked by Arp2/3 inhibitor CK666 (100 µM; time indicated in the movie). A2780 DExCon-modified NB^{GFP}-mCherry-Rab25 (biop-Amber LUT) cells dox pre-treated (>94 h; 250 ng/ml) stably expressing LifeAct-iRFP670 (F-actin, gem LUT) on FN-coated coverslips. GFP-MNPs delivered by microinjection (biop-SpringGreen LUT). Individual or merged channels as indicated in the movie. Magnetic tweezers/tip visible as shadow in brightfield/GFP-MNPs (left; boxed area show zoomed insets) or brightfield/F-actin/GFP-MNPs (middle left) merge. GFP-MNPs (GFP-nanop.) and NB^{GFP}-mCherry (Nan-mCh) relocalization followed by local F-actin dependent protrusion changes, yellow arrows. Timelapse video comprises a total duration of 103 minutes, with a frame captured every 2s (time 0:00 – 6:30; approx. 120 s elapsed time per second of the movie) for initial and following adjustment of the magnet tip position (cells focused without magnetic field, then approached with magnetic tip at 01:28 with 1 minute lag not visualized on the time stamp) or 30s interval (6:30 – 74:00; approx. 1800 s elapsed time per second of the movie) for long term imaging followed with 2s interval prior and after CK666 treatment (74:00 – 104:56; magnetic tip re-adjustments and re-focus), see depicted stamped time interval. Scale bar, 20 µm. Selected key frames from this movie are shown in fig. S3A. See also additional relevant movies S2, S15 accessible via https://doi.org/10.6084/m9.figshare.22155083.

Supplementary movie S15

Timelapse video (3i Marianas spinning disk) of magnetic re-localization of endogenous membrane-bound Rab25 in A2780 ovarian cancer cells, treatment with formin inhibitor SMIFH2 (5 µM, later 25 µM; time indicated in the movie) blocked F-actin protrusion growth. A2780 DExCon-modified NB^{GFP}-mCherry-Rab25 (biop-Amber LUT) cells dox pre-treated (>94 h; 250 ng/ml) stably expressing LifeAct-iRFP670 (F-actin, gem LUT) on FN-coated coverslips. GFP-MNPs delivered by microinjection (gem or biop-SpringGreen LUT). Individual or merged channels as indicated in the movie. Magnetic tweezers/tip visible as shadow in brightfield/F-actin merge (middle). GFP-MNPs (GFP-nanop.), NB^{GFP}-mCherry (Nan-mCh) distribution changes and retraction of F-actin based protrusions indicated by red arrowheads. Timelapse video comprises a total duration of 42 minutes, with a frame captured every 30s (00:00 – 39:20; approx. 840 s elapsed time per second of the movie) for long term imaging followed by 4s interval (39:20 – 42:12; approx. 112 s elapsed time per second of the movie; magnetic tip re-adjustments and additional SMIFH2 treatment). Scale bar 20 µm. Selected key frames from this movie are shown in fig. S3B and fig. S4C. See also additional relevant movies S2, S14 accessible via https://doi.org/10.6084/m9.figshare.22155083.

Supplementary movie S16

Tomogram animation with segmented recycling endosomes (light blue) and GFP-MNPs (orange) in A2780 DExCon-modified NB^{GFP}-mCherry-Rab25 cells (pre-treated with dox >94 h; 250 ng/ml). GFP-MNPs delivered by electroporation. Tomogram animation movie generated from STEM tomography relevant to Fig. 1F.

Supplementary movie S17

Timelapse video (Andor Dragonfly spinning disk) showing remote manipulation of Rab25 endosomes demonstrating a direct role in cell protrusion independent of initial cell polarity. A2780 stably co-expressing NB^{GFP}-mCherry-Rab25 (left) and LifeAct-iRFP670 (F-actin, right) on FN-coated coverslips. GFP-MNPs delivered by microinjection (middle). Magnetic tweezers/tip visible as shadow in brightfield/F-actin merge (right). GFP-MNPs and NB^{GFP}-mCherry relocalization followed by local F-actin dependent protrusion changes, blue arrowheads. The timelapse video comprises a total duration of 21 minutes, with a frame shown every 30 s (approximately 210 s elapsed time per second of the movie), as indicated by the depicted time interval. Note: Approximately 25% of NB^{GFP}-mCherry-Rab25 wt overexpressing cells exhibited a higher proportion of diffusively localized Rab25 (not observed with A2780 DExCon-modified NB^{GFP}-mCherry-Rab25 cells) with fast relocalization kinetics (not included in the analysis of Magnetic attraction and release kinetics). Scale bar, 10 µm. Selected key frames from this movie are shown in fig. S5B. See also additional relevant movies S2, S4, S5, S7 and S14 accessible via https://doi.org/10.6084/m9.figshare.22155083.

Supplementary movie S18

Timelapse video (3i Marianas spinning disk) showing magnetic manipulation of NB^{GFP}-mCherry and GFP-MNPs in living cells migrating in 3D-Cell-Derived Matrix (CDM). A2780 stably co-expressing NB^{GFP}-mCherry (biop-Amber LUT; middle right) with Lifeact-iRFP670 (F-actin; gem LUT). GFP-MNPs (gem or biop-SpringGreen LUT; left and middle left) delivered by microinjection. Magnetic tweezers/tip visible as shadow in brightfield/GFP-MNPs merge (left). Timelapse covers total 14 min (12:00 – 26:00) with frame taken every 30s (approximately 300 s elapsed time per second of the movie). Scale bar, 20 µm. Movie relevant to fig. S7A.

Supplementary movie S19

Timelapse video (Andor Dragonfly spinning disk) showing that cell adaptation to mechanosensing of magnetic force by MNP-bound Rab25 endosomes is perturbed by Blebbistatin treatment. A2780 DExCon-modified NB^{GFP}-mCherry-Rab25 (bottom left; dox treated > 94 h, 250 ng/ml) stably expressing Lifeact-iRFP670 (F-actin; bottom right, gem LUT) migrating in 3D-Cell-Derived Matrix (CDM). GFP-MNPs (upper left) delivered by microinjection. Magnetic tweezers/tip visible as shadow in brightfield/F-actin merge (top right) or as indicated in the movie. Cell shape changes: adaptation to magnetic force (time 6-18 min, yellow arrowhead), swelling (51 – 111 min, yellow arrowheads) induced by Blebbistatin (5 µM) treatment (23 – 111 min), no magnet in between 23:00 –51:30. Blue arrowheads, magnetic attraction. Timelapse covers total 111 min with frame taken every 30 s (00:00 –06:00 and 23:00 – 111:00; approximately 1500 s elapsed time per second of the movie) for interval before approaching cells with magnetic tip and for long term imaging or 5s (06:00 –22:45; approximately 250 s elapsed time per second of the movie) for initial adjustment of the magnet tip position and adaptation phase. Scale bar, 20 µm. For stress maps see relevant fig. S7B (time stamper here starts from 0, 6 min before approaching cells with the magnet).

Supplementary movie S20

Timelapse video (Andor Dragonfly spinning disk) showing magnetic manipulation of membrane-free Rab25 in A2780 ovarian cancer cells in 3D-Cell-Derived Matrix (CDM) with no effect on protrusion outgrowth (red arrowhead). A2780 stably co-expressing LifeAct-iRFP670 (F-actin, gem LUT) with NB^{GFP}-mCherry-Rab25 dC mutant (biop-Amber LUT). GFP-MNPs delivered by microinjection (biop-SpringGreen LUT). Individual or merged (GFP-MNPs/F-actin/brightfield) channels as indicated in the movie. Magnetic tweezers/tip visible as shadow in brightfield/F-actin (right). Gradient of NB^{GFP}-mCherry- Rab25 dC and GFP-MNPs induced by the magnet, blue arrowheads. Timelapse covers total 23 min with frame taken every 5 s (approximately 150 s elapsed time per second of the movie). Scale bar 20 µm. Movie relevant to fig. S7C.

Supplementary movie S21

Timelapse video (3i Marianas spinning disk) showing magnetic attraction of Rab25 positive endosomal cluster that promotes formation of actin polymerization hotspot. A2780 DExCon-modified NB^{GFP}-mCherry-Rab25 (Nan-mCh-R25; biop-Amber LUT; dox treated 72 h, 250 ng/ml) stably expressing Lifeact-iRFP670 (F-actin; gem or biob-Azure LUT) migrating in 3D-Cell-Derived Matrix (CDM). GFP-MNPs (GFP-nanop.; biop-SpringGreen LUT) delivered by microinjection. Individual or merged channels as indicated in the movie. Magnetic tweezers/tip visible as shadow in brightfield/F-actin merge (bottom left) or as indicated in the movie. Red arrowhead indicates shape changes (cell adaptation to the mechanosensing of magnetic forces upon the repositioning of a magnetic tip). Yellow arrowheads indicate the formation of a visible actin polymerization hot-spot that moved and highly colocalized with Rab25 and GFP-MNPs over >1 hour, co-attracted to the magnet, and returned after its removal. Timelapse covers total 75 min with frame taken every 20 s (approximately 420 s elapsed time per second of the movie). Scale bar 20 µm. Movie relevant to fig. S11 (SciAdvances) or (fig. S12 bioRxiv version).

Supplementary movie S22

Timelapse video (Andor Dragonfly spinning disk) showing local modulation of RhoA activity by magnetic re-localization of endogenous membrane-bound Rab25 in A2780 ovarian cancer cells migrating in 3D-CDM. A2780 DExCon-modified NB^{GFP}-mCherry-Rab25 (nanob-mCh-Rab25; biop-Amber LUT) cells dox pre-treated (> 72 h; 250 ng/ml) stably co-expressing active RhoA probe $iRFP670_{3x}$-RBD_{4x} (RBD_{4x}; gem LUT). GFP-MNPs delivered by microinjection (GFP-nanop.; biop-

SpringGreen LUT). Magnetic tweezers/tip visible as shadow in merge brightfield/GFP-MNPs (top left) and brightfield/ RBD_{4x} (top middle). Individual or merged channels as indicated in the movie. Yellow arrowheads indicate redistribution of Rab25 endosomes via bound GFP-MNPs and co-redistribution of active RhoA probe in a punctate pattern towards magnetic tweezers. Transient cell contraction adaptation upon magnetic tip re-positioning visible as a local increase of active RhoA signal at the cell rear, cyan arrowhead. Timelapse covers total 30 min with frame taken every 5 s (00:00-11:22; approximately 104 s elapsed time per second of the movie) for initial adjustment of the magnet tip position or 30 s (11:22-29:10; approximately 630 s elapsed time per second of the movie) for long term imaging. Scale bar, 20 µm. Movie relevant to Fig. 6D (time stamper here starts from 0, 2 min 30 s before approaching cells with the magnet).

REFERENCES AND NOTES

1. G. Jacquemet, M. J. Humphries, P. T. Caswell, Role of adhesion receptor trafficking in 3D cell migration. *Curr. Opin. Cell Biol.* **25**, 627–632 (2013).

2. G. Scita, P. P. Di Fiore, The endocytic matrix. *Nature* **463**, 464–473 (2010).

3. M. R. Golachowska, D. Hoekstra, S. C. D. van IJzendoorn, Recycling endosomes in apical plasma membrane domain formation and epithelial cell polarity. *Trends Cell Biol.* **20**, 618–626 (2010).

4. H. Jin, Y. Tang, L. Yang, X. Peng, B. Li, Q. Fan, S. Wei, S. Yang, X. Li, B. Wu, M. Huang, S. Tang, J. Liu, H. Li, Rab GTPases: Central coordinators of membrane trafficking in cancer. *Front. Cell Dev. Biol.* **9**, 648384 (2021).

5. E. E. Kelly, C. P. Horgan, M. W. McCaffrey, Rab11 proteins in health and disease. *Biochem. Soc. Trans.* **40**, 1360–1367 (2012).

6. R. Eva, S. Crisp, J. R. K. Marland, J. C. Norman, V. Kanamarlapudi, C. Ffrench-Constant, J. W. Fawcett, ARF6 directs axon transport and traffic of integrins and regulates axon growth in adult DRG neurons. *J. Neurosci.* **32**, 10352–10364 (2012).

7. Y. Higuchi, P. Ashwin, Y. Roger, G. Steinberg, Early endosome motility spatially organizes polysome distribution. *J. Cell Biol.* **204**, 343–357 (2014).

8. L. Sadowski, I. Pilecka, M. Miaczynska, Signaling from endosomes: Location makes a difference. *Exp. Cell Res.* **315**, 1601–1609 (2009).

9. K. Vaidžiulytė, A. S. Macé, A. Battistella, W. Beng, K. Schauer, M. Coppey, Persistent cell migration emerges from a coupling between protrusion dynamics and polarized trafficking. *eLife* **11**, e69229 (2022).

10. M. Zerial, H. McBride, Rab proteins as membrane organizers. *Nat. Rev. Mol. Cell Biol.* **2**, 107–117 (2001).

11. P. T. Caswell, H. J. Spence, M. Parsons, D. P. White, K. Clark, K. W. Cheng, G. B. Mills, M. J. Humphries, A. J. Messent, K. I. Anderson, M. W. McCaffrey, B. W. Ozanne, J. C. Norman, Rab25 associates with α5β1 integrin to promote invasive migration in 3D microenvironments. *Dev. Cell* **13**, 496–510 (2007).

12. K. H. Cho, H. Y. Lee, Rab25 and RCP in cancer progression. *Arch. Pharm. Res.* **42**, 101–112 (2019).

13. C. Gebhardt, U. Breitenbach, K. H. Richter, G. Fürstenberger, C. Mauch, P. Angel, J. Hess, c-Fos-dependent induction of the small Ras-related GTPase Rab11a in skin carcinogenesis. *Am. J. Pathol.* **167**, 243–253 (2005).

14. S. Mitra, K. W. Cheng, G. B. Mills, Rab25 in cancer: A brief update. *Biochem. Soc. Trans.* **40**, 1404–1408 (2012).

15. G. S. Ray, J. R. Lee, K. Nwokeji, L. R. Mills, J. R. Goldenring, Increased immunoreactivity for Rab11, a small GTP-binding protein, in low-grade dysplastic Barrett's epithelia. *Lab. Invest.* **77**, 503–511 (1997).

16. S. Wang, C. Hu, F. Wu, S. He, Rab25 GTPase: Functional roles in cancer. *Oncotarget* **8**, 64591–64599 (2017).

17. M. Schuh, An actin-dependent mechanism for long-range vesicle transport. *Nat. Cell Biol.* **13**, 1431–1436 (2011).

18. O. Pylypenko, T. Welz, J. Tittel, M. Kollmar, F. Chardon, G. Malherbe, S. Weiss, C. I. L. Michel, A. Samol-Wolf, A. T. Grasskamp, A. Hume, B. Goud, B. Baron, P. England, M. A. Titus, P. Schwille, T. Weidemann, A. Houdusse, E. Kerkhoff, Coordinated recruitment of Spir actin nucleators and myosin V motors to Rab11 vesicle membranes. *eLife* **5**, e17523 (2016).

19. N. R. Paul, J. L. Allen, A. Chapman, M. Morlan-Mairal, E. Zindy, G. Jacquemet, L. Fernandez del Ama, N. Ferizovic, D. M. Green, J. D. Howe, E. Ehler, A. Hurlstone, P. T. Caswell, α5β1 integrin recycling promotes Arp2/3-independent cancer cell invasion via the formin FHOD3. *J. Cell Biol.* **210**, 1013–1031 (2015).

20. G. Jacquemet, D. M. Green, R. E. Bridgewater, A. von Kriegsheim, M. J. Humphries, J. C. Norman, P. T. Caswell, RCP-driven α5β1 recycling suppresses Rac and promotes RhoA activity via the RacGAP1–IQGAP1 complex. *J. Cell Biol.* **202**, 917–935 (2013).

21. J. Gemperle, T. S. Harrison, C. Flett, A. D. Adamson, P. T. Caswell, On demand expression control of endogenous genes with DExCon, DExogron and LUXon reveals differential dynamics of Rab11 family members. *eLife* **11**, 1–39 (2022).

22. B. Wilson, C. Flett, J. Gemperle, C. Lawless, M. Hartshorn, E. Hinde, T. Harrison, M. Chastney, S. Taylor, J. Allen, J. C. Norman, T. Zacharchenko, P. T. Caswell, Proximity labelling identifies pro-migratory endocytic recycling cargo and machinery of the Rab4 and Rab11 families. *J. Cell Sci.* **136**, jcs260468 (2023).

23. J. R. Goldenring, K. R. Shen, H. D. Vaughan, I. M. Modlin, Identification of a small GTP-binding protein, Rab25, expressed in the gastrointestinal mucosa, kidney, and lung. *J. Biol. Chem.* **268**, 18419–18422 (1993).

24. B. Y. Jeong, K. H. Cho, K. J. Jeong, Y. Y. Park, J. M. Kim, S. Y. Rha, C. G. Park, G. B. Mills, J. H. Cheong, H. Y. Lee, Rab25 augments cancer cell invasiveness through a β1 integrin/EGFR/VEGF-A/Snail signaling axis and expression of fascin. *Exp. Mol. Med.* **50**, e435 (2018).

25. K. W. Cheng, J. P. Lahad, W. Kuo, A. Lapuk, K. Yamada, N. Auersperg, J. Liu, K. Smith-McCune, K. H. Lu, D. Fishman, J. W. Gray, G. B. Mills, The RAB25 small GTPase determines aggressiveness of ovarian and breast cancers. *Nat. Med.* **10**, 1251–1256 (2004).

26. M. A. Dozynkiewicz, N. B. Jamieson, I. Macpherson, J. Grindlay, P. V. E. van den Berghe, A. von Thun, J. P. Morton, C. Gourley, P. Timpson, C. Nixon, C. J. McKay, R. Carter, D. Strachan, K. Anderson, O. J. Sansom, P. T. Caswell, J. C. Norman, Rab25 and CLIC3 collaborate to promote integrin recycling from late endosomes/lysosomes and drive cancer progression. *Dev. Cell* **22**, 131–145 (2012).

27. P. van Bergeijk, M. Adrian, C. C. Hoogenraad, L. C. Kapitein, Optogenetic control of organelle transport and positioning. *Nature* **518**, 111–114 (2015).

28. C. C. Hoogenraad, A. Akhmanova, S. A. Howell, B. R. Dortland, C. I. De Zeeuw, R. Willemsen, P. Visser, F. Grosveld, N. Galjart, Mammalian Golgi-associated Bicaudal-D2 functions in the dynein-dynactin pathway by interacting with these complexes. *EMBO J.* **20**, 4041–4054 (2001).

29. W. Nijenhuis, M. M. P. van Grinsven, L. C. Kapitein, An optimized toolbox for the optogenetic control of intracellular transport. *J. Cell Biol.* **219**, e201907149 (2020).

30. V. I. P. Keizer, S. Grosse-Holz, M. Woringer, L. Zambon, K. Aizel, M. Bongaerts, F. Delille, L. Kolar-Znika, V. F. Scolari, S. Hoffmann, E. J. Banigan, L. A. Mirny, M. Dahan, D. Fachinetti, A. Coulon, Live-cell micromanipulation of a genomic locus reveals interphase chromatin mechanics. *Science* **377**, 489–495 (2022).

31. D. Liße, C. Monzel, C. Vicario, J. Manzi, I. Maurin, M. Coppey, J. Piehler, M. Dahan, Engineered ferritin for magnetogenetic manipulation of proteins and organelles inside living cells. *Adv. Mater.* **29**, 1–7 (2017).

32. M. Kappen, J. Gemperle, E. Secret, J. Flesch, P. T. Caswell, M. Coppey, C. Menager, D. Lisse, J. Piehler, Biofunctional coating of synthetic magnetic nanoparticles enables magnetogenetic control of protein functions inside cells. bioRxiv 621314 [Preprint] (2024). https://doi.org/10.1101/2024.10.31.621314.

33. J. H. R. Hetmanski, M. C. Jones, F. Chunara, J. M. Schwartz, P. T. Caswell, Combinatorial mathematical modelling approaches to interrogate rear retraction dynamics in 3D cell migration. *PLOS Comput. Biol.* **17**, e1008213 (2021).

34. S. Shukla, A. Troitskaia, N. Swarna, B. K. Maity, M. Tjioe, C. S. Bookwalter, K. M. Trybus, Y. R. Chemla, P. R. Selvin, High-throughput force measurement of individual kinesin-1 motors during multi-motor transport. *Nanoscale* **14**, 12463–12475 (2022).

35. M. J. Schnitzer, K. Visscher, S. M. Block, Force production by single kinesin motors. *Nat. Cell Biol.* **2**, 718–723 (2000).

36. R. A. Cross, Myosin's mechanical ratchet. *Proc. Natl. Acad. Sci. U.S.A.* **103**, 8911–8912 (2006).

37. M. Sittewelle, S. J. Royle, Passive diffusion accounts for the majority of intracellular nanovesicle transport. *Life Sci. Alliance* **7**, e202302406 (2024).

38. B. Schlierf, G. H. Fey, J. Hauber, G. M. Hocke, O. Rosorius, Rab11b is essential for recycling of transferrin to the plasma membrane. *Exp. Cell Res.* **259**, 257–265 (2000).

39. F. Matsuzaki, M. Shirane, M. Matsumoto, K. I. Nakayama, Protrudin serves as an adaptor molecule that connects KIF5 and its cargoes in vesicular transport during process formation. *Mol. Biol. Cell* **22**, 4602–4620 (2011).

40. R. Kaukonen, G. Jacquemet, H. Hamidi, J. Ivaska, Cell-derived matrices for studying cell proliferation and directional migration in a complex 3D microenvironment. *Nat. Protoc.* **12**, 2376–2390 (2017).

41. J. H. R. Hetmanski, H. de Belly, I. Busnelli, T. Waring, R. V. Nair, V. Sokleva, O. Dobre, A. Cameron, N. Gauthier, C. Lamaze, J. Swift, A. del Campo, T. Starborg, T. Zech, J. G. Goetz, E. K. Paluch, J. M. Schwartz, P. T. Caswell, Membrane tension orchestrates rear retraction in matrix-directed cell migration. *Dev. Cell* **51**, 460–475.e10 (2019).

42. P. T. Caswell, T. Zech, Actin-based cell protrusion in a 3D matrix. *Trends Cell Biol.* **28**, 823–834 (2018).

43. Y. Han, E. Eppinger, I. G. Schuster, L. U. Weigand, X. Liang, E. Kremmer, C. Peschel, A. M. Krackhardt, Formin-like 1 (FMNL1) is regulated by N-terminal myristoylation and induces polarized membrane blebbing. *J. Biol. Chem.* **284**, 33409–33417 (2009).

44. S. Yayoshi-Yamamoto, I. Taniuchi, T. Watanabe, FRL, a novel formin-related protein, binds to Rac and regulates cell motility and survival of macrophages. *Mol. Cell. Biol.* **20**, 6872–6881 (2000).

45. A. Nürnberg, T. Kitzing, R. Grosse, Nucleating actin for invasion. *Nat. Rev. Cancer* **11**, 117–187 (2011).

46. S. A. Eisler, F. Curado, G. Link, S. Schulz, M. Noack, M. Steinke, M. A. Olayioye, A. Hausser, A rho signaling network links microtubules to PKD controlled carrier transport to focal adhesions. *eLife* **7**, e35907 (2018).

47. C. Gaston, S. De Beco, B. Doss, M. Pan, E. Gauquelin, J. D'Alessandro, C. T. Lim, B. Ladoux, D. Delacour, EpCAM promotes endosomal modulation of the cortical RhoA zone for epithelial organization. *Nat. Commun.* **12**, 2226 (2021).

48. V. Vassilev, A. Platek, S. Hiver, H. Enomoto, M. Takeichi, Catenins steer cell migration via stabilization of front-rear polarity. *Dev. Cell* **43**, 463–479.e5 (2017).

49. J. Alanko, J. Ivaska, Endosomes: Emerging platforms for integrin-mediated FAK signalling. *Trends Cell Biol.* **26**, 391–398 (2016).

50. H. Bagci, N. Sriskandarajah, A. Robert, J. Boulais, I. E. Elkholi, V. Tran, Z. Y. Lin, M. P. Thibault, N. Dubé, D. Faubert, D. R. Hipfner, A. C. Gingras, J. F. Côté, Mapping the proximity interaction network of the Rho-family GTPases reveals signalling pathways and regulatory mechanisms. *Nat. Cell Biol.* **22**, 120–134 (2020).

51. T. S. Gomez, K. Kumar, R. B. Medeiros, Y. Shimizu, P. J. Leibson, D. D. D. Billadeau, Formins regulate the actin-related protein 2/3 complex-independent polarization of the centrosome to the immunological synapse. *Immunity* **26**, 177–190 (2007).

52. E. K. Mahlandt, J. J. G. Arts, W. J. van der Meer, F. H. van der Linden, S. Tol, J. D. van Buul, T. W. J. Gadella, J. Goedhart, Visualizing endogenous Rho activity with an improved localization-based, genetically encoded biosensor. *J. Cell Sci.* **134**, jcs258823 (2021).

53. P. A. J. Muller, P. T. Caswell, B. Doyle, M. P. Iwanicki, E. H. Tan, S. Karim, N. Lukashchuk, D. A. Gillespie, R. L. Ludwig, P. Gosselin, A. Cromer, J. S. Brugge, O. J. Sansom, J. C. Norman, K. H. Vousden, Mutant p53 drives invasion by promoting integrin recycling. *Cell* **139**, 1327–1341 (2009).

54. P. T. Caswell, M. Chan, A. J. Lindsay, M. W. McCaffrey, D. Boettiger, J. C. Norman, Rab-coupling protein coordinates recycling of α5β1 integrin and EGFR1 to promote cell migration in 3D microenvironments. *J. Cell Biol.* **183**, 143–155 (2008).

55. M. Bongaerts, K. Aizel, E. Secret, A. Jan, T. Nahar, F. Raudzus, S. Neumann, N. Telling, R. Heumann, J. M. Siaugue, C. Ménager, J. Fresnais, C. Villard, A. El Haj, J. Piehler, M. A. Gates, M. Coppey, Parallelized manipulation of adherent living cells by magnetic nanoparticles-mediated forces. *Int. J. Mol. Sci.* **21**, 6560 (2020).

56. F. Etoc, C. Vicario, D. Lisse, J.-M. M. Siaugue, J. Piehler, M. Coppey, M. Dahan, Magnetogenetic control of protein gradients inside living cells with high spatial and temporal resolution. *Nano Lett.* **15**, 3487–3494 (2015).

57. M. B. Steketee, S. N. Moysidis, X. Jin, J. E. Weinstein, W. Pita-thomas, H. B. Raju, S. Iqbal, J. L. Goldberg, Nanoparticle-mediated signaling endosome localization regulates growth cone motility and neurite growth. *Proc. Natl. Acad. Sci. U.S.A.* **108**, 19042–19047 (2011).

58. K. Dhillon, K. Aizel, T. J. Broomhall, E. Secret, T. Goodman, M. Rotherham, N. Telling, J. M. Siaugue, C. Ménager, J. Fresnais, M. Coppey, A. J. El Haj, M. A. Gates, Directional control of neurite outgrowth: Emerging technologies for Parkinson's disease using magnetic nanoparticles and magnetic field gradients. *J. R. Soc. Interface* **19**, 20220576 (2022).

59. N. Alzahofi, T. Welz, C. L. Robinson, E. L. Page, D. A. Briggs, A. K. Stainthorp, J. Reekes, D. A. Elbe, F. Straub, W. W. Kallemeijn, E. W. Tate, P. S. Goff, E. V. Sviderskaya, M. Cantero, L. Montoliu, F. Nedelec, A. K. Miles, M. Bailly, E. Kerkhoff, A. N. Hume, Rab27a co-ordinates actin-dependent transport by controlling organelle-associated motors and track assembly proteins. *Nat. Commun.* **11**, 3495 (2020).

60. S. Phuyal, P. Romani, S. Dupont, H. Farhan, Mechanobiology of organelles: Illuminating their roles in mechanosensing and mechanotransduction. *Trends Cell Biol.* **33**, 1049–1061 (2023).

61. B. Dehapiot, R. Clément, H. Alégot, G. Gazsó-Gerhát, J. M. Philippe, T. Lecuit, Assembly of a persistent apical actin network by the formin Frl/Fmnl tunes epithelial cell deformability. *Nat. Cell Biol.* **22**, 791–802 (2020).

62. O. Ossipova, K. Kim, B. B. Lake, K. Itoh, A. Ioannou, S. Y. Sokol, Role of Rab11 in planar cell polarity and apical constriction during vertebrate neural tube closure. *Nat. Commun.* **5**, 3734 (2014).

63. P. M. Willoughby, M. Allen, J. Yu, R. Korytnikov, T. Chen, Y. Liu, I. So, N. Macpherson, J. A. Mitchell, R. Fernandez-Gonzalez, A. E. E. Bruce, The recycling endosome protein Rab25 coordinates collective cell movements in the zebrafish surface epithelium. *eLife* **10**, e66060 (2021).

64. S. Kühn, C. Erdmann, F. Kage, J. Block, L. Schwenkmezger, A. Steffen, K. Rottner, M. Geyer, The structure of FMNL2–Cdc42 yields insights into the mechanism of lamellipodia and filopodia formation. *Nat. Commun.* **6**, 7088 (2015).

65. K. Zaoui, S. Honoré, D. Isnardon, D. Braguer, A. Badache, Memo-RhoA-mDia1 signaling controls microtubules, the actin network, and adhesion site formation in migrating cells. *J. Cell Biol.* **183**, 401–408 (2008).

66. B. Sönnichsen, S. De Renzis, E. Nielsen, J. Rietdorf, M. Zerial, Distinct membrane domains on endosomes in the recycling pathway visualized by multicolor imaging of Rab4, Rab5, and Rab11. *J. Cell Biol.* **149**, 901–914 (2000).

67. M. Padilla-Rodriguez, S. Parker, D. Adams, T. Westerling, J. Puleo, A. Watson, S. Hill, M. Noon, R. Gaudin, J. Aaron, D. Tong, D. Roe, B. Knudsen, G. Mouneimne, The actin cytoskeletal architecture of estrogen receptor positive breast cancer cells suppresses invasion. *Nat. Commun.* **9**, 2980 (2018).

68. E. Kowarz, D. Löscher, R. Marschalek, Optimized Sleeping Beauty transposons rapidly generate stable transgenic cell lines. *Biotechnol. J.* **10**, 647–653 (2015).

69. F. Kawano, H. Suzuki, A. Furuya, M. Sato, Engineered pairs of distinct photoswitches for optogenetic control of cellular proteins. *Nat. Commun.* **6**, 6256 (2015).

70. E. W. Miller, S. D. Blystone, The carboxy-terminus of the formin FMNL1ɣ bundles actin to potentiate adenocarcinoma migration. *J. Cell. Biochem.* **120**, 14383–14404 (2019).

71. H. Li, K. A. Beckman, V. Pessino, B. Huang, J. S. Weissman, M. D. Leonetti, Design and specificity of long ssDNA donors for CRISPR-based knock-in. bioRxiv 178905 [Preprint] (2017). https://doi.org/10.1101/178905.

72. H. Bennett, E. Aguilar-Martinez, A. D. Adamson, CRISPR-mediated knock-in in the mouse embryo using long single stranded DNA donors synthesised by biotinylated PCR. *Methods* **191**, 3–14 (2021).

73. M. R. Miller, E. W. Miller, S. D. Blystone, Non-canonical activity of the podosomal formin FMNL1γ supports immune cell migration. *J. Cell Sci.* **130**, 1730–1739 (2017).

74. E. Cukierman, R. Pankov, D. Stevens, K. Yamada, Taking cell-matrix adhesions to the third dimension. *Science* **294**, 1708–1712 (2001).

75. F. Piccinini, A. Kiss, P. Horvath, CellTracker (not only) for dummies. *Bioinformatics* **32**, 955–957 (2016).

76. Q. Tseng, E. Duchemin-Pelletier, A. Deshiere, M. Balland, H. Guilloud, O. Filhol, M. Théry, Spatial organization of the extracellular matrix regulates cell-cell junction positioning. *Proc. Natl. Acad. Sci. U.S.A.* **109**, 1506–1511 (2012).

77. M. A. Jakobs, A. Dimitracopoulos, K. Franze, Kymobutler, a deep learning software for automated kymograph analysis. *eLife* **8**, e42288 (2019).

78. I. Belevich, M. Joensuu, D. Kumar, H. Vihinen, E. Jokitalo, Microscopy image browser: A platform for segmentation and analysis of multidimensional datasets. *PLOS Biol.* **14**, e1002340 (2016).

79. M. H. H. Nørholm, A mutant Pfu DNA polymerase designed for advanced uracil-excision DNA engineering. *BMC Biotechnol.* **10**, 21 (2010).

80. L. A. Gross, G. S. Baird, R. C. Hoffman, K. K. Baldridge, R. Y. Tsien, The structure of the chromophore within DsRed, a red fluorescent protein from coral. *Proc. Natl. Acad. Sci. U.S.A.* **97**, 11990–11995 (2000).
